# First-Trimester Abortion Complications: Simulation Cases for OB/GYN Residents in Sepsis and Hemorrhage

**DOI:** 10.15766/mep_2374-8265.10995

**Published:** 2020-10-16

**Authors:** Armide Storey, Katharine White, Kelly Treder, Elisabeth Woodhams, Shannon Bell, Rachel Cannon

**Affiliations:** 1 Fourth-Year Medical Student, Boston University School of Medicine; 2 Associate Professor, Obstetrics and Gynecology, Boston University School of Medicine; 3 Instructor, Obstetrics and Gynecology, Boston University School of Medicine; 4 Assistant Professor, Obstetrics and Gynecology, Boston University School of Medicine

**Keywords:** Simulation, Abortion, Uterine Aspiration, Complication, Infection, Sepsis, Hemorrhage, Interdisciplinary Studies, Resident Education

## Abstract

**Introduction:**

Serious complications associated with first-trimester abortions are rare. The US mortality rate for these procedures is 0.7 per 100,000, primarily due to infection and hemorrhage. While complications are unlikely to arise during training, residents must be prepared to manage them in practice. To address this, we developed a 2-hour simulation-based abortion complication curriculum for OB/GYN resident learners.

**Methods:**

OB/GYN residents participated in three sessions: a case-based didactic reviewing institutional aspiration abortion practice and preop preparation; an in-vivo aspiration abortion hemorrhage simulation; and an interdepartmental postabortal sepsis simulation. Participants completed surveys before and after their participation that evaluated clinical knowledge, and self-rated competence in, and preparedness for, managing first-trimester abortion complications.

**Results:**

Resident learners (*N* = 26) represented all four classes of OB/GYN residents. Residents initially showed stronger clinical knowledge in managing postabortal hemorrhage than sepsis (90% vs. 62%, *p* < .001). Clinical knowledge improved following the sepsis simulation (62% to 91%, *p* < .001), and remained strong but unchanged after the hemorrhage simulation (90% to 87%, *p* = .3). Resident self-assessments of competence and preparedness were significantly improved after both the hemorrhage (*p* = .006) and sepsis (*p* = .002) simulations. Learners reported that the simulation increased their level of comfort in managing these complications in their future practice.

**Discussion:**

Postabortal hemorrhage and sepsis simulations increased OB/GYN residents' knowledge, comfort, and preparedness for managing rare complications of first-trimester abortions. In-vivo simulation and interdepartmental collaboration were novel aspects of these simulations that may facilitate increased preparedness and management skills.

## Educational Objectives

By the end of this activity, learners will be able to:

1. Demonstrate improved recognition of sepsis in a patient presenting for urgent care after medication abortion.
2. Demonstrate improved knowledge of the differential diagnosis of sepsis following abortion.
3. Demonstrate improved knowledge of the most common etiologies of hemorrhage at the time of aspiration abortion.
4. Develop a plan to evaluate and manage the most common etiologies of hemorrhage and sepsis as first-trimester abortion complications.
5. Demonstrate effective communication skills and workflow management with coresidents and colleagues from different disciplines in evaluating an emergency scenario and transferring a patient to an escalated level of care.

## Introduction

Abortions are one of the most common medical procedures in the US, and nearly one in four women will have an abortion by the age of 45.^1^ The procedure is exceptionally safe and serious complications are rare, with a mortality rate of 0.7 per 100,000. This small risk is further reduced earlier in pregnancy, with a the death rate of 0.3 per 100,000 for pregnancies at 8 weeks gestation or less.^2,3^ The ACGME accreditation of an OB/GYN residency program requires an “established curriculum for family planning, including for complications of abortions and provisions for the opportunity for direct procedural training in terminations of pregnancy for those residents who desire it.”^4^ Despite this requirement, only 64% of OB/GYN residency programs provide routine, scheduled training in family planning.^5^

Even with robust exposure to family planning, resident learners are unlikely to manage serious abortion complications such as hemorrhage and sepsis given their relative rarity (hemorrhage and infection each occur following fewer than 1% of abortions).^6,7^ While uncommon, these complications are potentially of great consequence, and providers must be prepared to recognize and address these efficiently and confidently in their future practice. Appropriately exposing resident learners to real-world training opportunities for rare events poses a challenge to graduate medical educators. To address this challenge, we developed a simulation-based abortion complication curriculum for resident learners.

Simulation training has been described as an ethical imperative in protecting patient safety and well-being.^8^ Practicing with simulation-based models has been shown to improve provider skill^9–11^ and confidence,^12,13^ as well as improve patient outcomes^14,15^ and reduce health care costs.^16^ Simulation‐based technical and nontechnical skills have been demonstrated to be transferable to the patient‐based setting.^17,18^

Given the similar impact of low-fidelity models when compared to more sophisticated models,^19^ we developed a low-fidelity simulation to prioritize a low-cost, easily reproducible learning experience. The primary goals of this simulation were to improve learner knowledge, comfort, and preparedness for managing rare complications of first-trimester abortions. This simulation also provided a valuable opportunity for our department to simulate a medical emergency in our gynecologic procedure unit and identify potential barriers to safe and efficient patient care. To our knowledge, there are two abortion complication simulations available via *MedEdPORTAL*, a sepsis simulation for emergency medicine clinicians^20^ and a hemorrhage simulation utilizing pitaya fruit.^21^ To further contribute to this literature, we developed simulations that were novel in their scope and design, incorporating a multidisciplinary, in situ approach. These simulations allowed residents not only to practice skills for scenarios they may not have the opportunity to experience in their training, but also to become familiar with emergency workflow in the clinical setting and practice collaborating across disciplines to care for a patient in crisis.

## Methods

### Development

We created an abortion complication simulation curriculum for OB/GYN residents, who typically have little to no hands-on experience managing hemorrhage and sepsis as complications of first-trimester abortion because of their rarity. The simulations were modeled on cases seen at our institution, using open-access materials developed by the Training in Early Abortion for Comprehensive Healthcare Training Program as a framework.^22^ Prerequisite knowledge for the curriculum included medical school training and basic knowledge of first-trimester abortion techniques as well as basic knowledge of the pathophysiology of hemorrhage and sepsis.

### Equipment/Environment

We used a simulation mannequin available through our institution (Victoria, Gaumard S2200, a maternal and neonatal birthing simulator) as the patient in these simulations. Learners were able to place an IV, monitor vitals (controlled by faculty), perform a pelvic exam, and a mock uterine aspiration on this mannequin. The sepsis simulation (Appendix A) took place in our simulation center set up as a simulated emergency department (ED) and the hemorrhage simulation (Appendix B) took place in situ in our institution's ambulatory gynecologic procedure unit. Each simulation had specific equipment needs (Appendices A–C). Faculty verbally reported imaging findings and bleeding was simulated with fake blood-saturated underpads (Chux).

### Personnel

Both simulations were overseen by at least two clinical faculty, one whose role was to respond to and prompt learners, and the other simply to observe and intermittently replace the underpads to simulate ongoing bleeding. Learners volunteered for roles to be played during the simulations: attending, resident, medical student, patient support person, and observers. The resident learners participated in the simulation alongside case-specific roles played by nonlearners. For the sepsis simulation (Appendix A), an emergency medicine physician played himself and an OB/GYN faculty member played the role of the ED nurse. For the hemorrhage simulation (Appendix B), an anesthesiologist, nurse, medical assistant, and operating room (OR) colleague played themselves. These participants worked regularly in the clinical setting of our outpatient gynecology procedure unit. To prepare for their various nonlearner roles, facilitators met with the team that developed the curriculum to walk through the cases (Appendices A and B) and addressed any potential areas of confusion or concern prior to beginning the simulation.

### Implementation

We performed a prospective pilot study of this simulation curriculum with OB/GYN residents of Boston Medical Center, an urban academic medical center, during three protected educational sessions. The curriculum began with a 2-hour case-based didactic lecture (Appendix D) with an emphasis on institutional practices, preoperative evaluation and preparation, and potential challenges and complications. The following two sessions were simulations; one on sepsis (Appendix A) and one on hemorrhage (Appendix B). The simulation sessions were 2 hours long and led by faculty in the OB/GYN department. Within each session, the simulation was run twice to accommodate two small groups of learners. Prior to each simulation, learners were given a brief description of the case and self-assigned roles.

The sepsis simulation (Appendix A) was conducted in the hospital's simulation center (Appendix C). The scenario was a consult for a patient presenting to the ED 2 days after a medication abortion at an outside clinic with heavy bleeding at home. Learners received sign-out from an emergency medicine physician and worked with them on the development of a management plan. The ED nurse caring for the patient was present. Learners interviewed the patient while faculty, observing through one-way glass, responded via remote microphone. Learners performed an exam on the mannequin which revealed fake blood-stained disposable underpads (Appendix C). The simulation provided opportunities for learners to identify the signs, symptoms, and differential diagnosis for sepsis, evaluate vital signs and laboratory results, identify the need for appropriate diagnostic tests and imaging, practice initial management of sepsis including resuscitation and antibiotic therapy, and practice decision making regarding aspiration abortion in the ED versus the OR (Appendix E).

The hemorrhage simulation (Appendix B) was conducted in the ambulatory gynecologic procedure unit, which is the setting of nearly all of the abortion care in our institution (Appendix C). The scenario was a patient presenting for a first-trimester aspiration abortion. Learners performed a mock uterine aspiration with support of anesthesia, nursing, and medical assistant colleagues as per hospital policy. Observing faculty reported the status of the patient and her ongoing bleeding to the learners using verbal cues. Bleeding was simulated with increasingly blood-saturated underpads under the mannequin (Appendix C). The simulation provided opportunities for the learners to correctly identify hemorrhage; recognize the need to transition from manual to electric suction; evaluate the most common etiologies of hemorrhage; identify the utility of ultrasound in diagnosis and management; evaluate for cervical laceration; utilize uterine massage, uterotonics, and Foley catheter tamponade; and identify the need for and prepare for transfer to the OR (Appendix F). Transfer to the OR in this simulation involved direct communication with the OR staff for preparation, moving the mannequin to a stretcher, preparing it for transport with an oxygen tank and a cardiac monitor, and ultimately bringing the mannequin to the doors of the OR.

### Debriefing

A debrief with faculty, staff, and learners followed each simulation (Appendices G and H). Faculty facilitated the conversation and learners had the opportunity to reflect on past clinical experiences and their strengths and weaknesses as a team. For each simulation, faculty reviewed the clinical pearls from the case, answered any learner questions, and reviewed the correct responses to the knowledge questions asked in the pre- and postsurvey.

For the sepsis debrief, faculty also reviewed a didactic presentation about the recognition and management of sepsis in the acute setting (Appendix I). We chose to review this information more formally with learners because of the unique opportunity for interdepartmental teaching with our emergency medicine colleague who cares for a much higher volume of sepsis cases. We also expected learners to feel less familiar with the management of sepsis than of hemorrhage given their familiarity with obstetric hemorrhage.

### Assessment

Standards of competence for each simulation (Appendices E and F) were developed using the Interprofessional Education Collaborative's Core Competencies for Interprofessional Collaborative Practice as a model.^23^ We assessed changes in learners' clinical knowledge and self-assessment of preparedness using pre- and postsurveys administered before and after each simulation (Appendices J and K), adapted from open-access materials developed by the Training in Early Abortion for Comprehensive Healthcare Training Program and published in *MedEdPORTAL*.^24^ Learners self-assessed their ability to recognize and manage abortion complications, as well as their readiness to take leadership and remain calm when caring for these patients. We included in the postsurvey an evaluation of the educational experience.

A single evaluator (Armide Storey) scored all de-identified pre- and postsurveys. We performed descriptive analysis of the data with means and standard deviations of the knowledge, competence, and preparedness pre- and postsurveys. We hypothesized that learners would have higher baseline knowledge in hemorrhage than in sepsis because of their experience in managing obstetric hemorrhage. We hypothesized that the simulations would increase clinical knowledge and confidence in sepsis and hemorrhage management. We conservatively performed two-sided paired *t* tests of the mean of the differences between pre- and postassessments of knowledge, competence, and preparedness, and between the sepsis and hemorrhage knowledge preassessments. Statistical analyses were conducted using R version 3.5.2 (2018-12-20).

## Results

Resident learners (*N* = 26) represented all four classes of OB/GYN residents, and some residents participated in one or both simulations (seven PGY 1s, five PGY 2s, five PGY 3s, and nine PGY 4s). Members of the OB/GYN department, including attendings and fellows, facilitated the simulation. This was the first time this type of simulation was performed at our institution. Residents initially showed stronger clinical knowledge in managing postabortal hemorrhage than sepsis (90% vs. 62%, *p* < .001).

Twelve learners attended the hemorrhage simulation, and 11 completed both pre- and postsurveys. Prior to the simulation, the average knowledge presurvey score was 90%, and remained stable following the simulation (90% to 87%, *p* = .3). We noticed a pattern in knowledge change for the five learners whose scores decreased: all changed a correct to an incorrect answer to the question, “What is the most common cause of bleeding in first-trimester uterine aspiration?” The correct answer was retained tissue; all changed to the incorrect answer uterine atony. Resident self-assessments of competence and preparedness improved after the simulation (*p* = .006).

Fourteen learners attended the sepsis simulation, and 10 completed both pre- and postsurveys. Prior to the simulation, the average knowledge presurvey score was 62%, and the knowledge score improved to 91% following the simulation (*p* < .001). Resident self-assessments of competence and preparedness improved after the simulation (*p* = .002).

While we did not perform rigorous qualitative review on the survey feedback, participants were invited to share their reflections, which were overwhelmingly positive: “Very informative;” “Loved it;” “This was awesome;” and, “Cool inclusion of OR staff.” Residents also shared how this simulation would change their future practice: “This sim made me think about how important it is to be systematic in your assessment and how to physically get the things you need.” In the future they will “pay more attention to (and understand) lactate;” “Call for help early, delegate tasks, think step-wise;” “Ask for specific equipment;” “Use all [the] tools available in clinic;” and “Utilize foley bulbs, [and] transition from manual to electric suction.”

## Discussion

We created this simulation for abortion care to provide resident learners the opportunity to recognize and manage two complications of first-trimester abortion: hemorrhage and sepsis. These scenarios represented rare but potentially serious clinical challenges that physicians must be familiar with in order to provide timely and appropriate care. Overall, these simulations have achieved our primary objective of improving learner knowledge, comfort, and perception of preparedness for managing complications of first-trimester abortions. Additionally, the curriculum was well-received by the residents, whose responses were overwhelmingly positive when solicited for feedback. While simulation is a well-established educational modality in graduate medical education, these simulations were novel in their scope and design. To our knowledge, this was the first abortion complication simulation of its kind, incorporating a multidisciplinary approach and partially taking place in the setting in which these residents practice. These simulations incorporated an emergency medicine physician, anesthesiologist, members of the OR staff, nurses, and medical assistants. We also recognized the potential role for this simulation in interdisciplinary team trainings. The opportunity to practice robust communication and management skills under clinical pressure could have important applications beyond resident education.

Residents initially showed stronger clinical knowledge in managing postabortal hemorrhage than sepsis, which was unsurprising given the relatively high frequency of obstetric hemorrhage compared to sepsis in their general OB/GYN training. While we didn't have the power to assess differences across training levels, we found that upper level residents volunteered to participate in the senior levels roles of the simulation. We suspect this was due to confidence in their knowledge level and comfort around their peers. We hoped this simulation would break down some communication barriers for junior level residents as they practiced interacting with their peers and other hospital services. We were interested to see that all hemorrhage learners whose scores decreased incorrectly identified atony as the most common cause of bleeding when they had originally correctly named retained tissue as the leading cause. We suspect this was because the clinical simulation was an atony case, which we chose for both the ease of simulation and the higher complexity of management decisions required. In the future, we will include a more thorough review of the relative frequency of each cause of hemorrhage during the structured debrief.

We hypothesized that the improvement in knowledge, comfort, and preparedness was in part due to the believability of the experience, taking place in the clinic in which these residents work, alongside their colleagues who are playing themselves, and with the realistic visual cue of blood-stained underpads. We chose to use a sophisticated simulation mannequin that was available to us through our institution because our resident learners were familiar with this mannequin from their surgical training simulations. However, the bleeding was simulated with low-fidelity blood-soaked underpads and we recognized this simulation could easily be reproduced with a simple pelvic model for a complete, low-cost alternative.

Limitations of this study included that all participating residents were recruited from an OB/GYN residency program at a single institution with robust abortion training, which may limit the generalizability to other training programs. Reliance on self-assessed perception of preparedness and comfort may introduce a social desirability bias, though we attempted to mitigate this effect with deidentified pre- and postsurveys. Further, by relying on knowledge and self-reported comfort and preparedness data, we were unable to directly assess the skills-based learning objectives of this exercise. The curriculum learning objectives would be better assessed with a demonstration of learner competence using a graded rubric by a faculty observer, which is a potential future direction for this curriculum. Another limitation was the small sample size of our cohort, and we would be interested in running this simulation with a larger cohort including resident learners from different specialties who may manage patients following their abortions, such as emergency medicine, family medicine, and internal medicine. Despite these limitations, we were able to demonstrate a positive effect of the simulation curriculum on knowledge and self-assessed preparedness and comfort.

We believe that this simulation curriculum for first-trimester abortion complications could be easily incorporated into educational curricula as an effective solution to the challenge within graduate medical education of exposing residents to rare but important learning opportunities, including the postabortal complications of hemorrhage and sepsis.

## Appendices

Sepsis Simulation Case.docxHemorrhage Simulation Case.docxSimulation Images.docxPresimulation Didactic Lecture.pptxSepsis Critical Action Checklist.docxHemorrhage Critical Action Checklist.docxSepsis Debriefing Guide.docxHemorrhage Debriefing Guide.docxSepsis Postsimulation Debrief Didactic.pptxSepsis Pre-and Postsurvey.docxHemorrhage Pre-and Postsurvey.docx
All appendices are peer reviewed as integral parts of the Original Publication.
