# Supplementary material for: First-Trimester Abortion Complications: Simulation Cases for OB/GYN Residents in Sepsis and Hemorrhage
Source: MedEdPORTAL. 2020 Oct 16;16:10995. doi: 10.15766/mep_2374-8265.10995 (PMC7566226; doi:10.15766/mep_2374-8265.10995)
Supplement: Supplementary file 1 — Sepsis Simulation Case.docxHemorrhage Simulation Case.docxSimulation Images.docxPresimulation Didactic Lecture.pptxSepsis Critical Action Checklist.docxHemorrhage Critical Action Checklist.docxSepsis Debriefing Guide.docxHemorrhage Debriefing Guide.docxSepsis Postsimulation Debrief Didactic.pptxSepsis Pre-and Postsurvey.docxHemorrhage Pre-and Postsurvey.docx [file mep_2374-8265.10995-s001.zip › A. Sepsis Simulation Case.docx]

| **Appendix A: Sepsis Simulation Case Template**  **SIMULATION CASE TITLE: Assessment and management of sepsis following first-trimester abortion**  **AUTHORS: Armide Storey, BS, Katharine White MD, MPH, Kelly Treder, MD, MPH, Elisabeth Woodhams, MD, MSc, Shannon Bell, MD, Rachel Cannon, MD, MSc**  **LEARNER AUDIENCE: OB/GYN Residents** | |
| --- | --- |
| **PATIENT NAME: Maria**  **PATIENT AGE: 35**  **CHIEF COMPLAINT: Heavy Bleeding**  **PHYSICAL SETTING: Emergency Department** | |
|  | |
| **Brief narrative description of case** | The patient is a 35yo G3P1011 who presents to the emergency department via ambulance after heavy vaginal bleeding at home. She started a medication abortion at 6 weeks gestational age at an outside clinic 2 days prior. She is found to meet criteria for sepsis with possible shock and end organ damage. This simulation provides opportunities for learners to practice identification and management of sepsis after a medication abortion. |
| **Primary Learning Objectives** | By the end of this activity, learners will be able to:   1. Demonstrate improved recognition of sepsis in a patient presenting for urgent care after medication abortion 2. Demonstrate improved knowledge of the differential diagnosis of sepsis following abortion 3. Develop a plan to evaluate and manage the most common etiologies of sepsis as first-trimester abortion complications 4. Demonstrate effective communication skills and workflow management with co-residents and colleagues from different disciplines in evaluating an emergency scenario and transferring a patient to an escalated level of care |
| **Critical Actions** | 1. Ascertain complete history and physical from Emergency Department (ED) report 2. Order appropriate labs for a patient presenting to the ED for complications due to a medication abortion and meeting criteria for sepsis with possible shock and end organ damage    1. CBC    2. CMP    3. Urine pregnancy test    4. Lactate    5. Venous blood gas    6. Coagulation panel    7. Type and screen    8. Blood culture 3. Perform bimanual and speculum exam to evaluate uterine bleeding 4. Review the differential diagnosis    1. Septic abortion    2. Endometritis    3. PID/TOA    4. Hemorrhagic shock 5. Perform bedside ultrasound to evaluate for retained products of conception 6. Recognize worsening vital signs: tachycardia, hypotension 7. Prepare for resuscitation efforts with two large bore IVs and requesting crossmatched units of blood 8. Work with ED colleagues to follow hospital sepsis protocols re: initiation of vasopressor support if BP refractory to IV fluids 9. Prepare for and perform immediate manual vacuum aspiration    1. Recognize the importance of performing the procedure in the trauma bay, which is faster than transferring the unstable patient to the operating room    2. Have the following supplies available:       1. MVA supplies       2. Dilation and Evacuation kit       3. Uterotonics including Misoprostol, Carboprost, Methylergonovine (Methergine)       4. Anesthesia/sedation (fentanyl/midazolam) 10. Prepare disposition plan     1. CT abdomen to be obtained in the ED prior to transfer pending stability     2. Consult ICU 11. Review plan for antibiotics     1. Broad spectrum antibiotics: clindamycin and vancomycin     2. Consult Infectious Disease 12. Demonstrate effective communication skills and workflow management with co-residents and emergency medicine colleagues |
| **Learner Preparation or Prework** | Learners participate in a two-hour case-based didactic lecture prior to the simulation with an emphasis on institutional practices, preoperative evaluation and preparation, and potential challenges and complications (Appendix D).  Prior to entering the simulation, learners are told “You were just paged for an emergency department consult for heavy bleeding after a medication abortion.” |
| **Equipment** | Simulation Mannequin  “Blood” stained underpads (Chux)  Simulated IV fluids  Simulated IV antibiotics  Simulated bedside ultrasound  Dilation and Evacuation kit  Manual Vacuum Aspiration with varying cannula sizes  Vitals monitor |

| Initial Presentation | | | |
| --- | --- | --- | --- |
| **Initial vital signs** | BP: 108/74 HR: 115 RR: 22 SpO2: 98 Temp: 95.3 | | |
| **Overall Setting and Appearance** | The simulation begins with learners calling the emergency medicine doctor for report on the patient. When the residents come to see the patient in the emergency room, they see an obstetric mannequin lying in a hospital bed. She is dressed in a patient gown with IV in place. | | |
| **Confederates (e.g., standardized participants) and their roles in the room at case start** | Registered nurse (RN) confederate: Faculty member playing RN support role  Emergency Medicine confederate: Emergency Medicine MD playing regular role  Facilitator 1: Attending level physician. Adjusts vital signs, reports patient status, and provided labs/images as the case progresses.  Facilitator 2: Observes, replaces underpads to simulate ongoing bleeding. | | |
| **Learner Roles** | Gynecology Attending  Gynecology Resident  Medical Student  Patient’s family member with them in the ED  Observers | | |
| **HPI** | HPI from Emergency Department (ED) report:  35yo G3P1011 who started a medication abortion at 6 weeks at an outside clinic 2 days ago. Received mifepristone 2 days ago and took misoprostol 1 day ago at home. Presents to ED by ambulance, reports very heavy bleeding at home. EMS reports bucket of blood next to the patient.  ROS: Positive for fever and chills, nausea and an episode of emesis, and vaginal spotting and discharge  Denies urinary symptoms, flank pain, changes in bowel movements, and vaginal itching.  Initial vital signs on arrival to the ED:  Temperature = 95.3 degrees Fahrenheit  Heart Rate = 115 beats per minute  Blood Pressure = 108/74 mmHg  Respiratory Rate = 22 breaths per minute  Oxygen Saturation = 98% on room air  Learner must ask for:  Labs are pending.  Bedside FAST exam is unremarkable. | | |
| **Past Medical/Obstetric/Surgical History** | **Medications** | **Allergies** | **Family History** |
| C-section 3 years prior  Ectopic pregnancy treated with methotrexate 1 year prior | none | NKDA | No applicable family history |
| **Physical Examination per ED physician report** | | | |
| **General** | Uncomfortable female lying supine in the gurney holding her low abdomen with both hands | | |
| **Lungs** | Tachypnea, otherwise unremarkable | | |
| **Cardiovascular** | Tachycardia, otherwise unremarkable | | |
| **Abdomen** | Bilateral low abdominal tenderness with voluntary guarding in the right lower quadrant greater than the left lower quadrant | | |
| **GU per learner’s exam** | External genitalia without erythema, exudate or discharge. Vaginal vault is without discharge. Cervix is 1cm dilated. Uterus is noted to be approximately 8wk in size, anteverted, and mobile. There is bleeding. | | |

| Instructor Notes – Changes and CASE Branch Points | | |
| --- | --- | --- |
| **Intervention / Time point** | **Change in Case** | **Additional Information** |
| Learner calls Emergency Department (ED) for patient report | ED physician gives report per H&P above, but does not report GU exam |  |
| Learner asks if labs have been drawn | ED physician reports a CBC and CMP have been drawn and results are pending |  |
|  | If not -> ED physician volunteers information |  |
| Learner asks for urine pregnancy test, coagulation panel, venous blood gas, lactate, and type and screen to be drawn | If not -> ED physician asks “are they any other labs you’d like to collect?” |  |
|  | If still no -> ED physician says “We are collecting a urine pregnancy test, coagulation panel, venous blood gas, lactate, and type and screen now.” |  |
| Learner asks if imaging has been done | ED physician says a bedside ultrasound (US) was unrevealing |  |
| Learner asks for more details regarding the US | ED physician reports that a FAST scan is negative |  |
|  | If not -> ED physician volunteers that the US was a FAST scan |  |
| Learner goes to ED to examine patient | ED nurse is placing an IV |  |
| Learner asks patient history | Facilitator 1 responds as patient “I am in pain and still bleeding.” She is somewhat delirious and unable to continue a train of thought. |  |
| Learner performs bimanual exam | Facilitator 1 responds “On bimanual exam you feel 1cm dilation and an anteverted, mobile uterus about 6wk in size.” | Learner sees underpad under patient with mild blood stain |
|  | If not -> facilitator 1 asks “will you be performing a bimanual exam?” |  |
| Learner performs speculum exam | Facilitator 1 responds “You see blood pooling, approximately 150cc.” | Facilitator 2 replaces underpad with pad stained with more blood |
|  | If not -> facilitator 1 asks “will you be performing a speculum exam?” |  |
| Learner notes new vital signs | Vitals monitor shows HR 130, BP 90/50, T 98.7, O2 94% |  |
|  | If not -> facilitator 1 asks “Have you seen her vitals?” |  |
| ED nurse places patient on 2L supplemental oxygen via nasal canula |  |  |
| Learner asks if the patient labs have returned | Facilitator 1 says “Labs are back and show:  WBC 25 with mild left shift  Hgb 10.9  Cr 0.8  Lactate 2.4  O negative blood type” |  |
|  | If not -> facilitator 1 asks “Have you seen the lab results?” |  |
| Learner talks with ED physician | Learner and ED physician discuss:   - Resuscitation efforts, including 2 large bore IVs with fluids - Drawing blood cultures - Sending crossmatch for 2 units | If not -> ED physician asks learner for case update and prompts for discussion content |
| Learner calls on call GYN attending (played by another learner) to review case | Learner asks attending to come to bedside and reviews differential | Differential:   - Septic abortion - Endometritis - PID/TOA - Hemorrhagic shock |
|  |  | If not -> Facilitator 1 asks learner for their differential diagnosis and reports the attending will join the learner at the bedside. |
| Learner and GYN attending (played by learner) decide on next steps | - Obtain bedside US to evaluate for retained products of conception - IV fluids - Draw blood cultures if not done yet - Order crossmatched blood if not done yet | If not -> facilitator 1 prompts learner for these components of the plan |
| Learner obtains bedside US | Facilitator on says “Your bedside US reveals ~2-3cm endometrial stripe with evidence of products of conception in left upper uterine segment.” | If not -> facilitator 1 prompts learner to obtain US |
| Learner asks for blood cultures to be drawn | ED nurse draws blood cultures | If not -> facilitator 1 prompts learner ask for blood cultures |
| Learner notes new vital signs | Vitals monitor shows HR 120, BP 90/50, T 98.7, O2 98% on 2L |  |
|  | If not -> facilitator 1 asks “Have you seen her vitals?” |  |
| Learners discuss plan each other and with ED attending | 1: Plan for immediate manual vacuum aspiration (MVA)  2: Current differential: Sepsis vs Hemorrhagic Shock | Sepsis:   - Start antibiotics - What microbes are learners worried about? (Group A Strep, Clostridium, Endotoxin related infection, vaginal flora/polymicrobial) - Broad spectrum antibiotics: vancomycin, piperacillin/tazobactam, and clindamycin   Hemorrhagic Shock:   - Remains on differential, as it is unclear how much blood the patient has lost - Transfusion may be indicated – how much and what blood products? |
|  |  | If not -> facilitator 1 prompts learners for these components of the plan |
| ED nurse hangs mock IV antibiotics |  |  |
| Learners discuss the location of MVA – should this happen in the operating room (OR) in the trauma bay | If not -> facilitator 1 prompts learners to discuss |  |
|  | The importance of this discussion is to recognize the value in *immediate* uterine evacuation, so the trauma bay is the appropriate location in our institution (ie the fastest way to perform the procedure) |  |
|  | If learner pushes for the OR -> facilitator 1 says “The OR is occupied with traumas and cannot accommodate this case for two hours.” |  |
| Learner prepares for MVA in the trauma bay | Learners recognize each preparation step:   - MVA supplies - Dilation and Evacuation kit - Uterotonics including Misoprostol, Carboprost, Methylergonovine (Methergine) - Anesthesia/sedation (fentanyl/midazolam) | If not -> facilitator 1 prompts learners to discuss each step in preparation |
| Learner consents patient | Facilitator 1 responds as patient in the consenting process, appears to be participating only partially given level of pain and worsening condition | If not -> facilitator 1 asks as patient “What is happening to me? What are you going to do to fix it?” |
| Learner performs MVA | Facilitator 1 says “The MVA is performed easily, consistent with products of conception, and you see no bleeding after the procedure.” |  |
| Learner notes new vital signs | Vitals monitor shows HR 105, BP 95/60, T 98.7, O2 99% on 2L |  |
|  | If not -> facilitator 1 asks “Have you seen her vitals?” |  |
| Learners discuss disposition plan | Learners recognize next steps:   - Consult ICU (surgical or medical ICU?) - Infectious disease consult - Additional imaging: CT abdomen to be obtained in the ED prior to transfer pending stability | If not -> facilitator 1 prompts learners to discuss disposition, including each listed step |
| END OF CASE |  |  |

**Ideal Scenario Flow**

The learners call the ED physician for report and discuss the patient’s HPI and initial exam. They ask the ED to obtain a urine pregnancy test, CBC, CMP, lactate, coagulation panel, type and screen, and venous blood gas. They then enter to visit the patient in the emergency department and obtain pertinent history including the course of the patient’s mediation abortion and how much she has bleed. They perform a bimanual and speculum exam that are notable for copious bleeding. The underpad beneath the patient continues to reveal larger and larger blood stains. Learners perform a bedside ultrasound, which reveals ~2-3cm endometrial stripe with evidence of products of conception in left upper uterine segment. Repeat vital signs show tachycardia, hypotension, and a temperature of 98.7 up from 95.3 on presentation. After examining the patient and reviewing the vitals, labs, and imaging, learners recognize the two diagnoses at the top of their differential: sepsis and hemorrhagic shock. They prepare management for both, including initiating appropriate antibiotic coverage (clindamycin and vancomycin) and considering blood transfusion. Learners recognize the importance of prompt uterine evacuation. They prepare for and perform manual vacuum aspiration in the trauma bay, which is faster than transferring the unstable patient to the operating room. After a successful uterine aspiration, the case ends when learners order a CT of the abdomen and pelvis for further source evaluation, consult the ICU for admission, and place a consult with the infectious disease team.

**Anticipated Management Mistakes**

1. Failure to recognize the importance of prompt uterine evacuation: Many of our learners felt uncomfortable with performing uterine aspiration outside of the OR in this case, however an important critical action in this simulation is to recognize the importance of prompt evacuation. We found it helpful to make the OR unavailable in the case so learners must discuss the importance of the timeframe of this procedure.
2. Uncertainty about appropriate antibiotic coverage: Our learners were uncertain of which organisms were indicated in a septic abortion and which antibiotics would have appropriate coverage. To address this, we reviewed most common organisms and appropriate antibiotic coverage explicitly in the debrief materials.
