# Supplementary material for: First-Trimester Abortion Complications: Simulation Cases for OB/GYN Residents in Sepsis and Hemorrhage
Source: MedEdPORTAL. 2020 Oct 16;16:10995. doi: 10.15766/mep_2374-8265.10995 (PMC7566226; doi:10.15766/mep_2374-8265.10995)
Supplement: Supplementary file 1 — Sepsis Simulation Case.docxHemorrhage Simulation Case.docxSimulation Images.docxPresimulation Didactic Lecture.pptxSepsis Critical Action Checklist.docxHemorrhage Critical Action Checklist.docxSepsis Debriefing Guide.docxHemorrhage Debriefing Guide.docxSepsis Postsimulation Debrief Didactic.pptxSepsis Pre-and Postsurvey.docxHemorrhage Pre-and Postsurvey.docx [file mep_2374-8265.10995-s001.zip › B. Hemorrhage Simulation Case.docx]

| **Appendix B: Hemorrhage Simulation Case Template**  **SIMULATION CASE TITLE: Assessment and management of hemorrhage at time of uterine aspiration for first-trimester abortion**  **AUTHORS: Armide Storey, BS, Katharine White MD, MPH, Kelly Treder, MD, MPH, Elisabeth Woodhams, MD, MSc, Shannon Bell, MD, Rachel Cannon, MD, MSc**  **LEARNER AUDIENCE: OB/GYN Residents** | |
| --- | --- |
| **PATIENT NAME: Stephanie**  **PATIENT AGE: 25 years**  **CHIEF COMPLAINT: Hemorrhage at time of uterine aspiration for first-trimester abortion**  **PHYSICAL SETTING: Ambulatory gynecologic procedure unit** | |
|  | |
| **Brief narrative description of case** | The patient is a 24yo G4P3003 who hemorrhages during a first-trimester aspiration abortion at 8w5d. The simulation provides opportunities for learners to practice identification and management of dangerous bleeding at the time of aspiration abortion. |
| **Primary Learning Objectives** | By the end of this activity, learners will be able to:   1. Demonstrate improved knowledge of the most common etiologies of hemorrhage at the time of aspiration abortion 2. Develop a plan to evaluate and manage the most common etiologies of hemorrhage as a first-trimester abortion complication 3. Demonstrate effective communication skills and workflow management with co-residents and colleagues from different disciplines in evaluating an emergency scenario and transferring a patient to an escalated level of care |
| **Critical Actions** | 1. Correctly identify dangerous bleeding 2. Transition from manual to electric suction for uterine evacuation in the setting of ongoing bleeding 3. Evaluate uterine bleeding using ultrasound 4. Evaluate the most common etiologies of hemorrhage    1. Atony (Tone)    2. Retained products of conception (Tissue)    3. Cervical laceration (Trauma)    4. Uterine perforation (Trauma)    5. Coagulopathy (Thrombin) 5. Perform uterine massage 6. Administer uterotonics, recognizing contraindications    1. Methylergonovine contraindicated in patients with hypertension    2. Carboprost contraindicated in patients with asthma 7. Perform cervical evaluation to evaluate for laceration    1. ask for additional instruments for proper evaluation of the cervix 8. Recognize worsening vital signs: tachycardia, hypotension 9. Place Foley catheter for tamponade 10. Recognize need for escalation of care and transfer to operating room (OR) 11. Communicate with anesthesia to draw and order labs (CBC, PTT, INR, Fibrinogen, Type and Cross) 12. Communicate with staff to draw and order labs (CBC, PTT, INR, Fibrinogen, Type and Cross) 13. Call OR staff to coordinate transfer to OR 14. Call fellow residents to coordinate transfer to OR and any attending level backup required 15. Transfer patient to the OR     1. Transfer patient to stretcher     2. Connect patient to supplemental oxygen and cardiac monitoring     3. Bring stretcher to OR 16. Demonstrate effective communication skills and workflow management with co-residents, anesthesia, nursing, medical assistant, and operating room colleagues |
| **Learner Preparation or Prework** | Learners participate in a two-hour case-based didactic lecture prior to the simulation with an emphasis on institutional practices, preoperative evaluation and preparation, and potential challenges and complications (Appendix D).  Prior to entering the simulation, learners are told “Your patient is a 24yo G4P3003 with a history of three prior uncomplicated vaginal deliveries and asthma well controlled with albuterol PRN. She is presenting to the gynecologic procedure unit for a first-trimester aspiration abortion at 8w5d.” |
| **Equipment** | Simulation Mannequin  “Blood” stained underpads (Chux)  Simulated IV fluids  Simulated bedside ultrasound  Dilation and Evacuation kit  Manual Vacuum Aspiration with varying canula sizes  Patient chart with history and orders forms  Vitals monitor |

| Initial Presentation | | | |
| --- | --- | --- | --- |
| **Initial vital signs** | BP: 120/80 HR: 80 RR: 16 SpO2: 99 Temp: 97 | | |
| **Overall Setting and Appearance** | Learners enter the ambulatory gynecologic procedure unit and see an obstetric mannequin seated in a gynecologic procedure chair. She is dressed in a patient gown with IV in place and IV fluids hanging. | | |
| **Confederates (e.g., standardized participants) and their roles in the room at case start** | Registered nurse (RN) confederate: Staff RN playing regular support role  Medical assistant (MA) confederate: Staff MA playing regular support role  Operating room (OR) confederate: OR staff playing regular support role  Anesthesiologist confederate: Anesthesiology MD playing regular role  Facilitator 1: Attending level physician. Adjusts vital signs, reports patient status, and provided labs/images as the case progresses  Facilitator 2: Observes, replaces underpads to simulate ongoing bleeding | | |
| **Learners Role** | Family Planning attending  Resident  Medical Student  On Call Resident  Patient’s escort  Observers | | |
| **HPI** | HPI is given to learners by facilitators at the start of the case.  Patient is a 24yo G4P3003 woman presenting to the gynecologic procedure unit for a first-trimester aspiration abortion at 8w5d. | | |
| **Past Medical/ Surgical/Obstetric History** | **Medications** | **Allergies** | **Family History** |
| Asthma  History of 3 prior uncomplicated vaginal deliveries 9, 7, and 3 years ago. | Albuterol PRN | NKDA | No applicable family history |
| **Physical Examination** | | | |
| **General** | Appears mildly anxious, otherwise well | | |
| **GU** | External genitalia without erythema, exudate or discharge. Vaginal vault is without discharge. Cervix is of normal color without lesions. The external os is closed. There is no bleeding noted. Uterus is noted to be approximately 8wk in size, anteverted, and nontender. | | |

| Instructor Notes - Changes and CASE Branch Points | | |
| --- | --- | --- |
| **Intervention / Time point** | **Change in Case** | **Additional Information** |
| Learner enters room, performs universal protocol | Anesthesiologist administers Propofol for sedation |  |
| Learner performs manual vacuum aspiration (MVA) for uterine evacuation | Facilitator 1 alerts learners: “The MVA quickly fills with blood” |  |
| Learner removes MVA curette, discharges, and repeats MVA | Facilitator 1 alerts learners: “The MVA quickly fills with blood” | Facilitator 2 replaces underpad with pad stained with scant blood |
| Learner removes MVA curette, discharges, and repeats MVA | Facilitator 1 alerts learners: “The MVA quickly fills with blood” | Facilitator 2 replaces underpad with pad stained with mild blood |
| Learner asks for ultrasound (US) to evaluate bleeding | Facilitator 1 alerts learner “US shows hematoma in uterus” |  |
|  | If not -> nurse says “We have the US available if you want it” |  |
|  | If still no -> facilitator 1 says “You should evaluate the uterine bleeding using US” |  |
| Learner asks for electric suction and performs electric suction aspiration (EVA) under US guidance | Facilitator 1 alerts learners “US reveals rapid reaccumulation of  blood and no free fluid in  the cul-de-sac” | Facilitator 2 replaces underpad with pad stained with more blood |
|  | If not -> facilitator 1 asks “Your MVA is not evacuating blood quickly enough. What else do you have available to you?” |  |
|  | If still no -> facilitator 1 says “You should transition to the EVA to more efficiently evacuate the blood” |  |
| Learner removes EVA curette | Facilitator 1 alters learners “The US shows rapid reaccumulation of blood” | Facilitator 2 replaces underpad with pad stained with more blood |
| Learner reviews differential for hemorrhage | If not -> facilitator 1 asks “What is your differential?” | Answer:  Tone- atony  Tissue- retained products of conception  Trauma- cervical laceration and uterine perforation  Thrombin - coagulopathy |
| Learner performs uterine massage | Facilitator 1 alerts learner “You’ve noticed atony, which shows some initial improvement after massage” | Facilitator 2 replaces underpad with pad stained with more blood |
|  | If not -> nurse asks “Have you tried uterine massage?” |  |
| Learner asks for utero-tonics which are administered by nursing | Uterotonics available:  - Methylergonovine (Methergine) 200mcg IM  - Misoprostol 600 mcg  - Carboprost 250 mcg IM  - Pitocin |  |
|  | If not -> nurse asks “Do you want me to get any medications for you?” |  |
| Learner reviews uterotonic contraindications | If not -> facilitator 1 asks “what are contraindications to uterotonics?” | Answer:  - Hypertension for methylergonovine  - Asthma for carboprost |
| Learner administers uterotonics, avoiding carboprost given patient’s asthma history | Facilitator 1 alerts learners: “You see continued bleeding.” | Facilitator 2 replaces underpad with pad stained with more blood |
| Learner performs cervical evaluation | Facilitator 1 alerts learners: “Your cervical exam reveals ongoing bleeding. You can visualize the anterior lip of cervix but are unable to clearly visualize posterior lip. You can palpate an irregular posterior lip with no clear defect, but your exam is limited by ongoing bleeding.” |  |
|  | If not -> facilitator 1 asks “Have you evaluated the cervix for laceration?” |  |
| Learner asks for cervical laceration kit, which at our institution is available only on the Labor & Delivery floor (this is for learners to have on hand in case a cervical laceration becomes evident, as it takes time to retrieve this kit in our institution) | If not -> facilitator 1 asks “How will you manage this patient if there is a cervical laceration?” | Facilitator 2 replaces underpad with pad stained with more blood |
| Learner repeats bimanual exam | Facilitator 1 alerts learners: “Your bimanual exam demonstrates a boggy, soft, enlarged uterus.” |  |
|  | If not -> facilitator 1 asks “What has the response been to the uterotonics you administered?” |  |
| Learner asks for repeat set of vital signs | HR 105, BP 90/50 |  |
|  | If not -> RN asks “Would you like me to get a set of vital signs?” |  |
| Learner asks for and places foley balloon under US guidance | Facilitator 1 alerts learners: “You see ongoing bleeding around the balloon” | Facilitator 2 replaces underpad with pad stained with more blood |
|  | If not -> facilitator 1 asks “What is another way you can achieve hemostasis?” |  |
|  | If still no -> facilitator 1 says “You should consider Foley catheter tamponade.” |  |
| Learners articulate that uterine atony is their leading differential diagnosis | If not -> facilitator 1 asks “Given the tone on your exam, what is your most likely diagnosis in this case?” |  |
| Learners discuss plan to transfer patient to the operating room (OR) for further management vs IR for uterine artery embolization | If not -> facilitator 1 asks “Is it time to escalate care for this patient? What options do you have” |  |
| Learners decide to transfer to the OR for further visualization and surgical management given their primary concern for atony. | If not -> facilitator 1 says “Given that atony is so high on your differential, plan to transfer to the OR for evaluation and possible surgical management is best. Should you discover a cervical laceration with better visualization, you could consider repair in the OR vs. IR embolization then.” |  |
| Learners ask for IV fluids to be hung | RN hangs IV fluids |  |
|  | If not -> facilitator 1 asks “How will you keep her blood pressure up during transfer to the OR?” |  |
| Learners asks anesthesiologist to order and draw (CBC, PTT, INR, Fibrinogen, Type and Cross) to be drawn in preparation for OR transfer | Anesthesiologist draws labs |  |
|  | If not -> anesthesiologist asks “What labs do you need to draw in preparation for transfer to the OR?” |  |
| Learners request for blood to be sent from the bank to the OR. | If not -> facilitator 1 asks “Do you want to order blood from the bank?” |  |
| Learner calls OR colleagues and other residents on service to prepare for OR transfer | If not -> facilitator 1 asks “Who do you need to alert for transfer to the OR?” |  |
| Learners transfers patient to hospital stretcher, connects her to oxygen and cardiac monitoring, and brings the bed to the doors of the OR |  |  |
| END OF CASE |  |  |

**Ideal Scenario Flow**

The learners enter the room and find the patient ready for her procedure. After learners perform universal protocol, anesthesia administers Propofol for sedation. They begin a manual vacuum aspiration (MVA), which fills quickly with blood. After a few passes with the MVA, ultrasound (US) evaluation reveals hematometra. Learners transition to electric vacuum aspiration (EVA) for further uterine evacuation and US shows rapid reaccumulation of fluid in the uterus without free fluid in the cul-de-sac. The underpad beneath the patient continues to reveal larger and larger blood stains. Learners review the differential for hemorrhage and perform uterine massage, recognizing atony with mild improvement. They administer uterotonic medications, avoiding carboprost because of the patient’s history of asthma. The learner communicates with the anesthesia and nursing staff to draw and order labs (CBC, PTT, INR, Fibrinogen). A cervical evaluation is limited by ongoing bleeding, but reveals an irregular posterior lip with no clear defect on palpation. Learners request cervical laceration kit which needs to be retrieved from another area of the hospital. Repeat vitals show tachycardia to 105 and hypotension to 90/50. Foley catheter balloon tamponade does not achieve hemostasis, and learners decide to bring the patient to the operating room (OR). Learners coordinate with OR staff, draw appropriate labs, hang IV fluids, and transfer the patient to a hospital stretcher, connect her to oxygen and cardiac monitoring, and the case ends when the patient is brought to the doors of the OR.

**Anticipated Management Mistakes**

1. Failure to switch to electric suction from manual vacuum aspiration: We found that learners do not always immediately recognize when uterine bleeding is too brisk for manual vacuum aspiration (MVA) and appropriately transition to electric vacuum aspiration. We found it helpful to prompt learners during the case if they fail to transition after discharging the MVA three times.
2. Failure to perform a cervical exam: Some of our learners did not recognize the need for a cervical exam to evaluate for possible laceration, leading to the potential of a delayed diagnosis of laceration. In our institution where cervical laceration kits are only available in a different part of the hospital from where abortions take place, it is important to promptly recognize the possibility of laceration. We found it helpful to prompt learners after reviewing the differential diagnosis.
3. Uncertainty about escalation to the operating room: Many of our learners were unfamiliar with the procedure of transferring a patient to the operating room (OR) from the ambulatory procedure unit. To address this, we reviewed the steps involved, including drawing labs, hanging fluids, contacting OR staff and fellow residents, and physically bringing the patient to the OR.
