# Supplementary material for: First-Trimester Abortion Complications: Simulation Cases for OB/GYN Residents in Sepsis and Hemorrhage
Source: MedEdPORTAL. 2020 Oct 16;16:10995. doi: 10.15766/mep_2374-8265.10995 (PMC7566226; doi:10.15766/mep_2374-8265.10995)
Supplement: Supplementary file 1 — Sepsis Simulation Case.docxHemorrhage Simulation Case.docxSimulation Images.docxPresimulation Didactic Lecture.pptxSepsis Critical Action Checklist.docxHemorrhage Critical Action Checklist.docxSepsis Debriefing Guide.docxHemorrhage Debriefing Guide.docxSepsis Postsimulation Debrief Didactic.pptxSepsis Pre-and Postsurvey.docxHemorrhage Pre-and Postsurvey.docx [file mep_2374-8265.10995-s001.zip › C. Simulation Images.docx]

**Appendix C: Simulation Images**

**Simulated Emergency Department**

**(The Solomont Center for Clinical Simulation and Nursing Education at Boston Medical Center)**

Author owned.

**
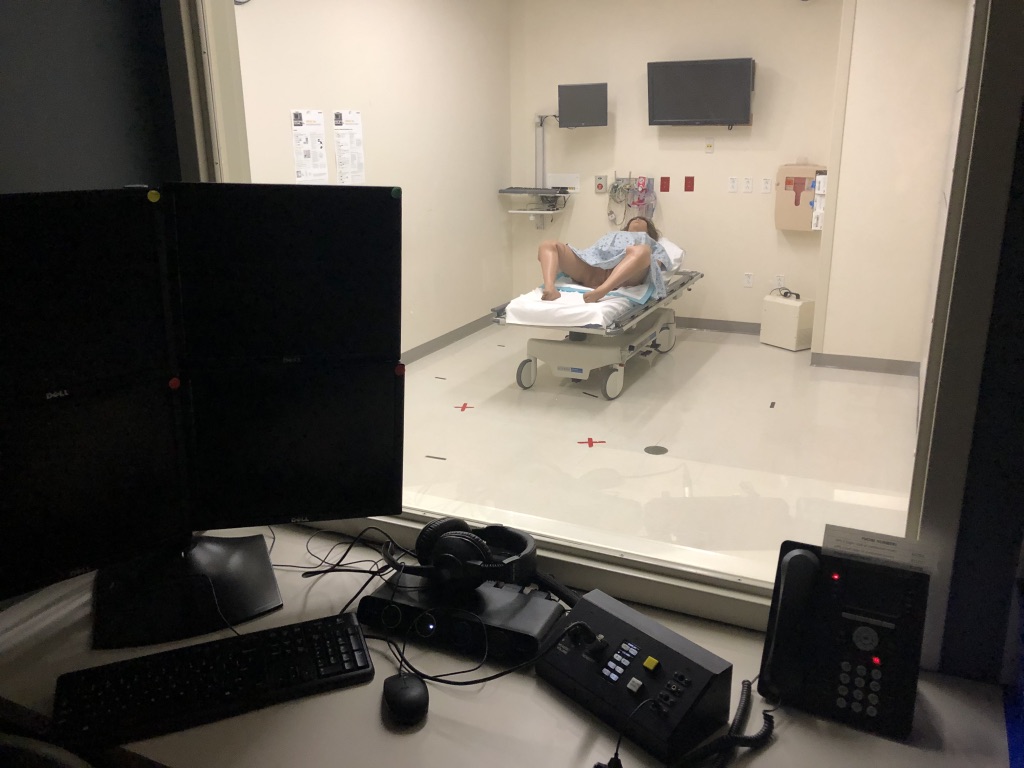
**

**Simulated Emergency Department as viewed through one-way glass by facilitators**

Author owned.

**
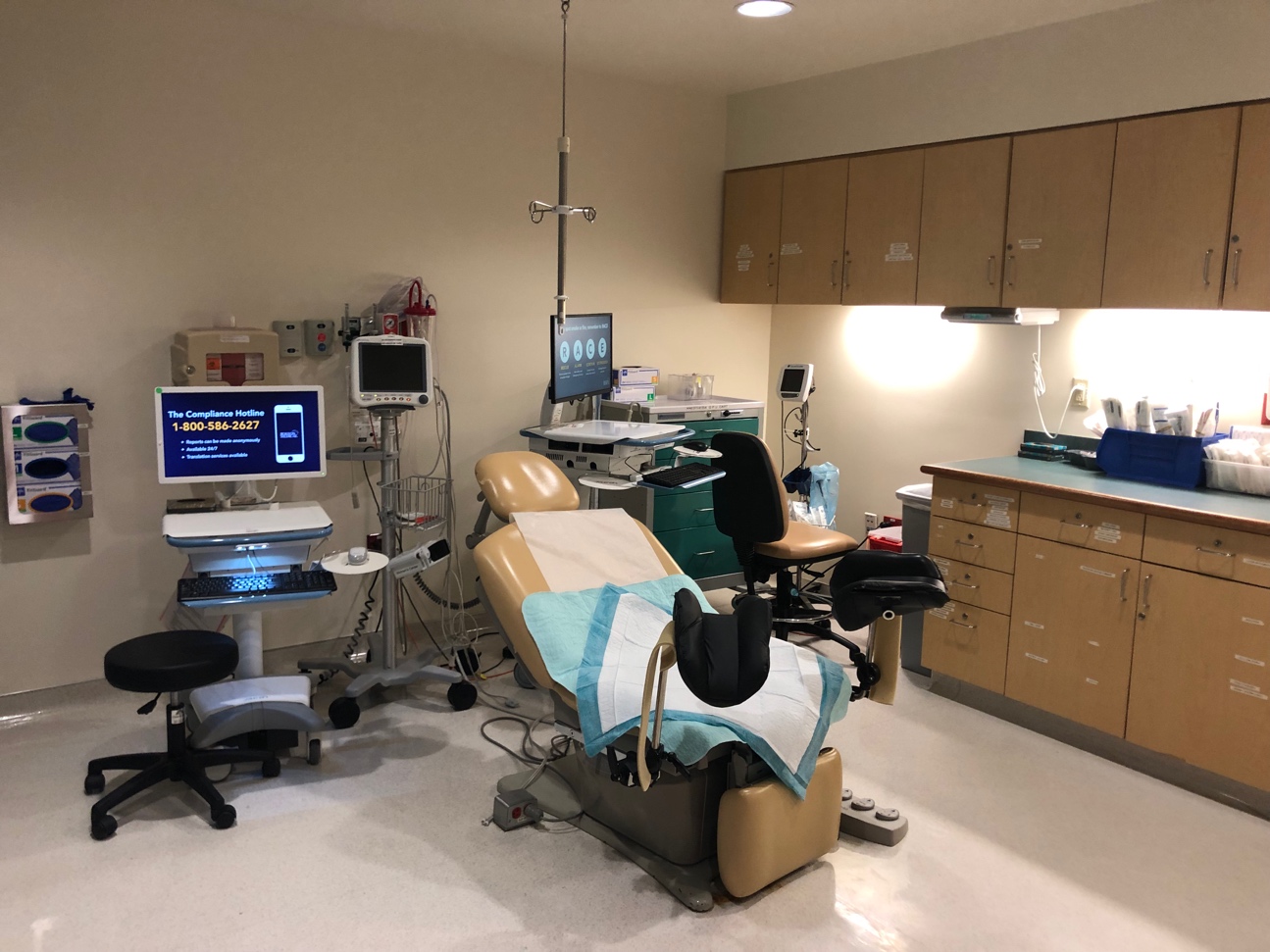
**

**Gynecologic Procedure Unit**

Author owned.


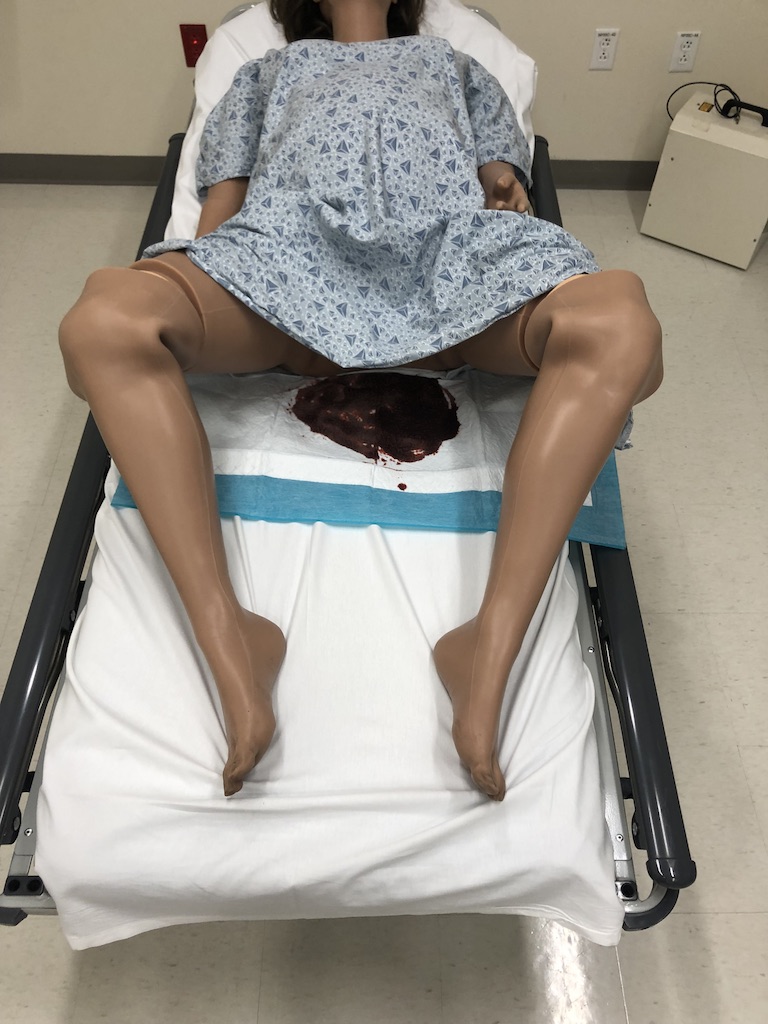


**“Blood” covered underpad (chuck) under simulation model.**

**Simulated blood was purchased from a Halloween store.**

Author owned.
