# Supplementary material for: First-Trimester Abortion Complications: Simulation Cases for OB/GYN Residents in Sepsis and Hemorrhage
Source: MedEdPORTAL. 2020 Oct 16;16:10995. doi: 10.15766/mep_2374-8265.10995 (PMC7566226; doi:10.15766/mep_2374-8265.10995)
Supplement: Supplementary file 1 — Sepsis Simulation Case.docxHemorrhage Simulation Case.docxSimulation Images.docxPresimulation Didactic Lecture.pptxSepsis Critical Action Checklist.docxHemorrhage Critical Action Checklist.docxSepsis Debriefing Guide.docxHemorrhage Debriefing Guide.docxSepsis Postsimulation Debrief Didactic.pptxSepsis Pre-and Postsurvey.docxHemorrhage Pre-and Postsurvey.docx [file mep_2374-8265.10995-s001.zip › D. Presimulation Didactic Lecture.pptx]

## Slide 1
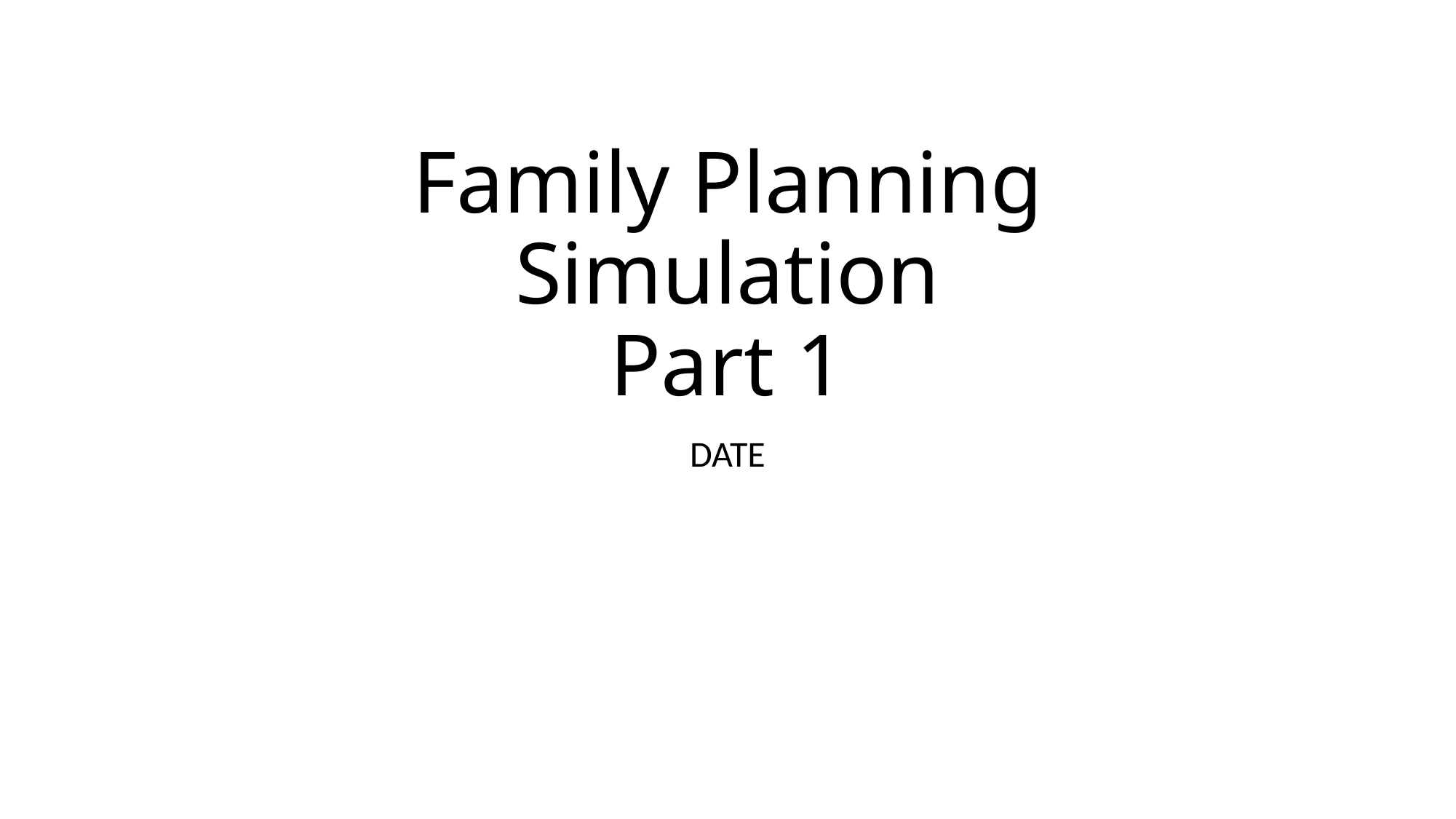

# Family Planning Simulation Part 1
DATE

## Slide 2
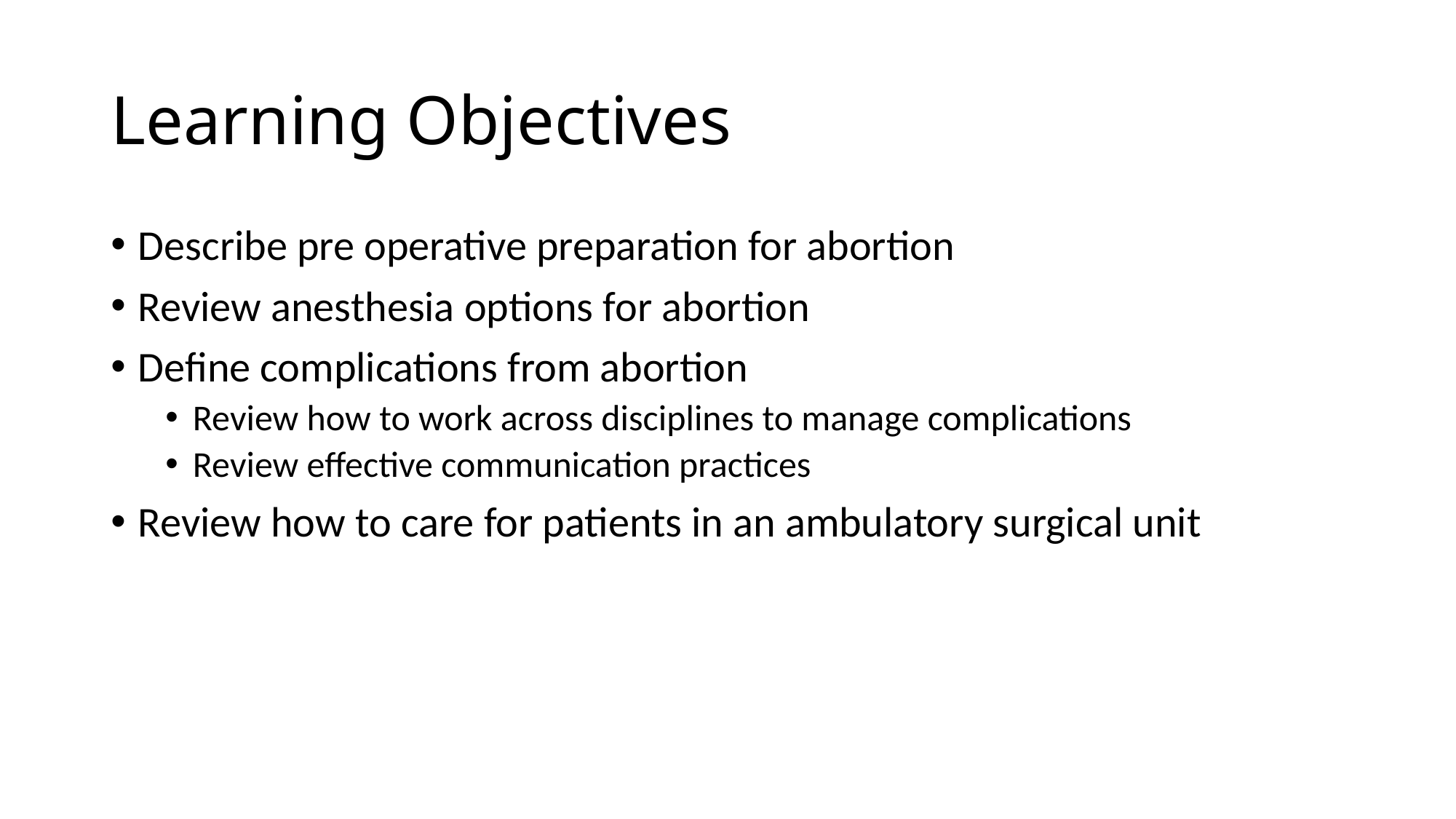

# Learning Objectives
Describe pre operative preparation for abortion
Review anesthesia options for abortion
Define complications from abortion
Review how to work across disciplines to manage complications
Review effective communication practices
Review how to care for patients in an ambulatory surgical unit

## Slide 3
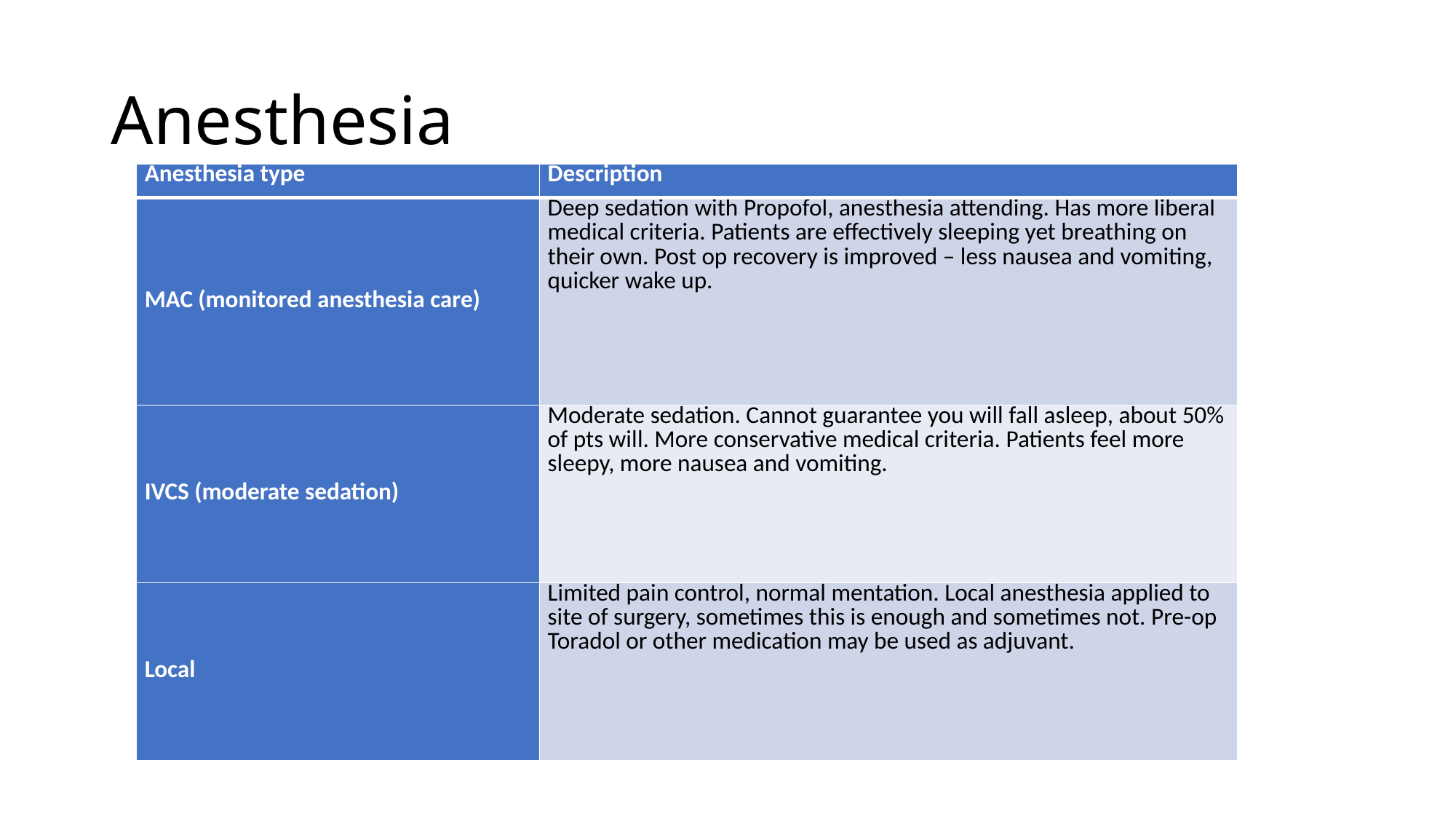

# Anesthesia
| Anesthesia type | Description |
| --- | --- |
| MAC (monitored anesthesia care) | Deep sedation with Propofol, anesthesia attending. Has more liberal medical criteria. Patients are effectively sleeping yet breathing on their own. Post op recovery is improved – less nausea and vomiting, quicker wake up. |
| IVCS (moderate sedation) | Moderate sedation. Cannot guarantee you will fall asleep, about 50% of pts will. More conservative medical criteria. Patients feel more sleepy, more nausea and vomiting. |
| Local | Limited pain control, normal mentation. Local anesthesia applied to site of surgery, sometimes this is enough and sometimes not. Pre-op Toradol or other medication may be used as adjuvant. |

## Slide 4
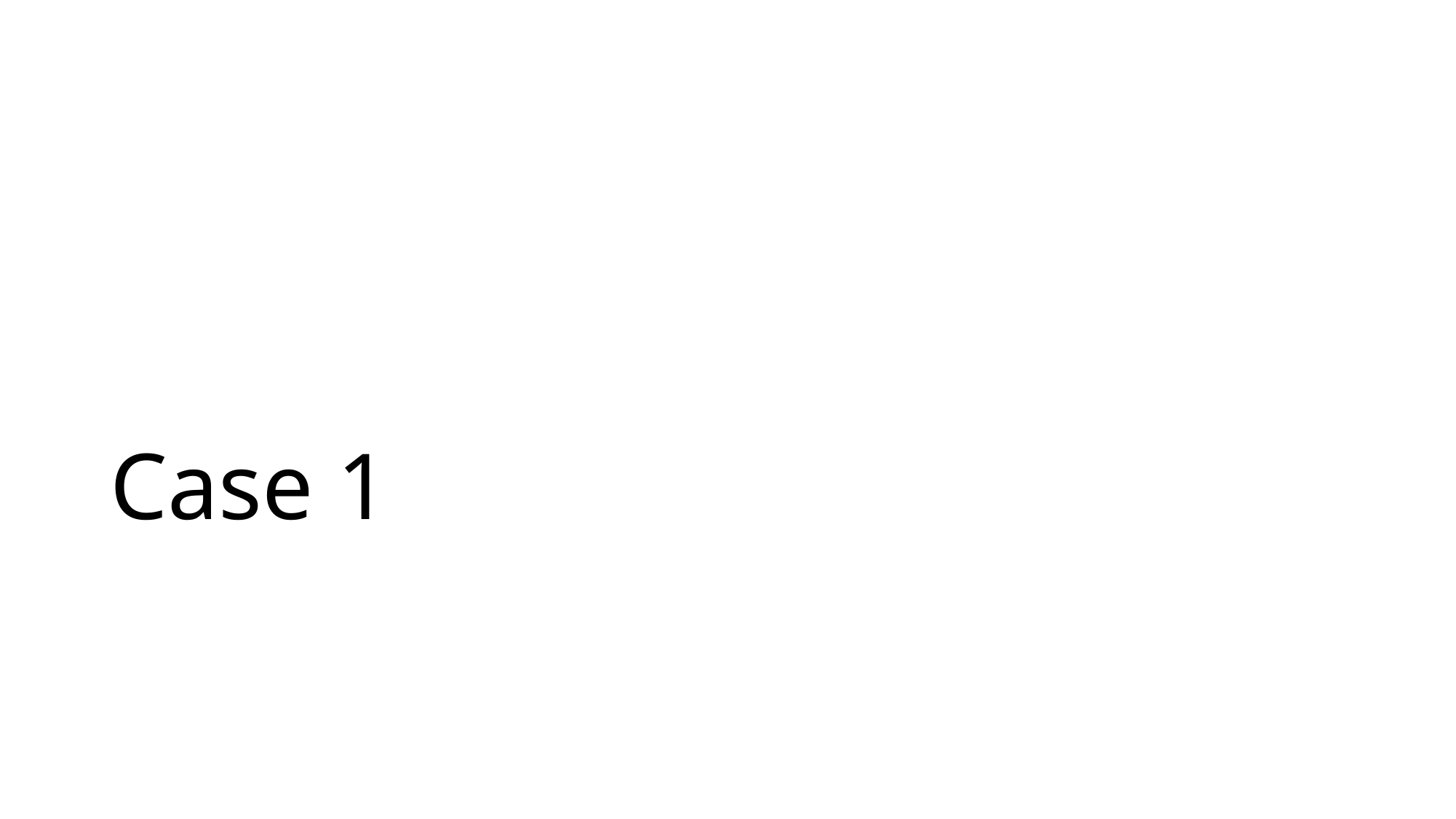

# Case 1

## Slide 5
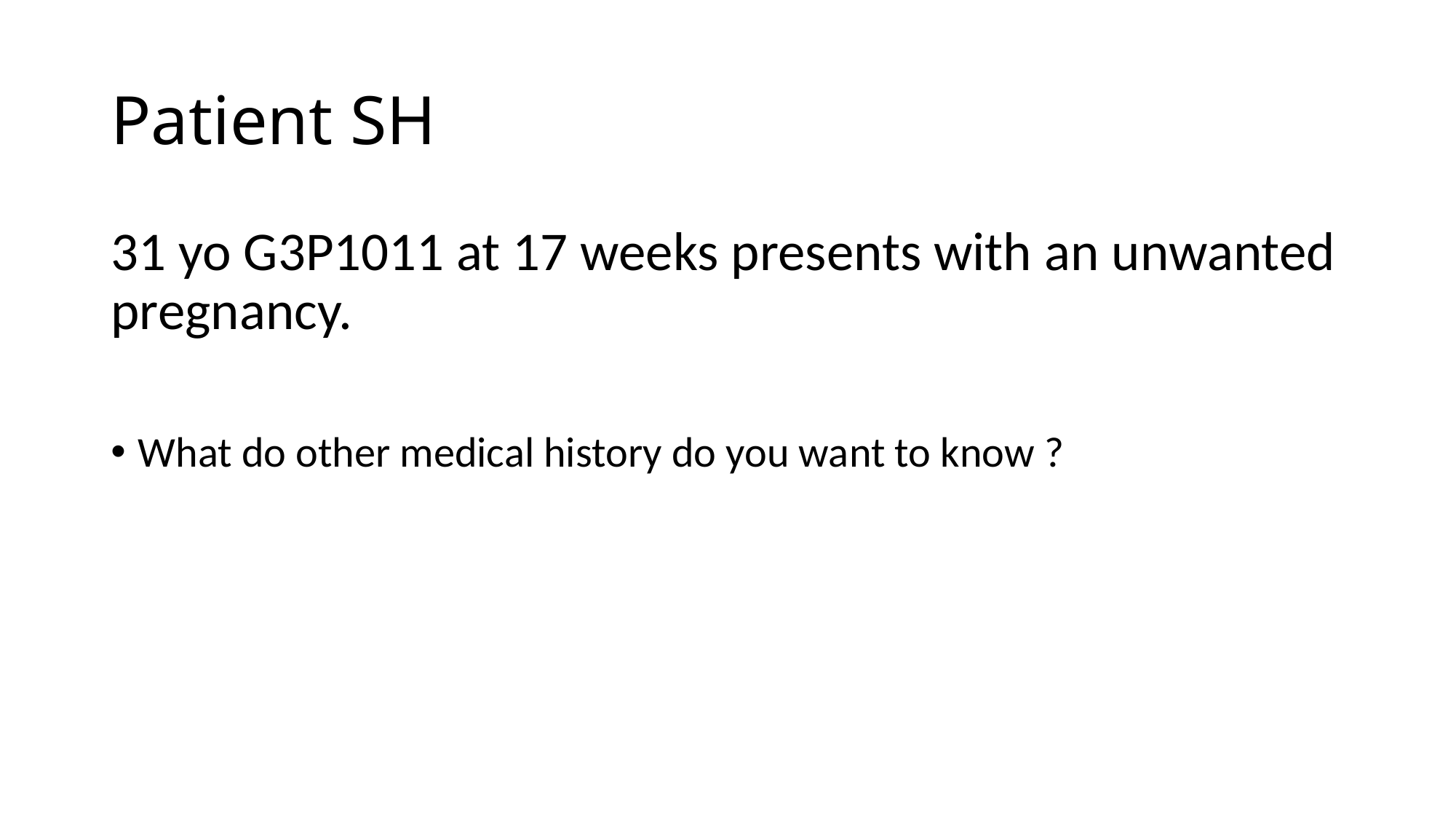

# Patient SH
31 yo G3P1011 at 17 weeks presents with an unwanted pregnancy.
What do other medical history do you want to know ?

## Slide 6
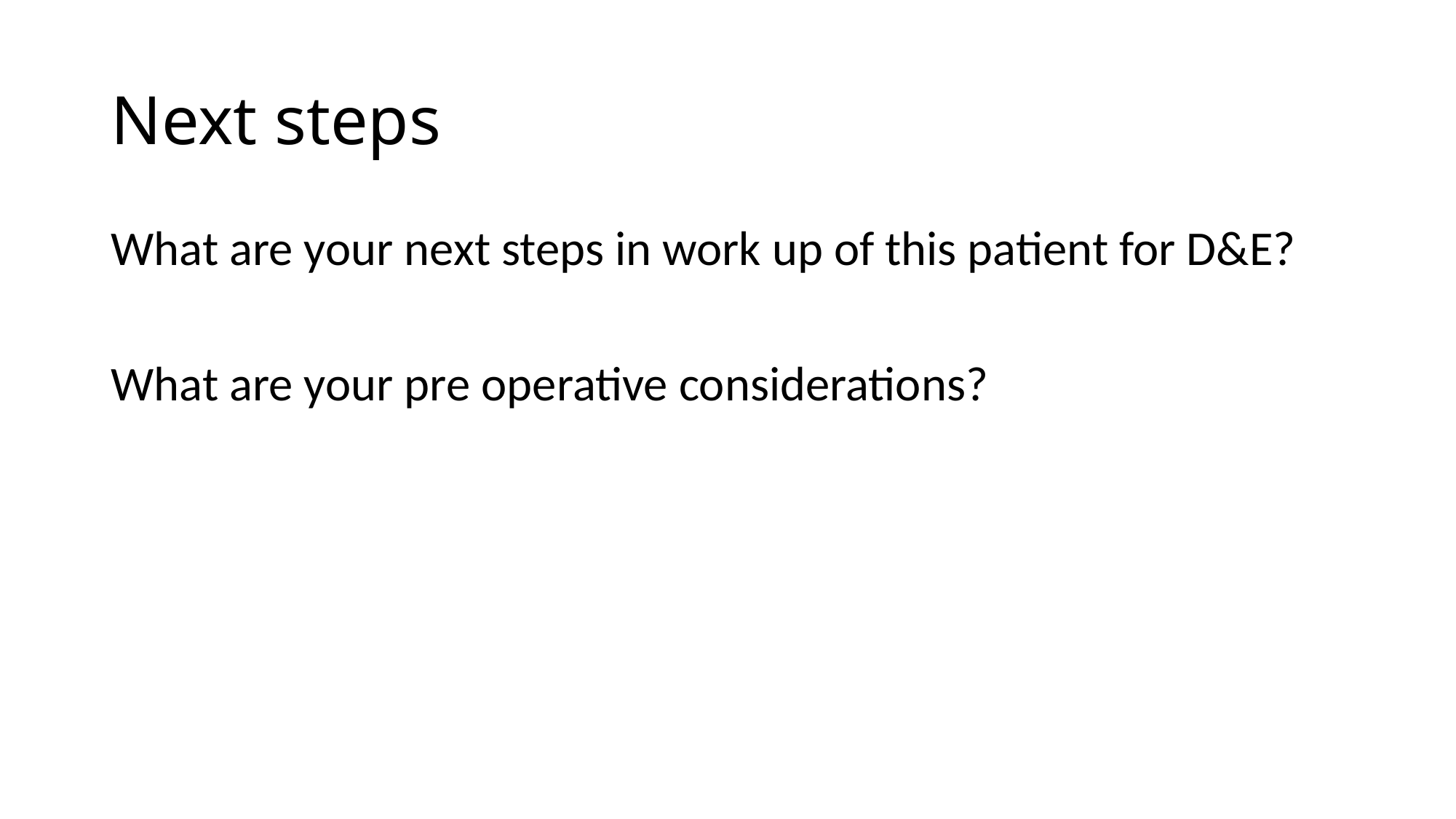

# Next steps
What are your next steps in work up of this patient for D&E?
What are your pre operative considerations?

## Slide 7
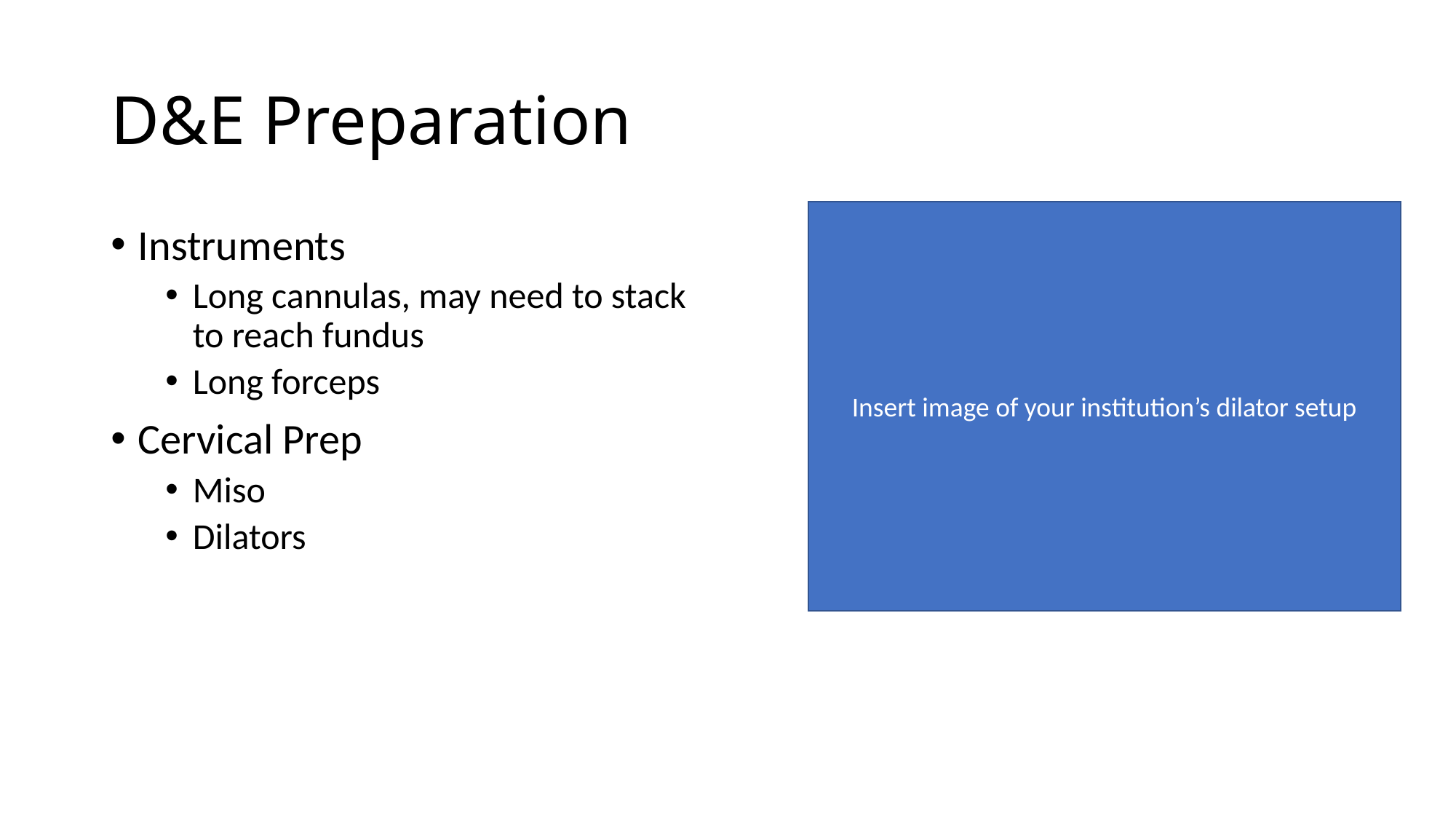

# D&E Preparation
Insert image of your institution’s dilator setup
Instruments
Long cannulas, may need to stack to reach fundus
Long forceps
Cervical Prep
Miso
Dilators

## Slide 8
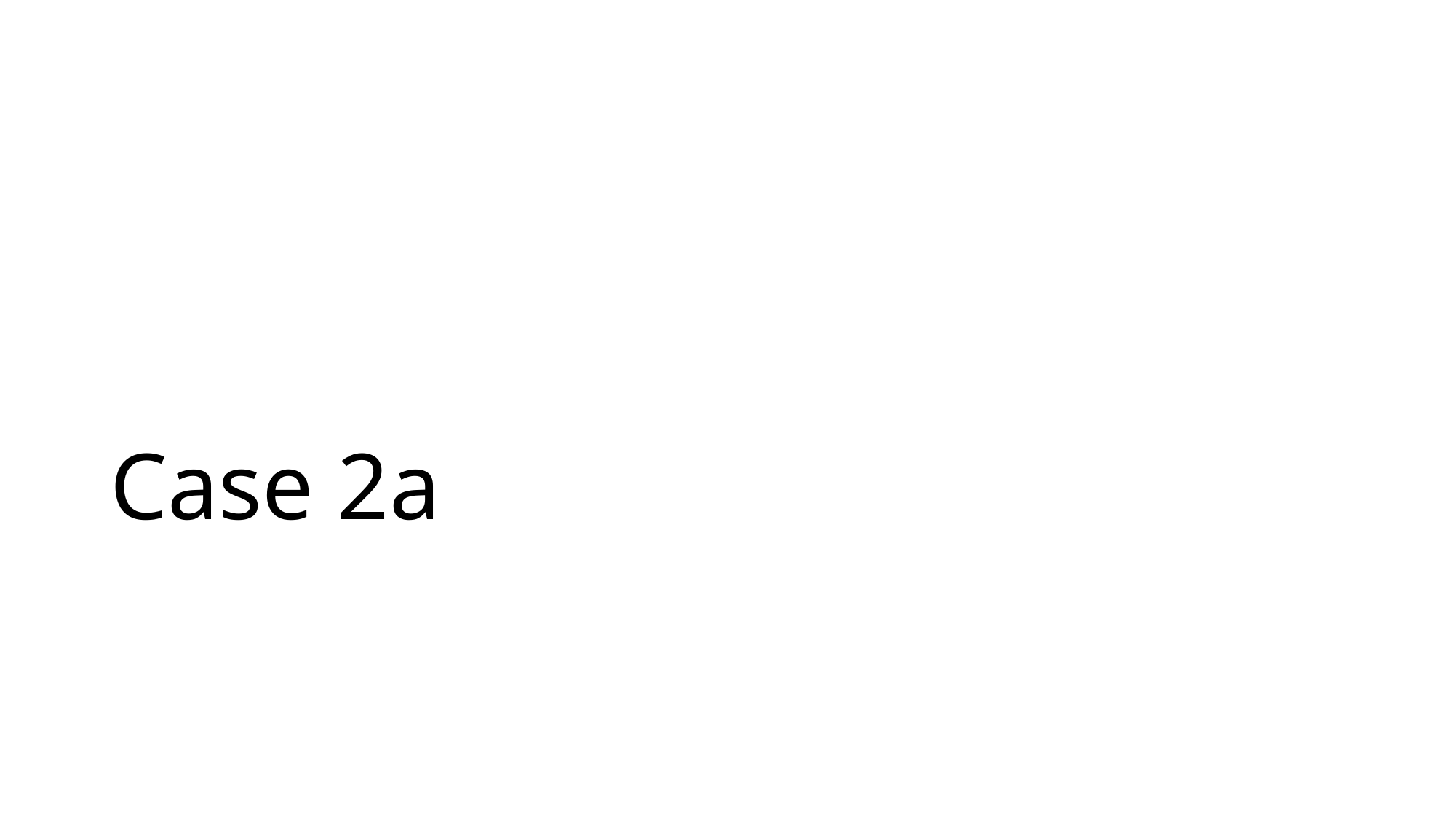

# Case 2a

## Slide 9
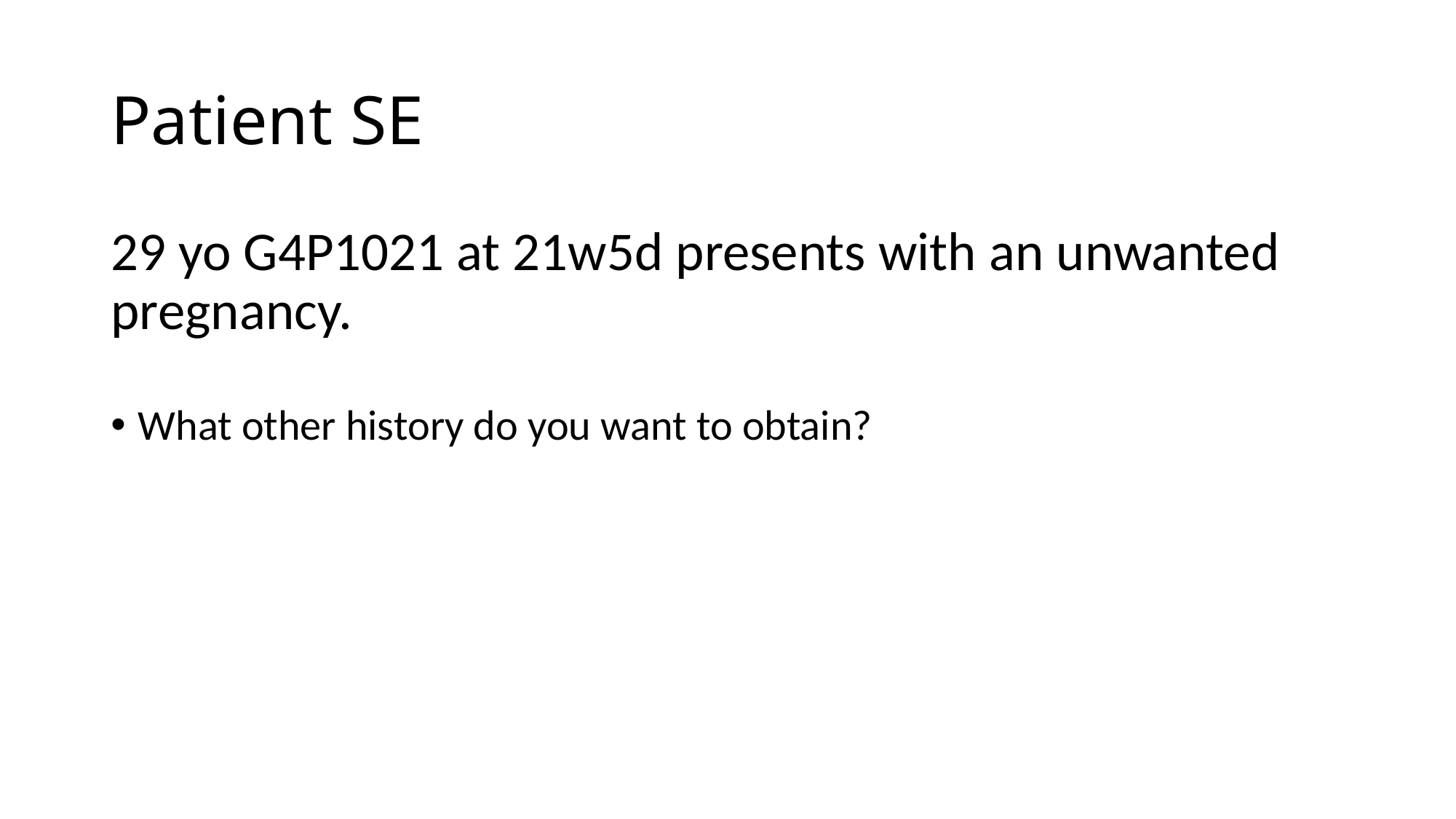

# Patient SE
29 yo G4P1021 at 21w5d presents with an unwanted pregnancy.
What other history do you want to obtain?

## Slide 10
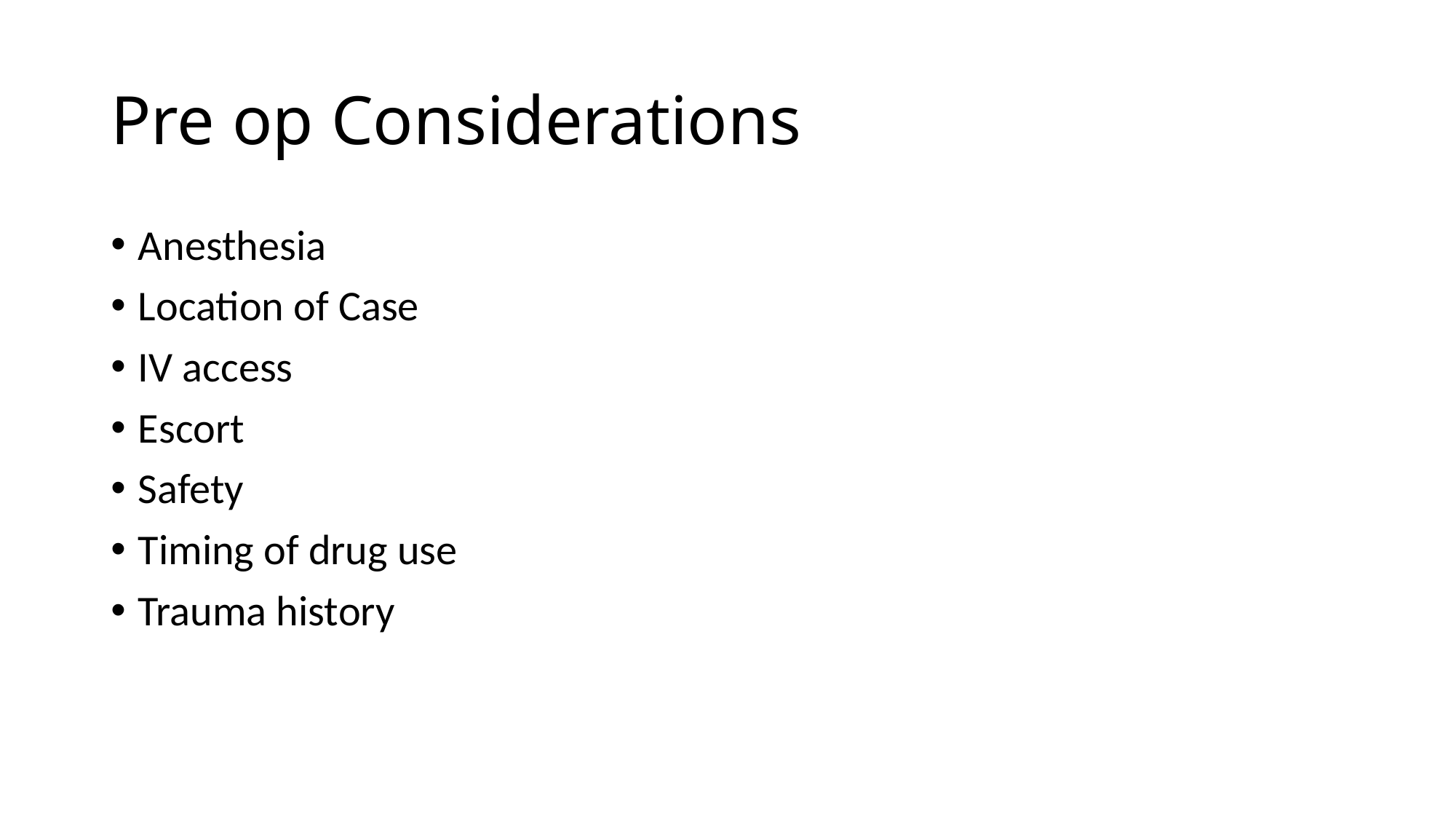

# Pre op Considerations
Anesthesia
Location of Case
IV access
Escort
Safety
Timing of drug use
Trauma history

## Slide 11
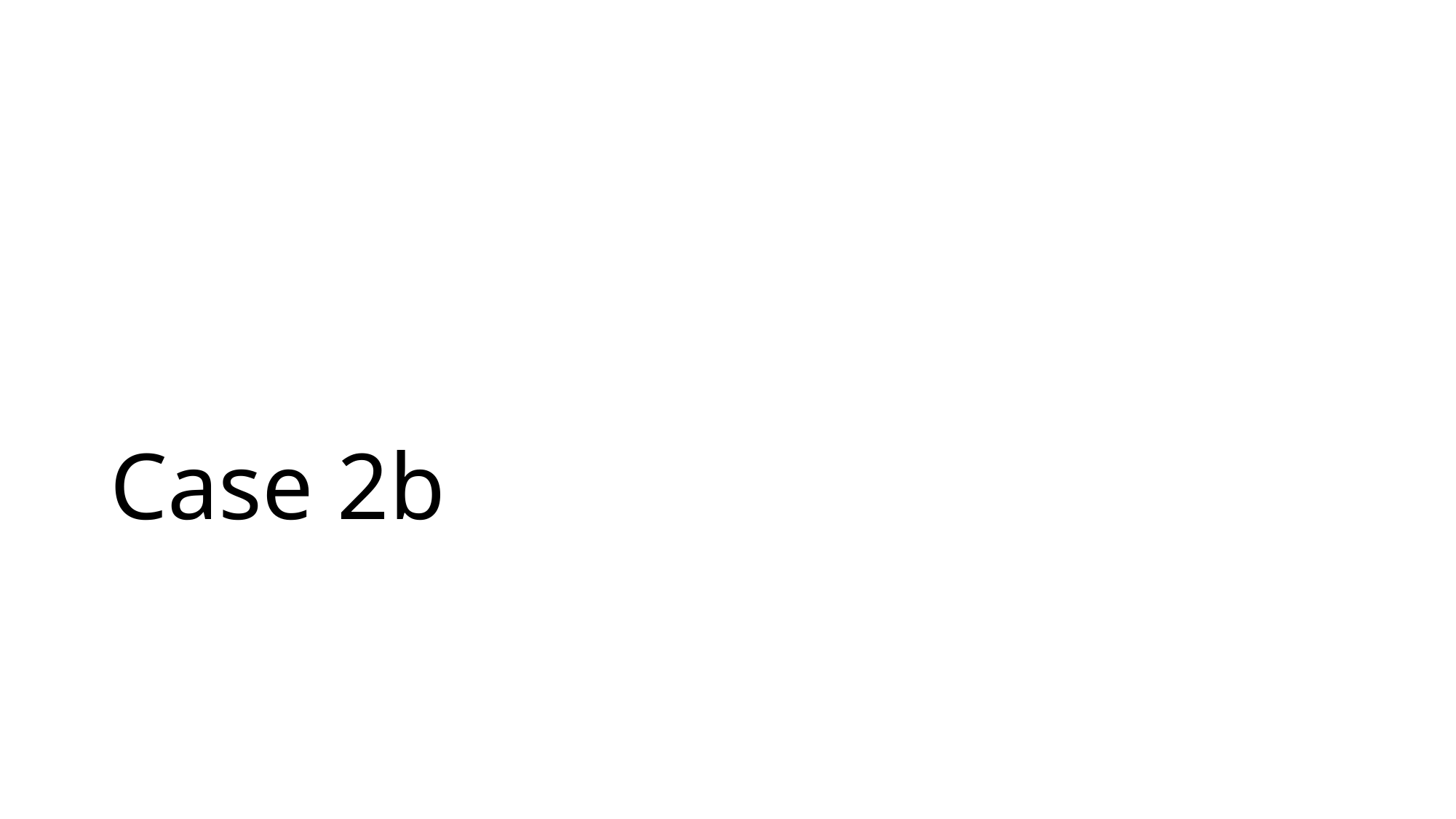

# Case 2b

## Slide 12
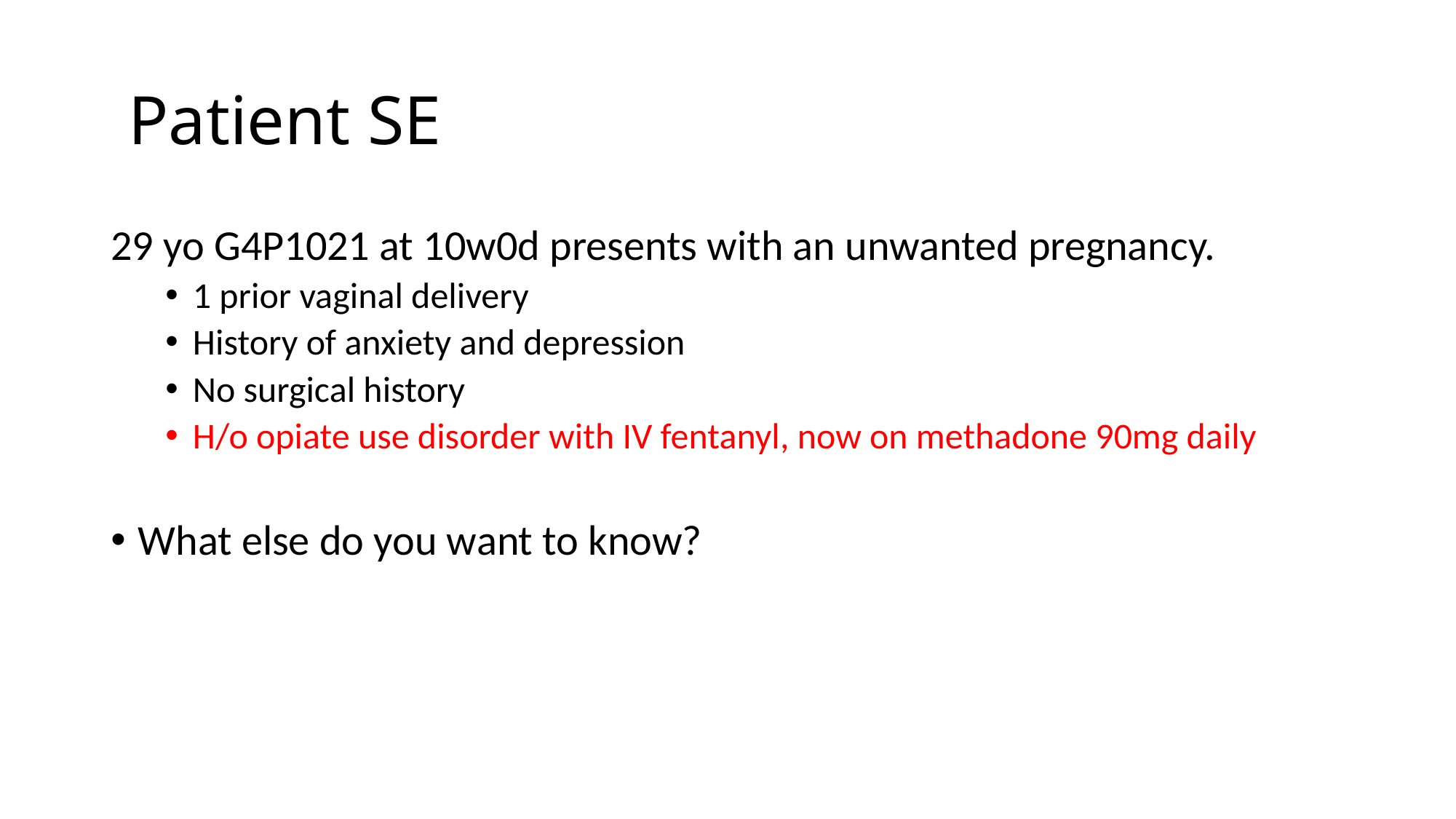

# Patient SE
29 yo G4P1021 at 10w0d presents with an unwanted pregnancy.
1 prior vaginal delivery
History of anxiety and depression
No surgical history
H/o opiate use disorder with IV fentanyl, now on methadone 90mg daily
What else do you want to know?

## Slide 13
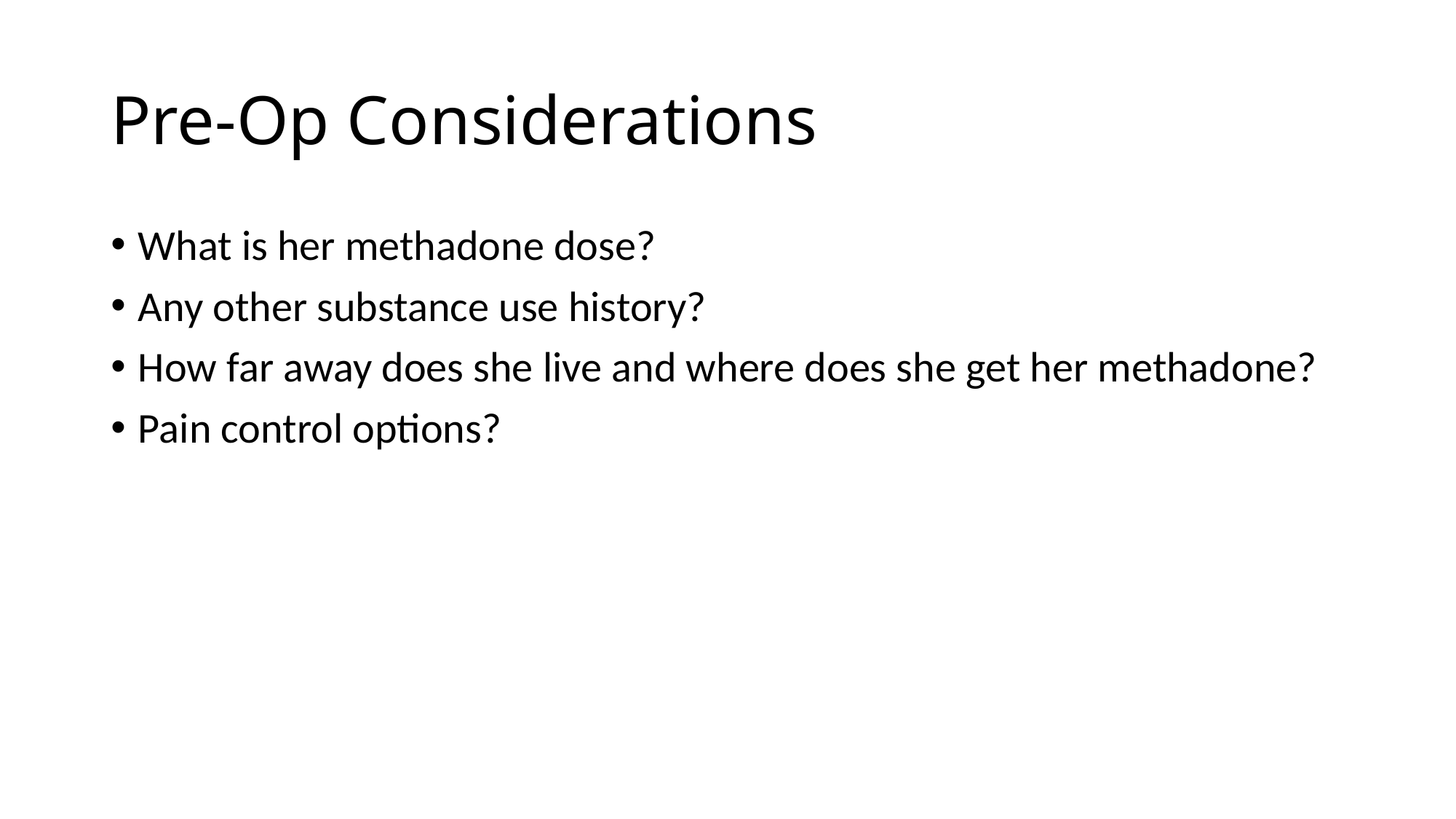

# Pre-Op Considerations
What is her methadone dose?
Any other substance use history?
How far away does she live and where does she get her methadone?
Pain control options?

## Slide 14
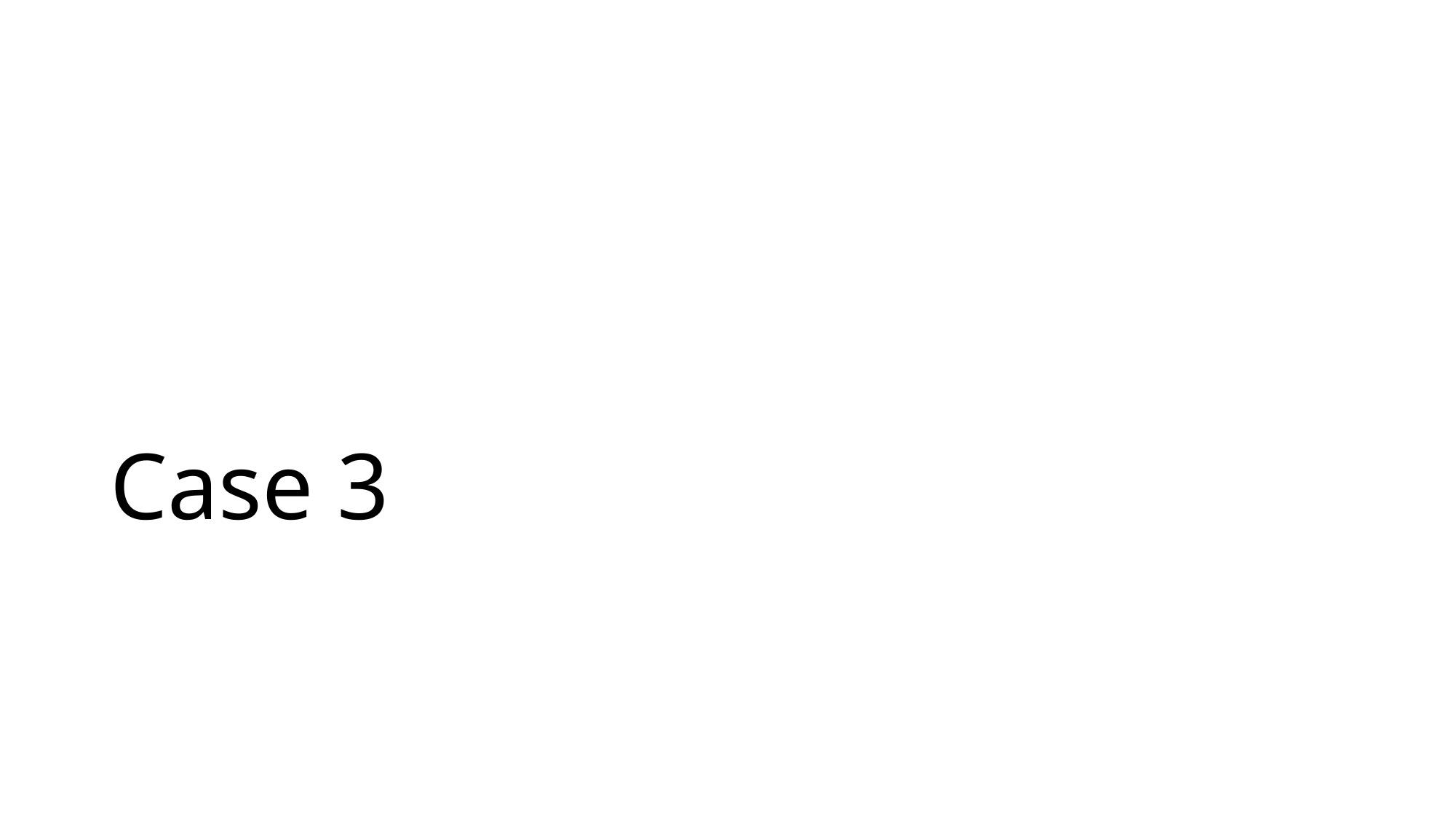

# Case 3

## Slide 15
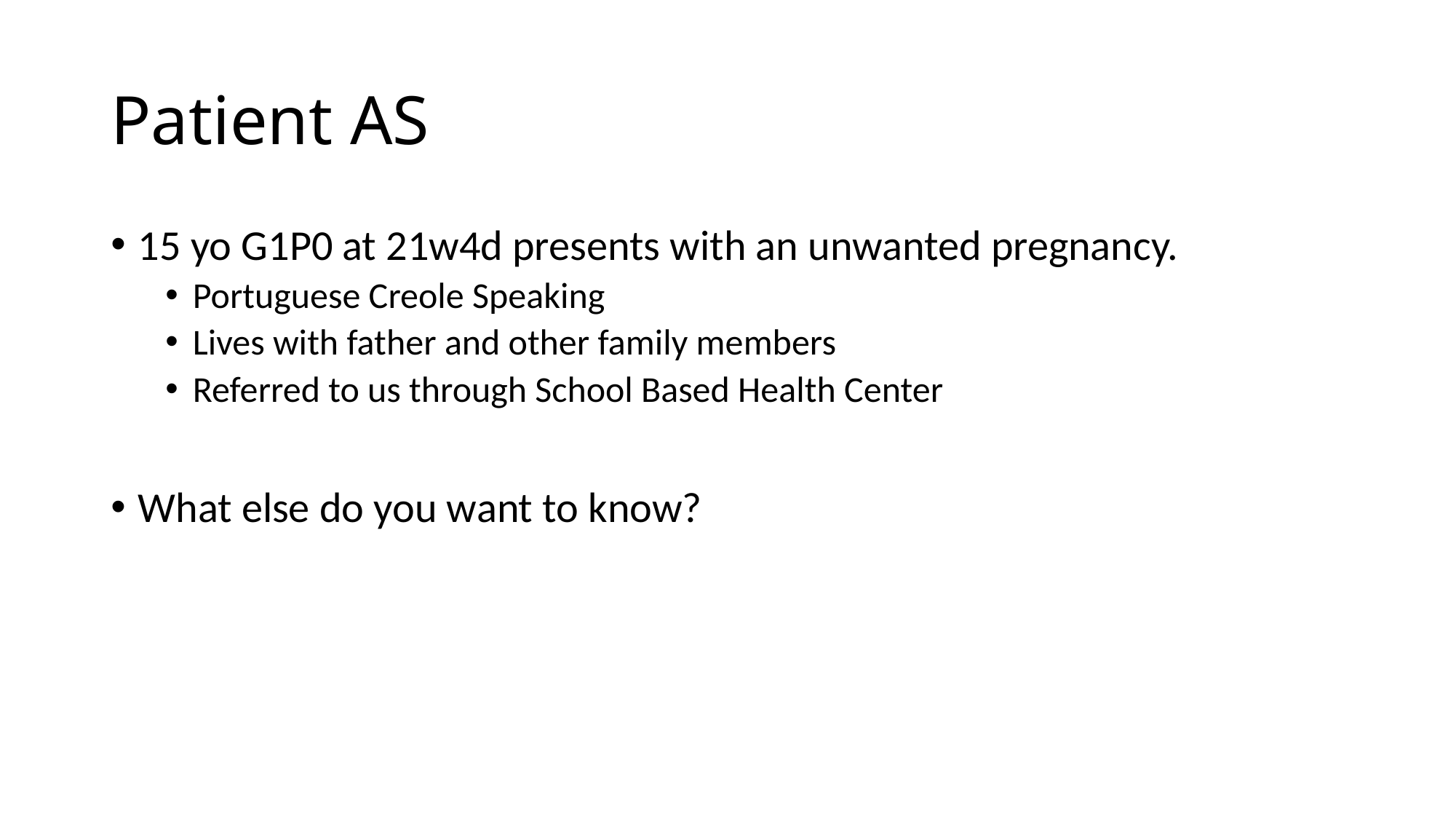

# Patient AS
15 yo G1P0 at 21w4d presents with an unwanted pregnancy.
Portuguese Creole Speaking
Lives with father and other family members
Referred to us through School Based Health Center
What else do you want to know?

## Slide 16
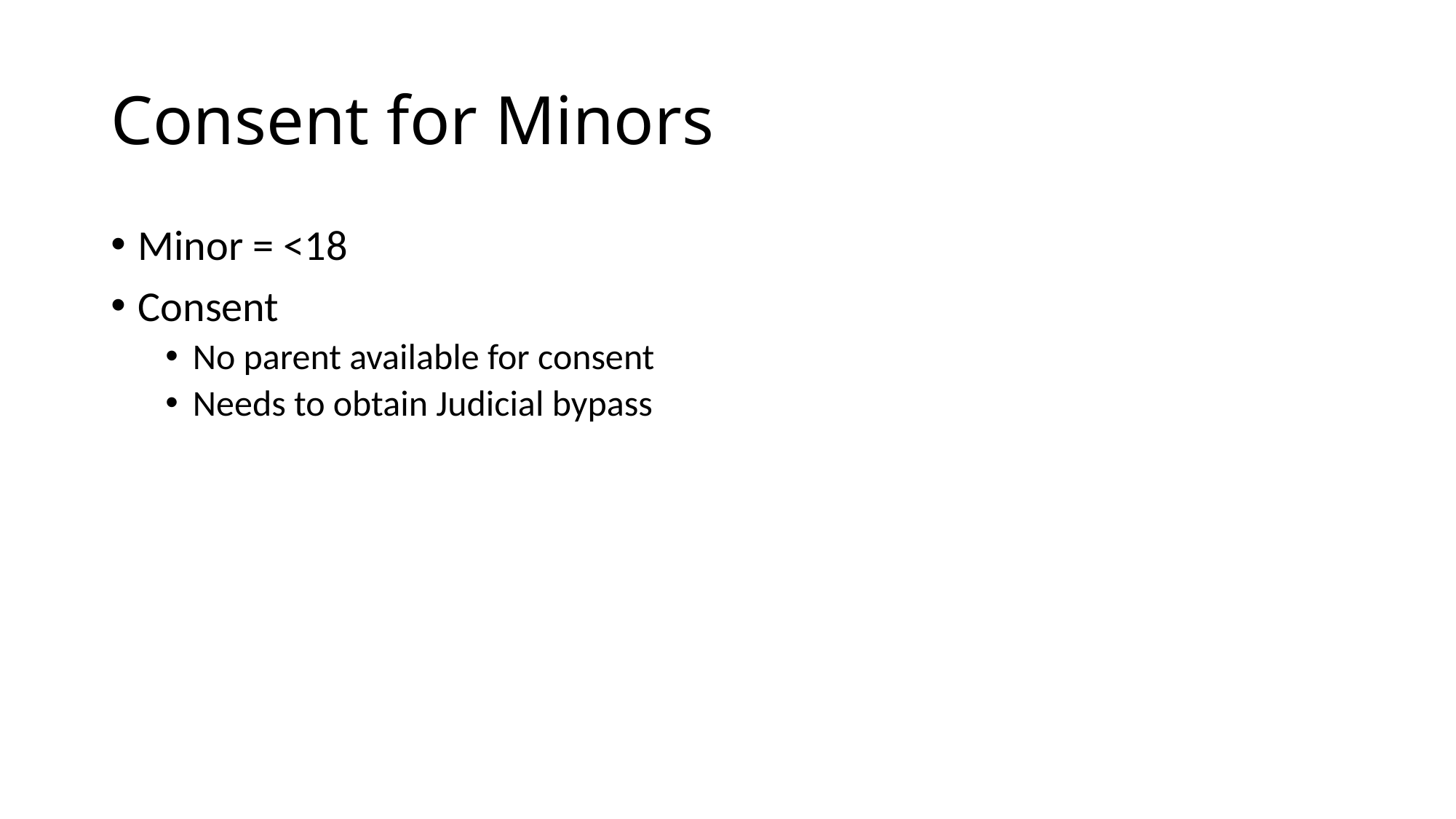

# Consent for Minors
Minor = <18
Consent
No parent available for consent
Needs to obtain Judicial bypass

## Slide 17
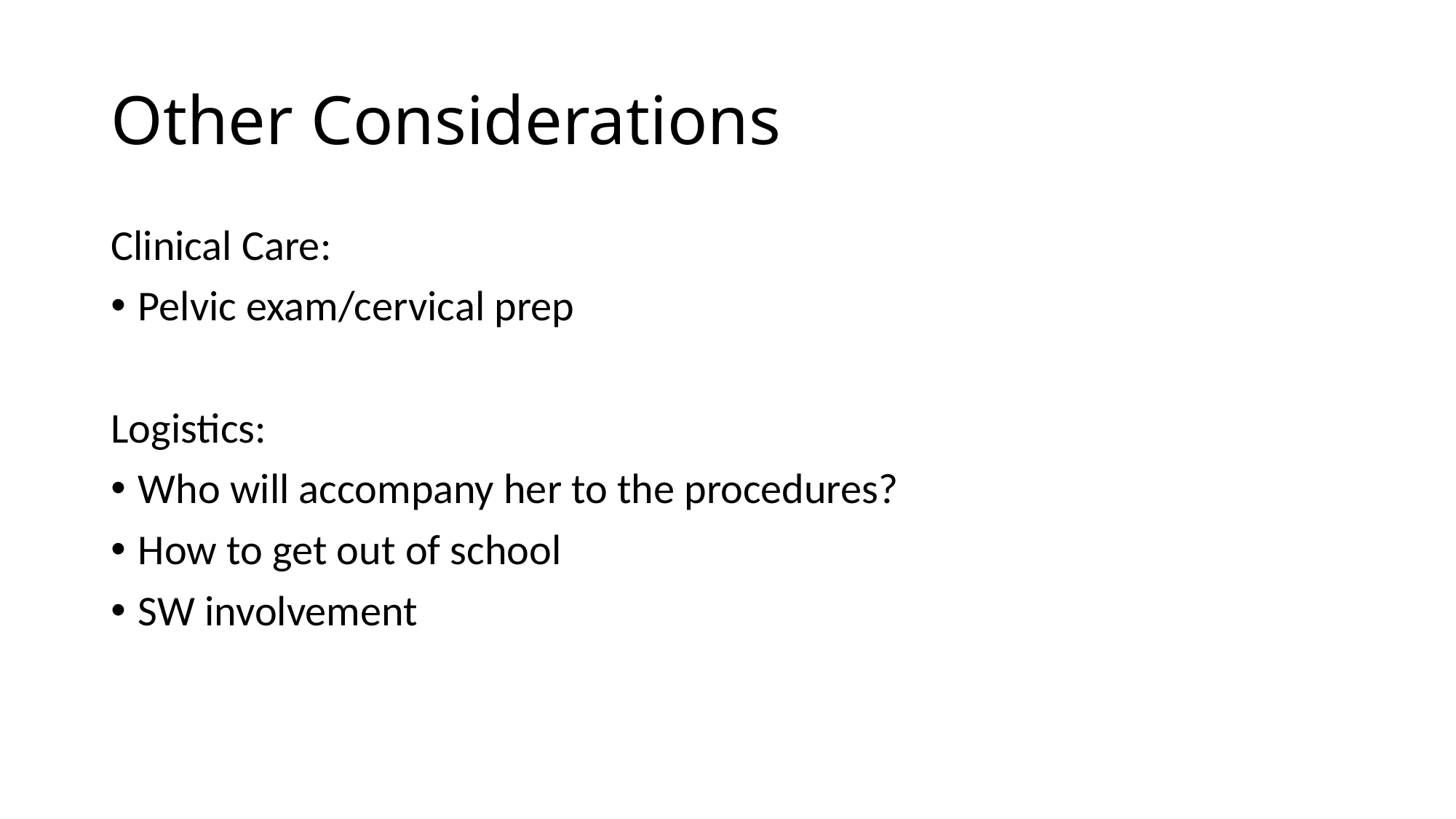

# Other Considerations
Clinical Care:
Pelvic exam/cervical prep
Logistics:
Who will accompany her to the procedures?
How to get out of school
SW involvement

## Slide 18
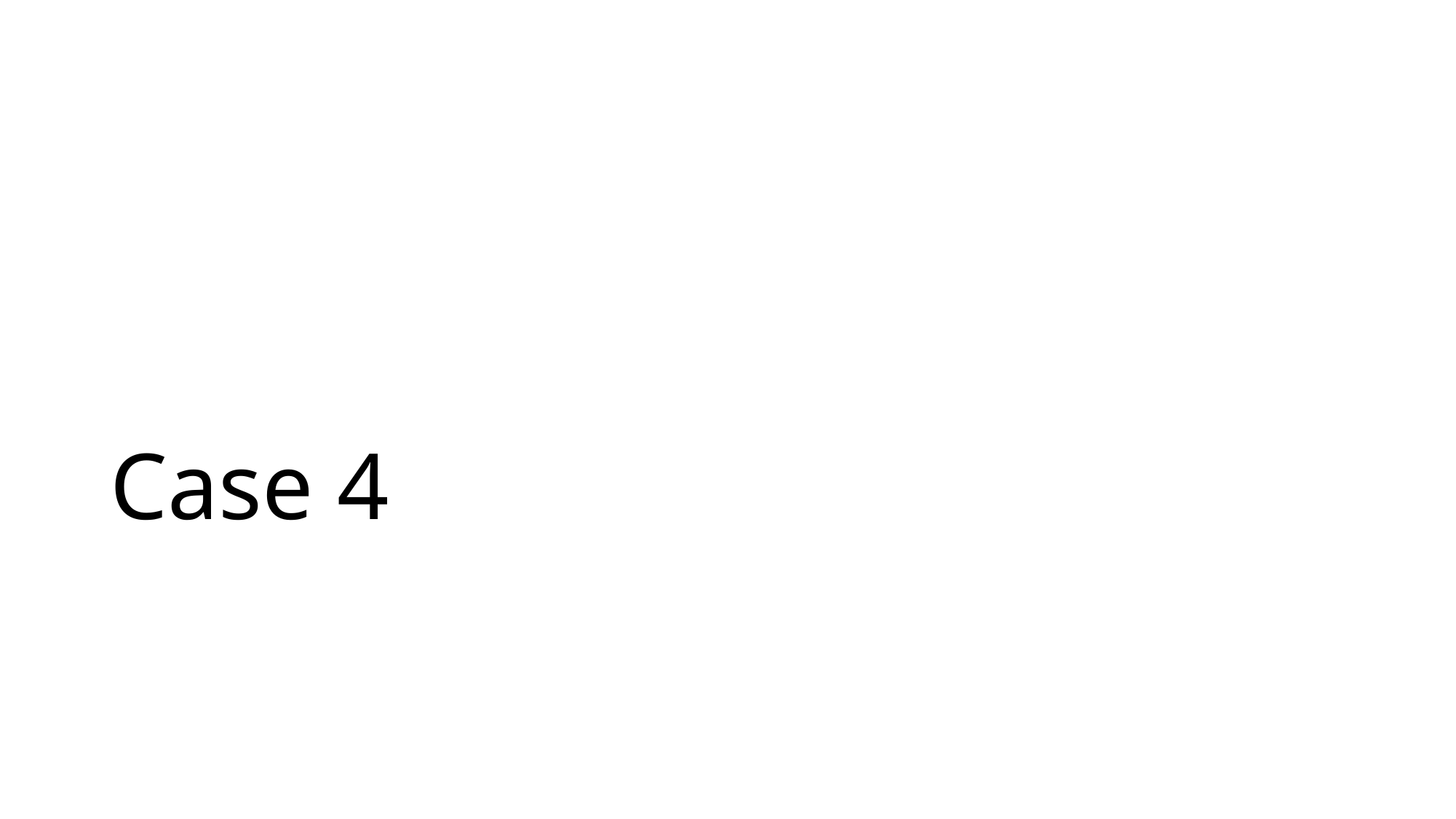

# Case 4

## Slide 19
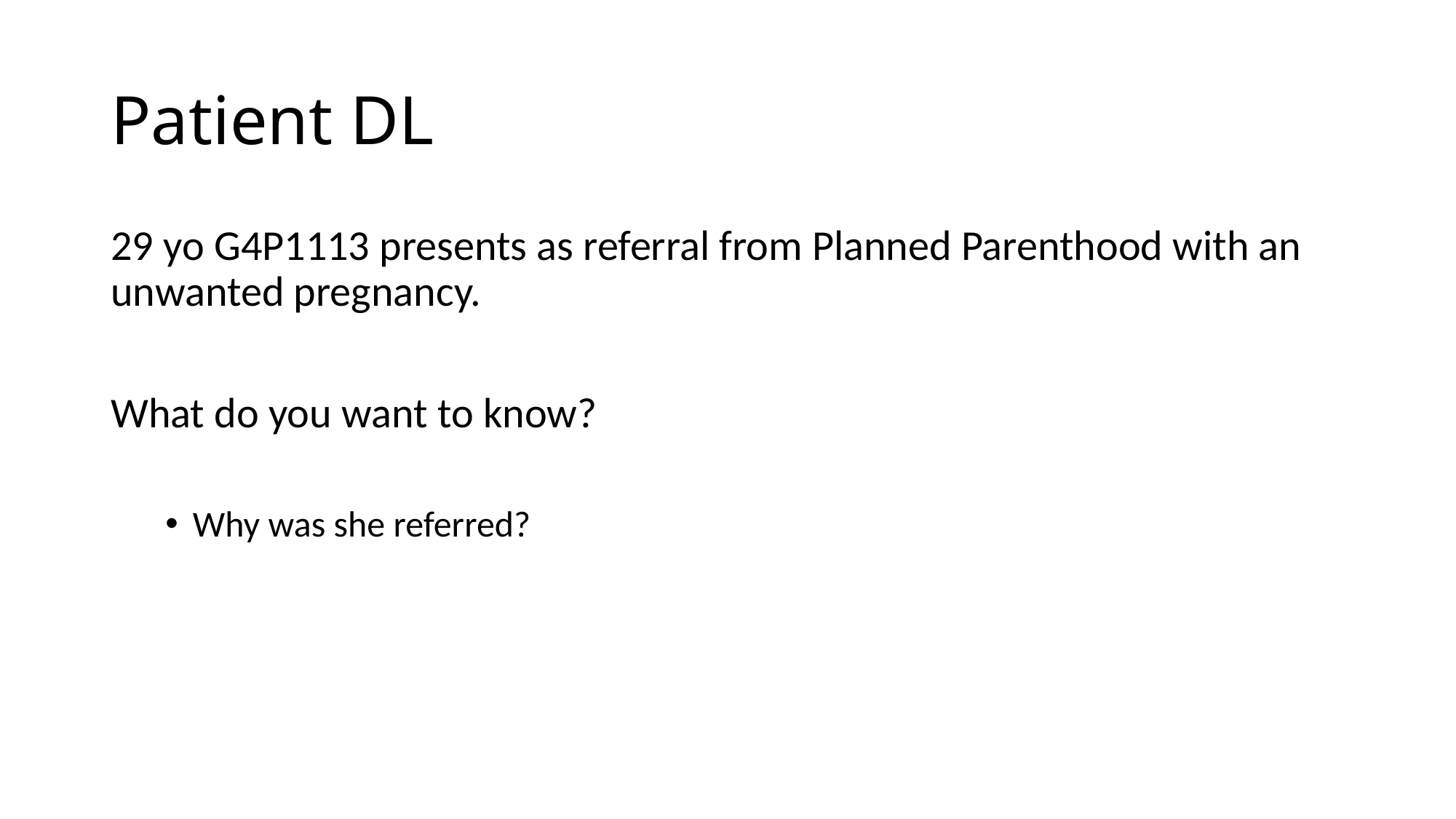

# Patient DL
29 yo G4P1113 presents as referral from Planned Parenthood with an unwanted pregnancy.
What do you want to know?
Why was she referred?

## Slide 20
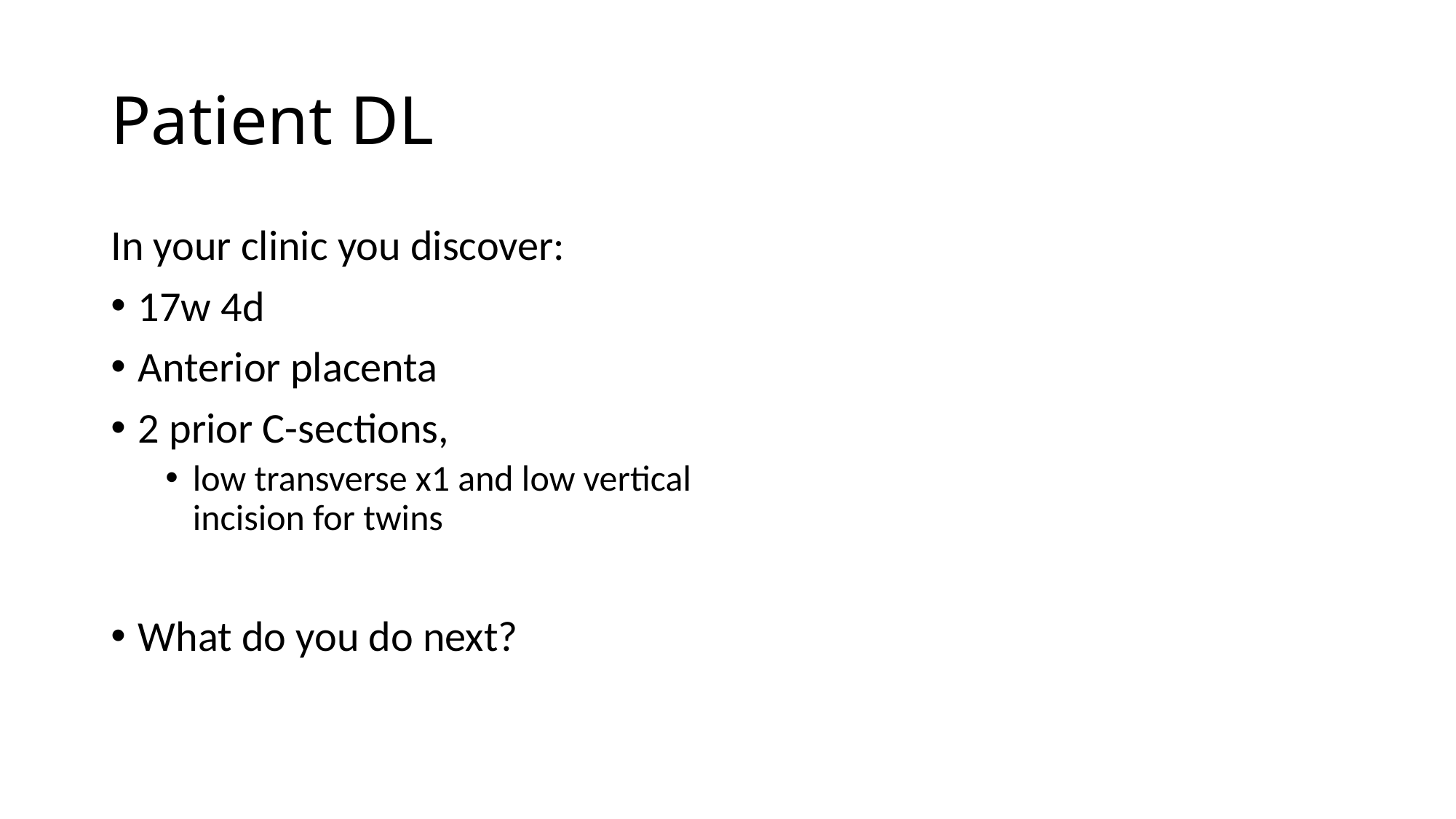

# Patient DL
In your clinic you discover:
17w 4d
Anterior placenta
2 prior C-sections,
low transverse x1 and low vertical incision for twins
What do you do next?

## Slide 21
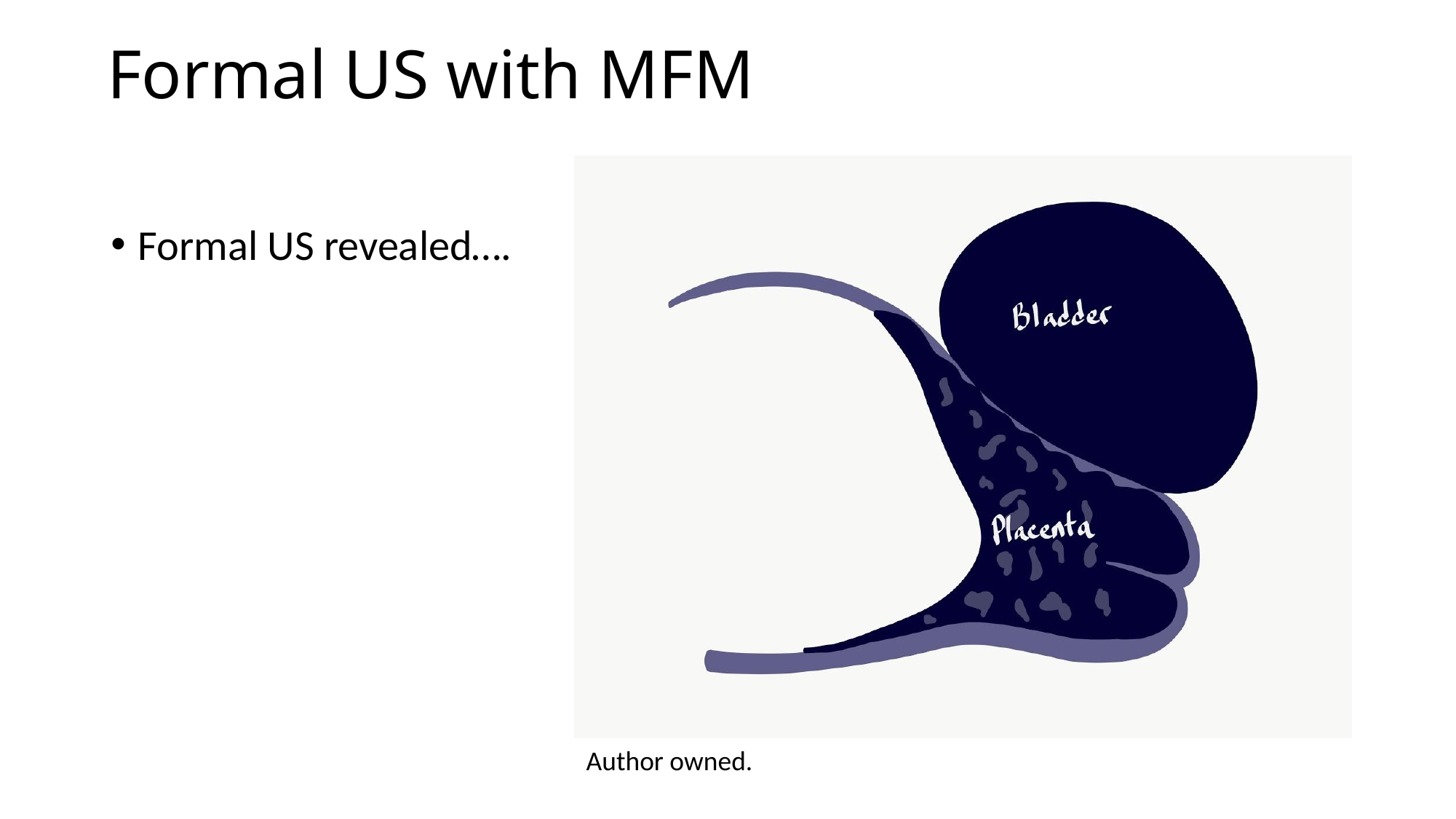

# Formal US with MFM
Formal US revealed….
Author owned.

## Slide 22
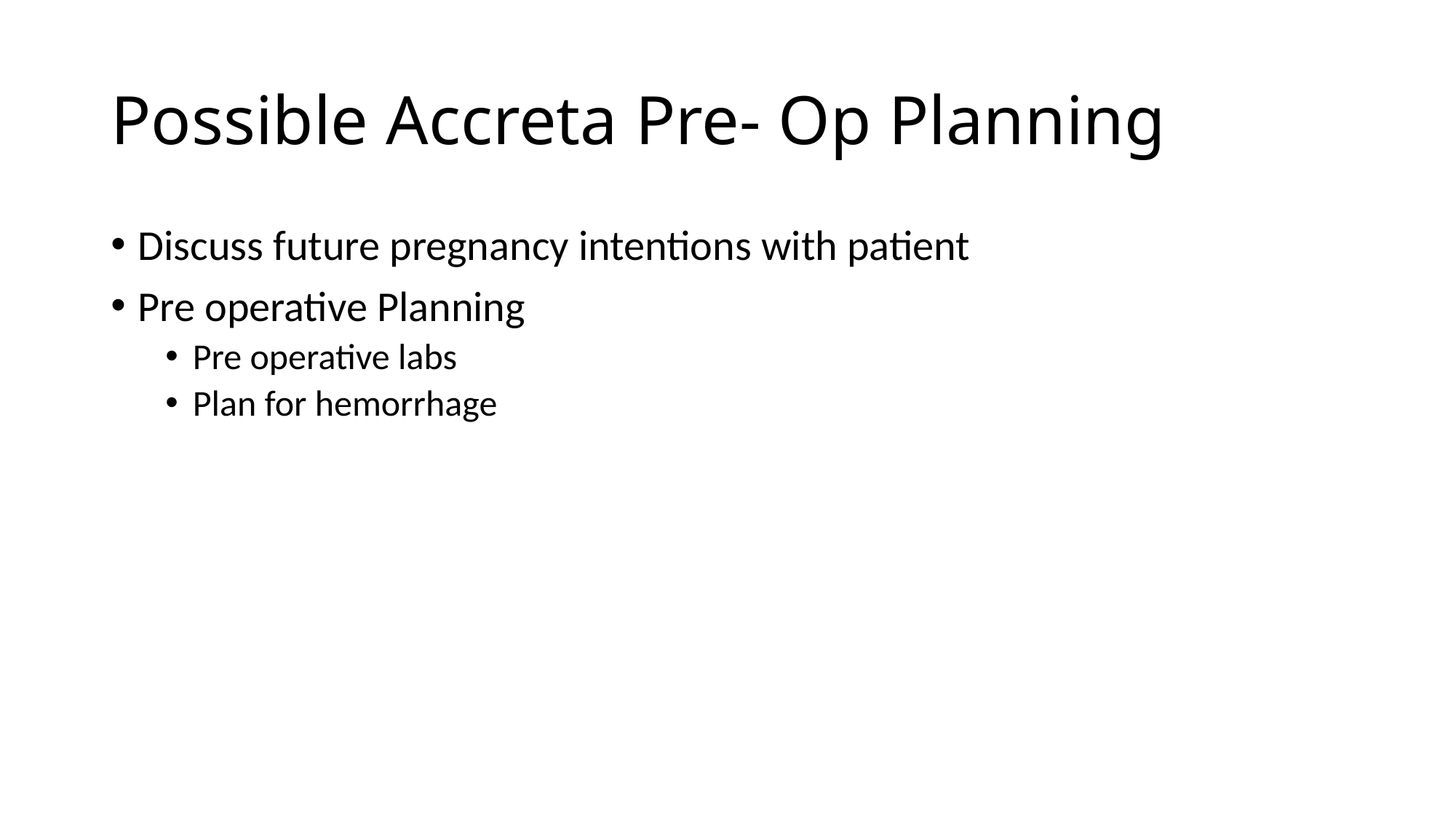

# Possible Accreta Pre- Op Planning
Discuss future pregnancy intentions with patient
Pre operative Planning
Pre operative labs
Plan for hemorrhage

## Slide 23
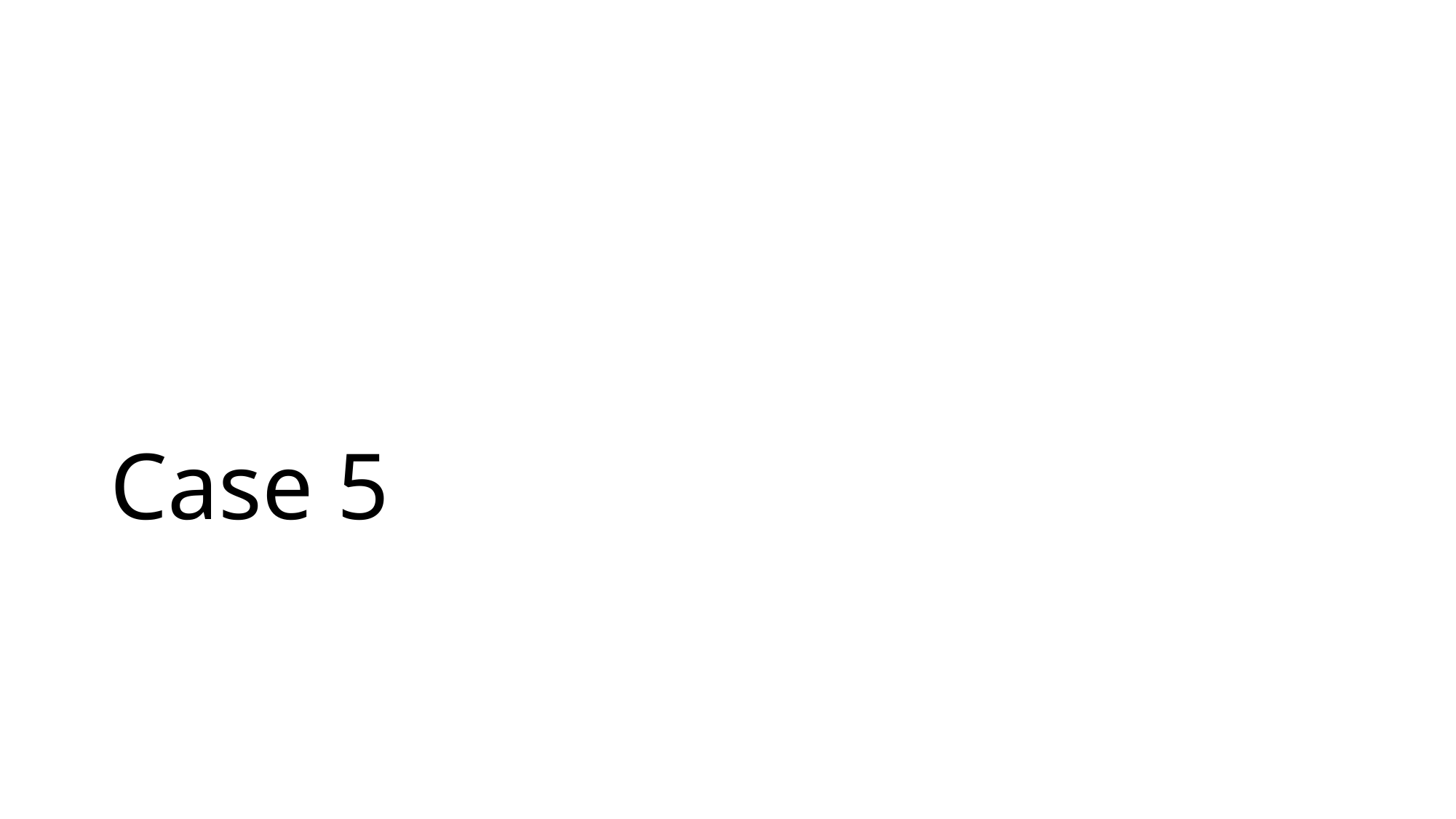

# Case 5

## Slide 24
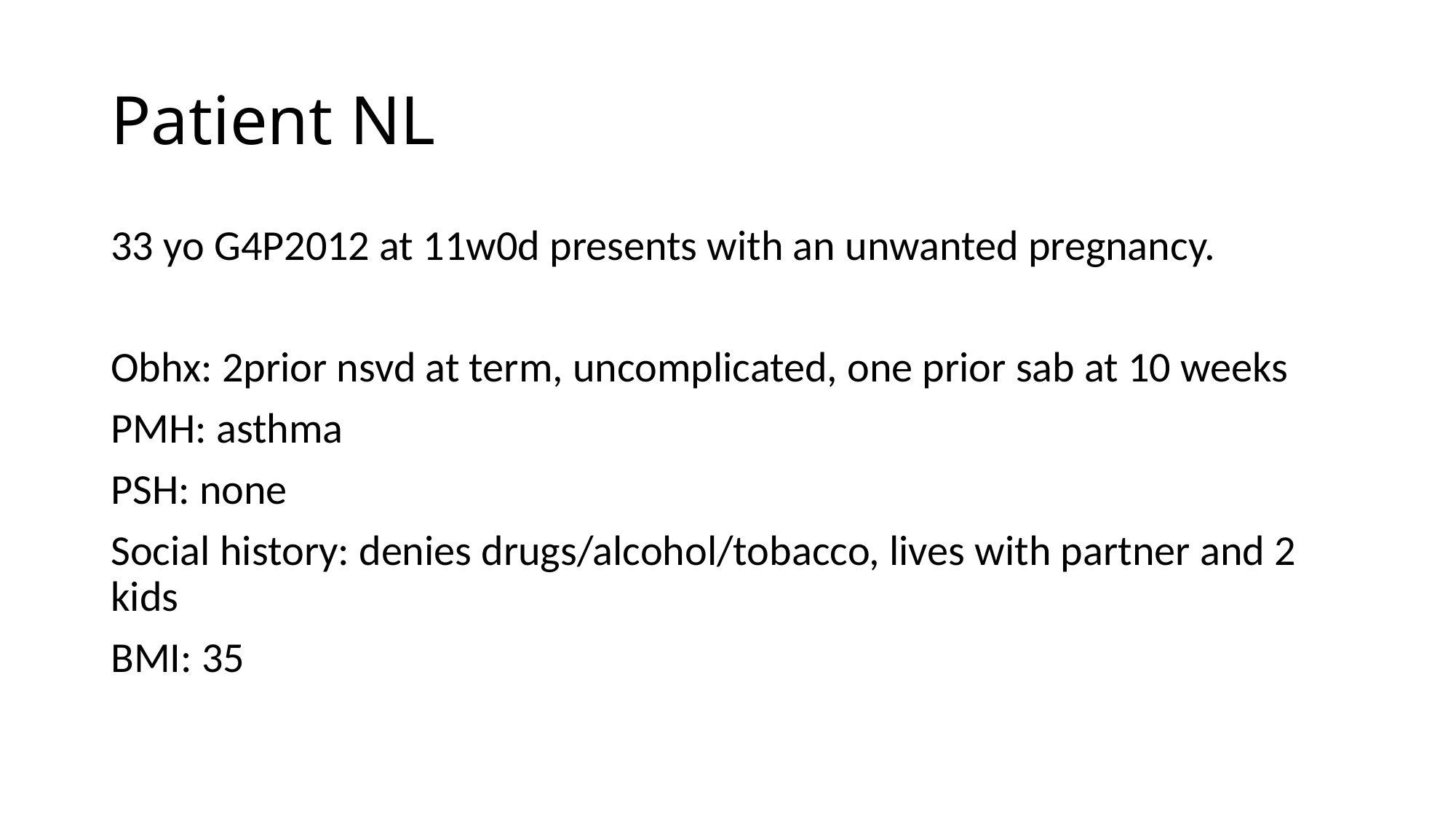

# Patient NL
33 yo G4P2012 at 11w0d presents with an unwanted pregnancy.
Obhx: 2prior nsvd at term, uncomplicated, one prior sab at 10 weeks
PMH: asthma
PSH: none
Social history: denies drugs/alcohol/tobacco, lives with partner and 2 kids
BMI: 35

## Slide 25
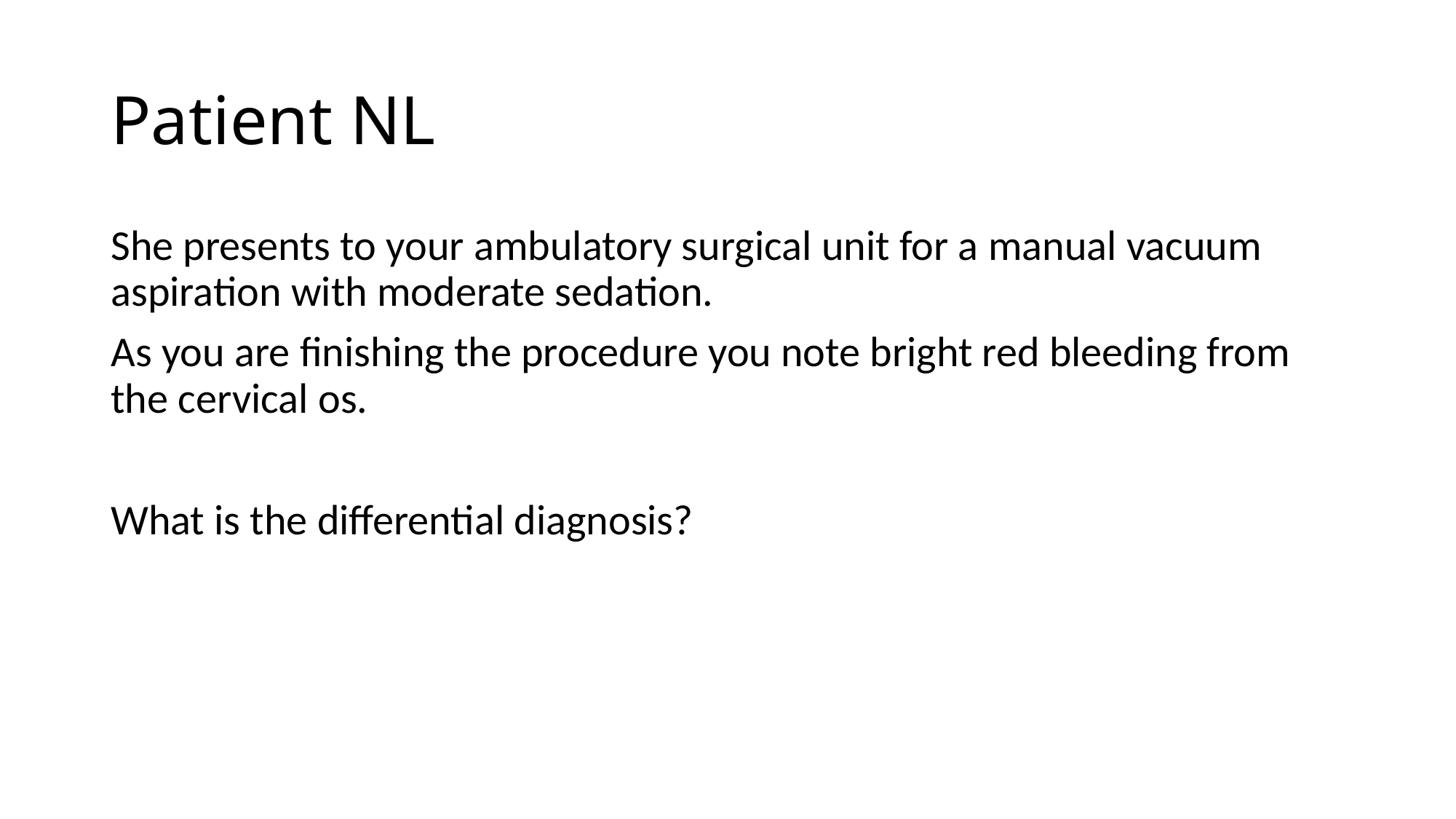

# Patient NL
She presents to your ambulatory surgical unit for a manual vacuum aspiration with moderate sedation.
As you are finishing the procedure you note bright red bleeding from the cervical os.
What is the differential diagnosis?

## Slide 26
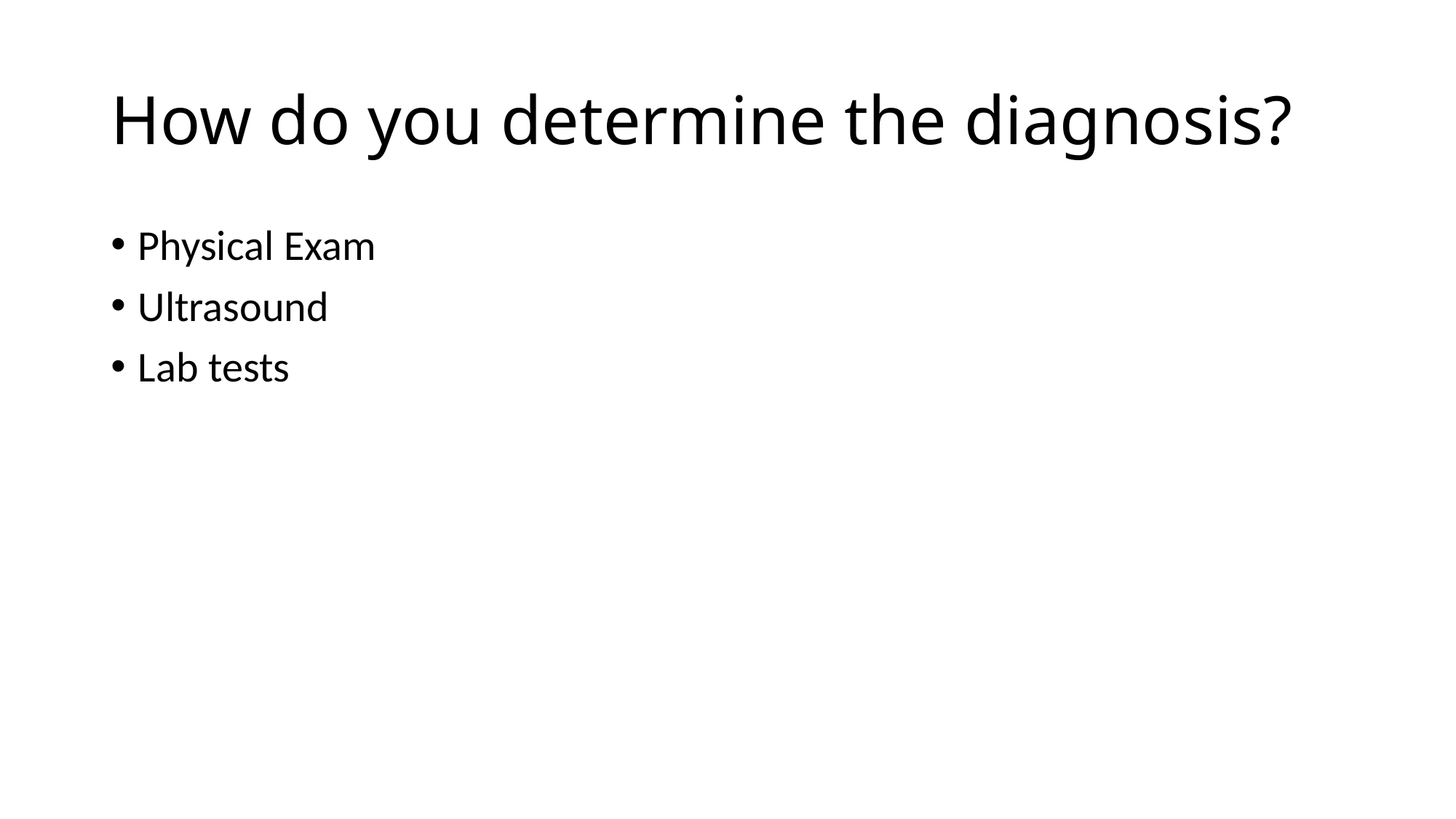

# How do you determine the diagnosis?
Physical Exam
Ultrasound
Lab tests

## Slide 27
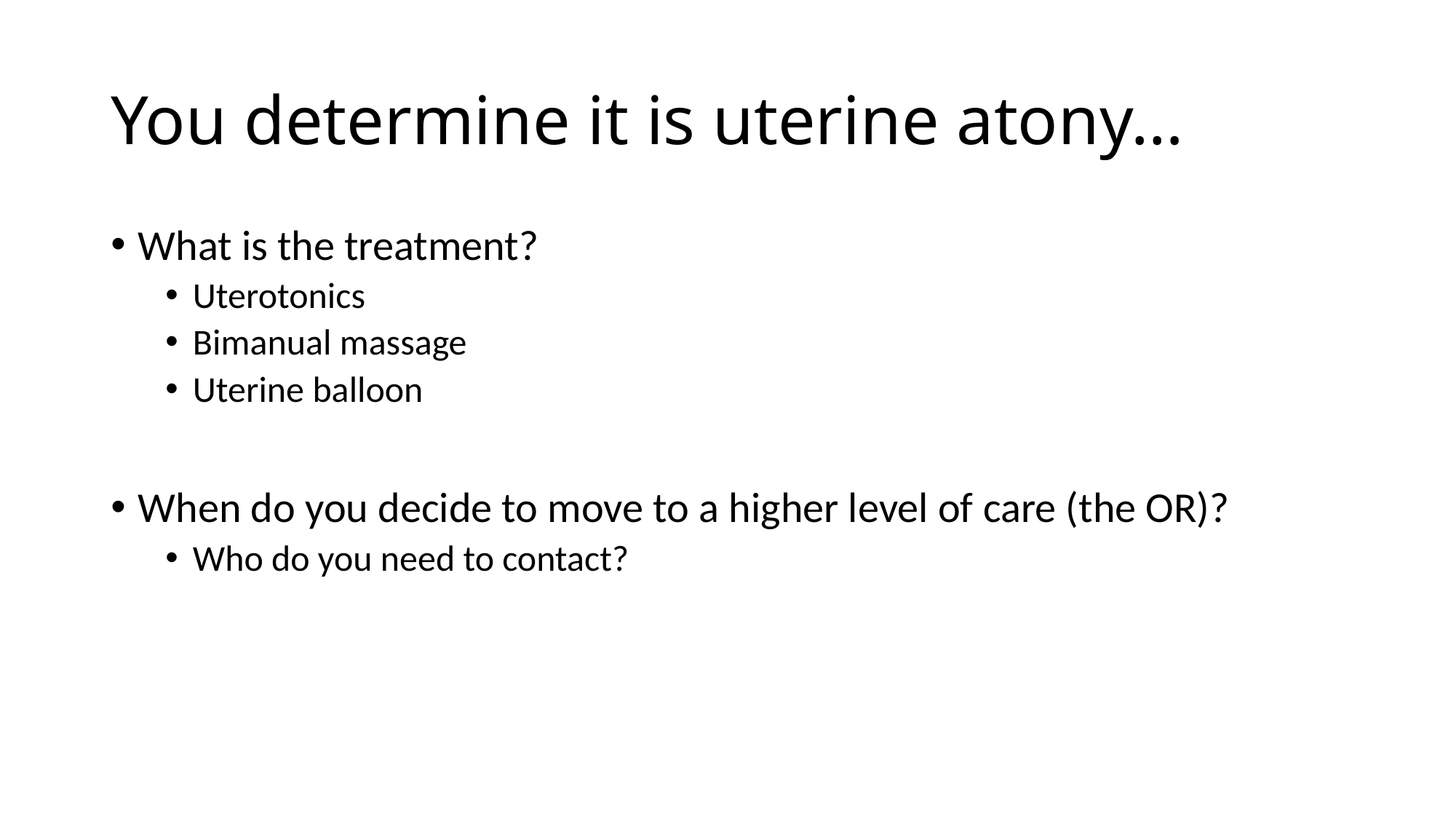

# You determine it is uterine atony…
What is the treatment?
Uterotonics
Bimanual massage
Uterine balloon
When do you decide to move to a higher level of care (the OR)?
Who do you need to contact?

## Slide 28
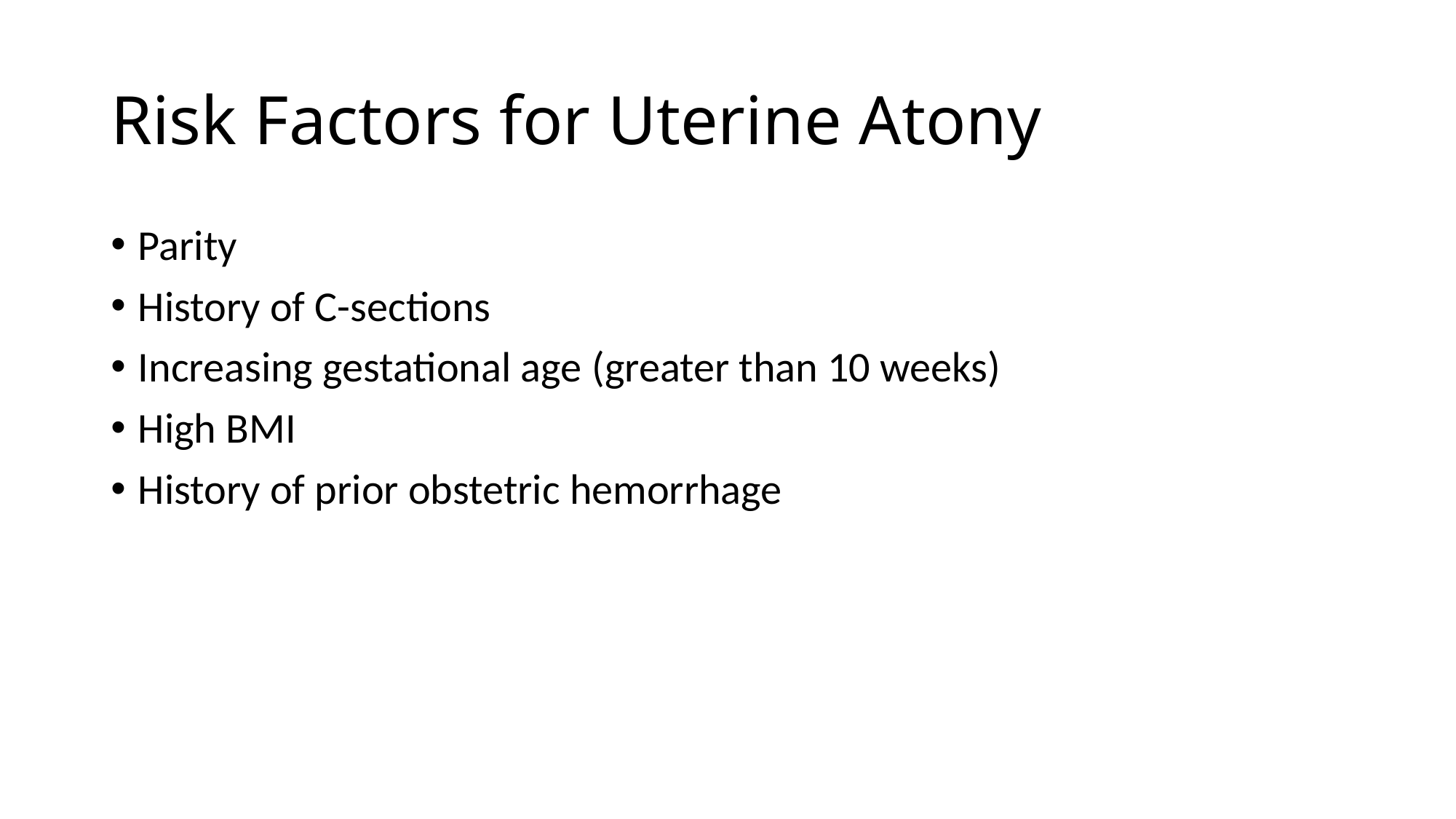

# Risk Factors for Uterine Atony
Parity
History of C-sections
Increasing gestational age (greater than 10 weeks)
High BMI
History of prior obstetric hemorrhage

## Slide 29
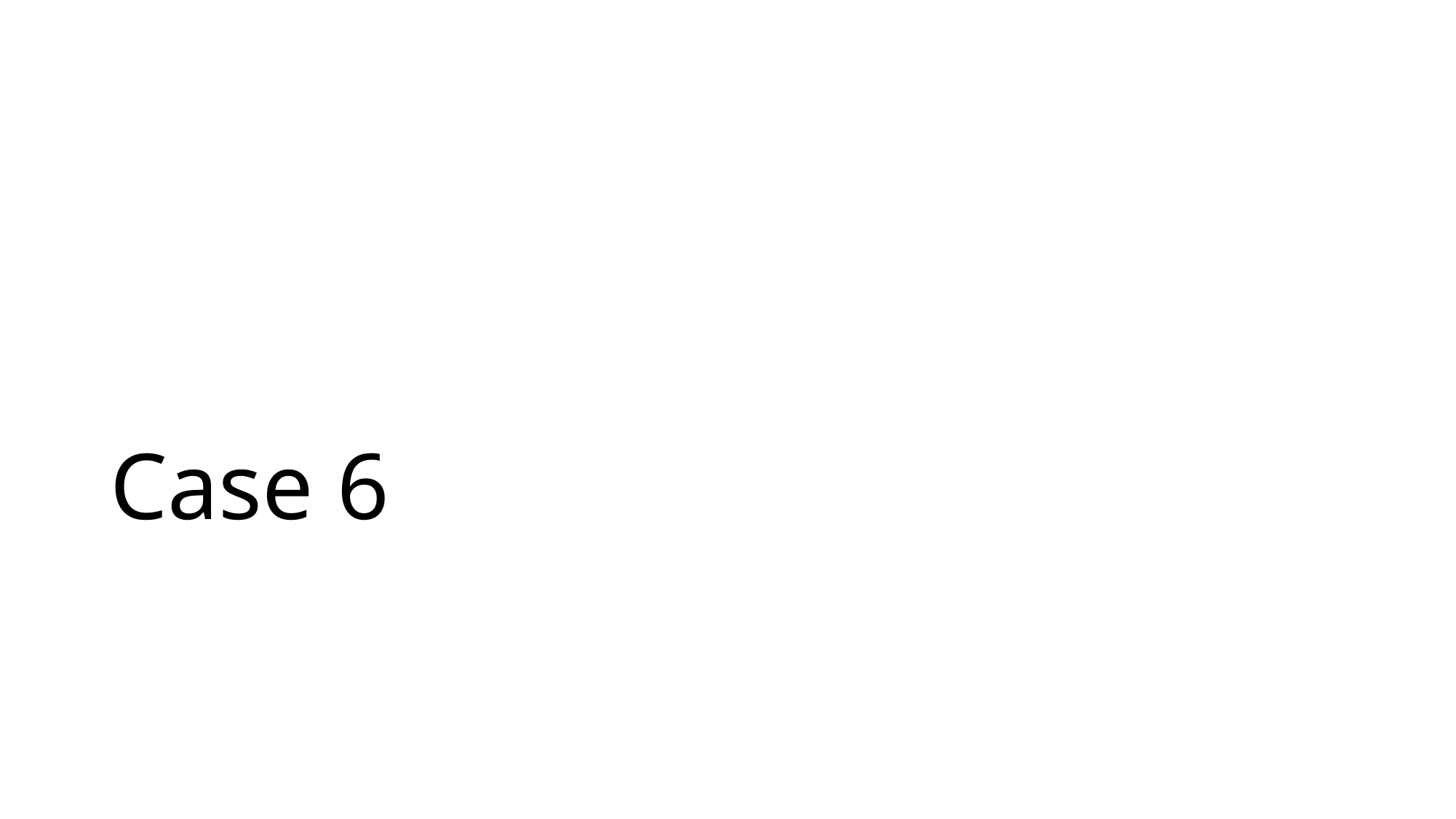

# Case 6

## Slide 30
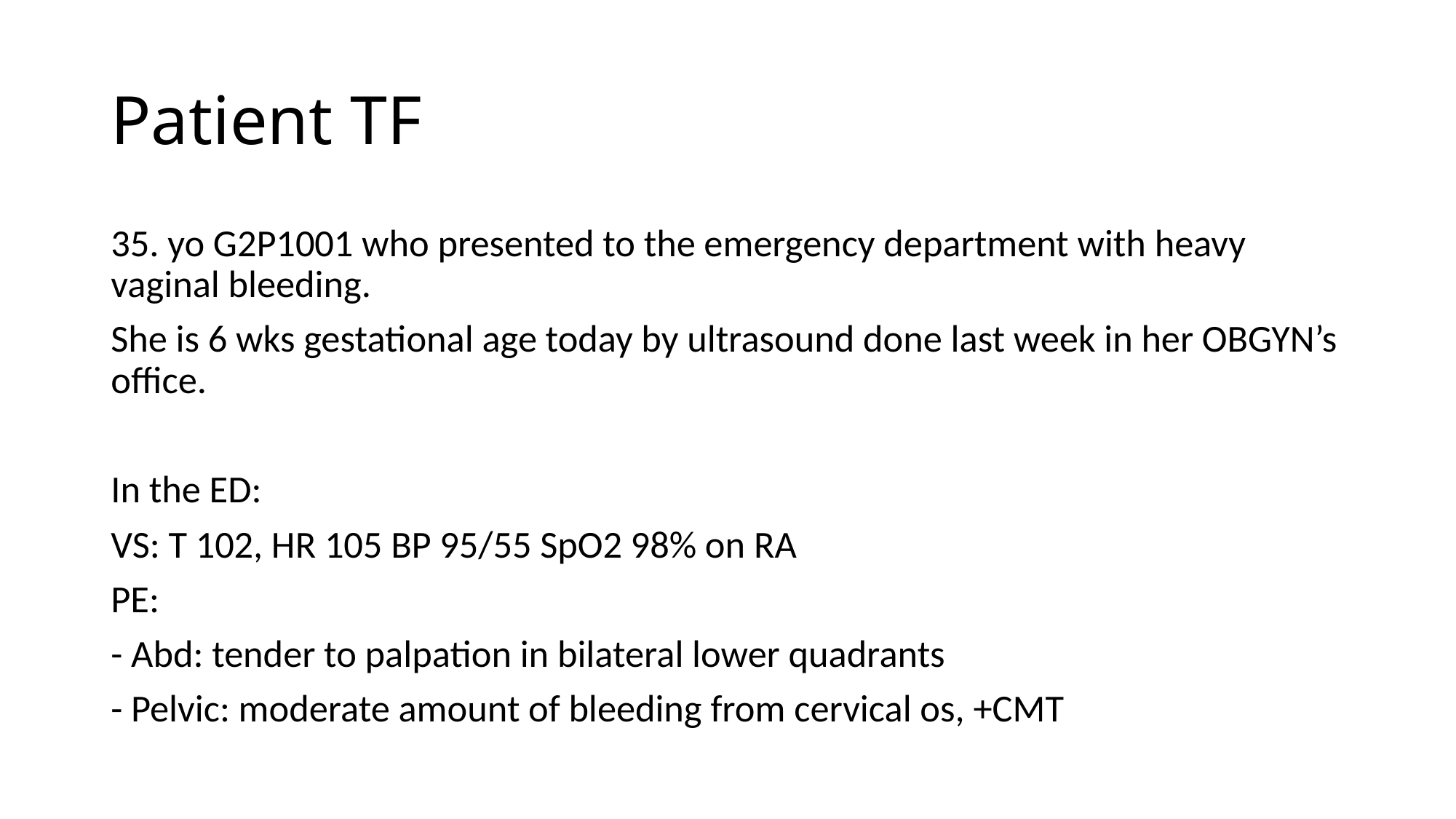

# Patient TF
35. yo G2P1001 who presented to the emergency department with heavy vaginal bleeding.
She is 6 wks gestational age today by ultrasound done last week in her OBGYN’s office.
In the ED:
VS: T 102, HR 105 BP 95/55 SpO2 98% on RA
PE:
- Abd: tender to palpation in bilateral lower quadrants
- Pelvic: moderate amount of bleeding from cervical os, +CMT

## Slide 31
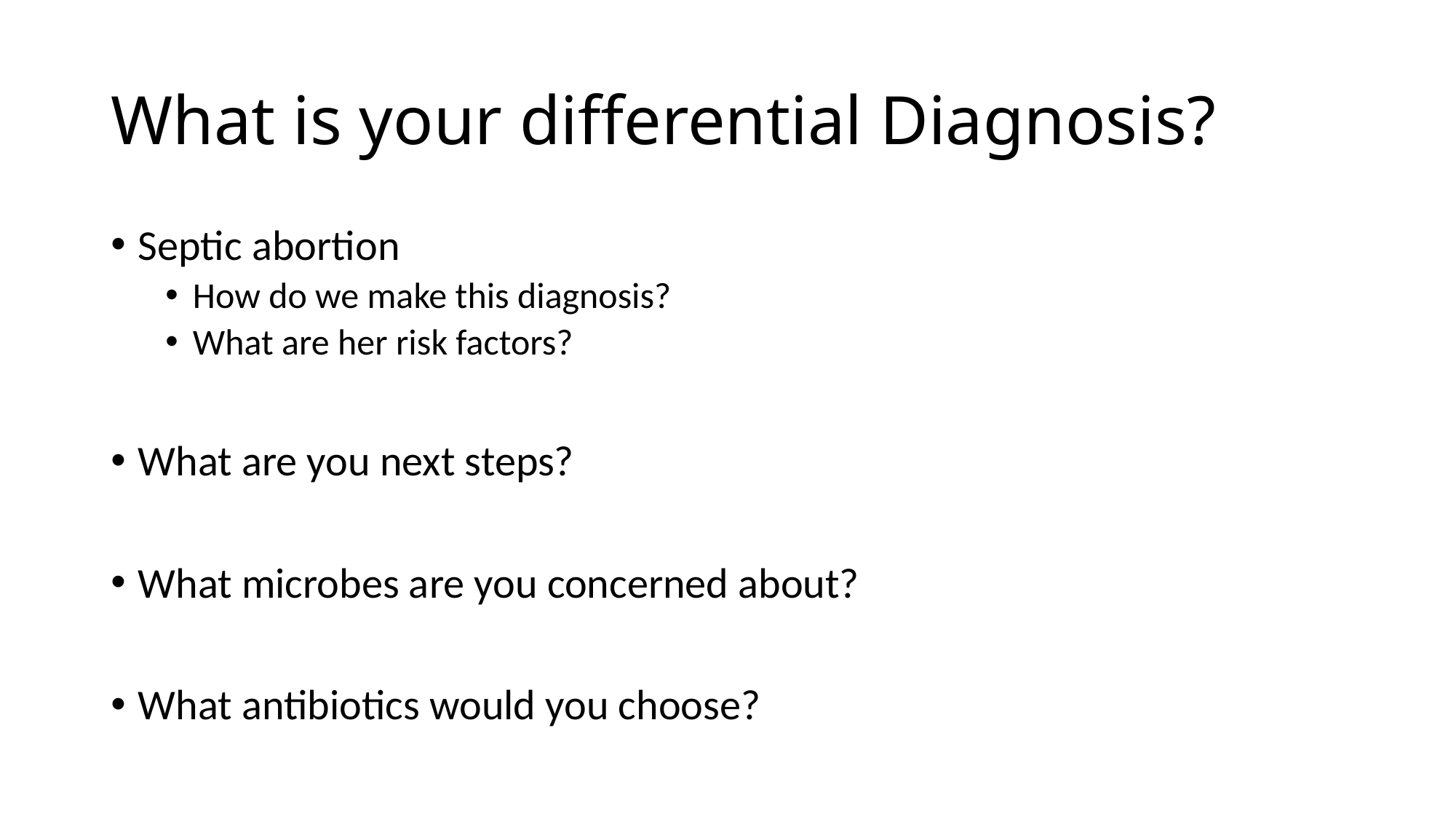

# What is your differential Diagnosis?
Septic abortion
How do we make this diagnosis?
What are her risk factors?
What are you next steps?
What microbes are you concerned about?
What antibiotics would you choose?

## Slide 32
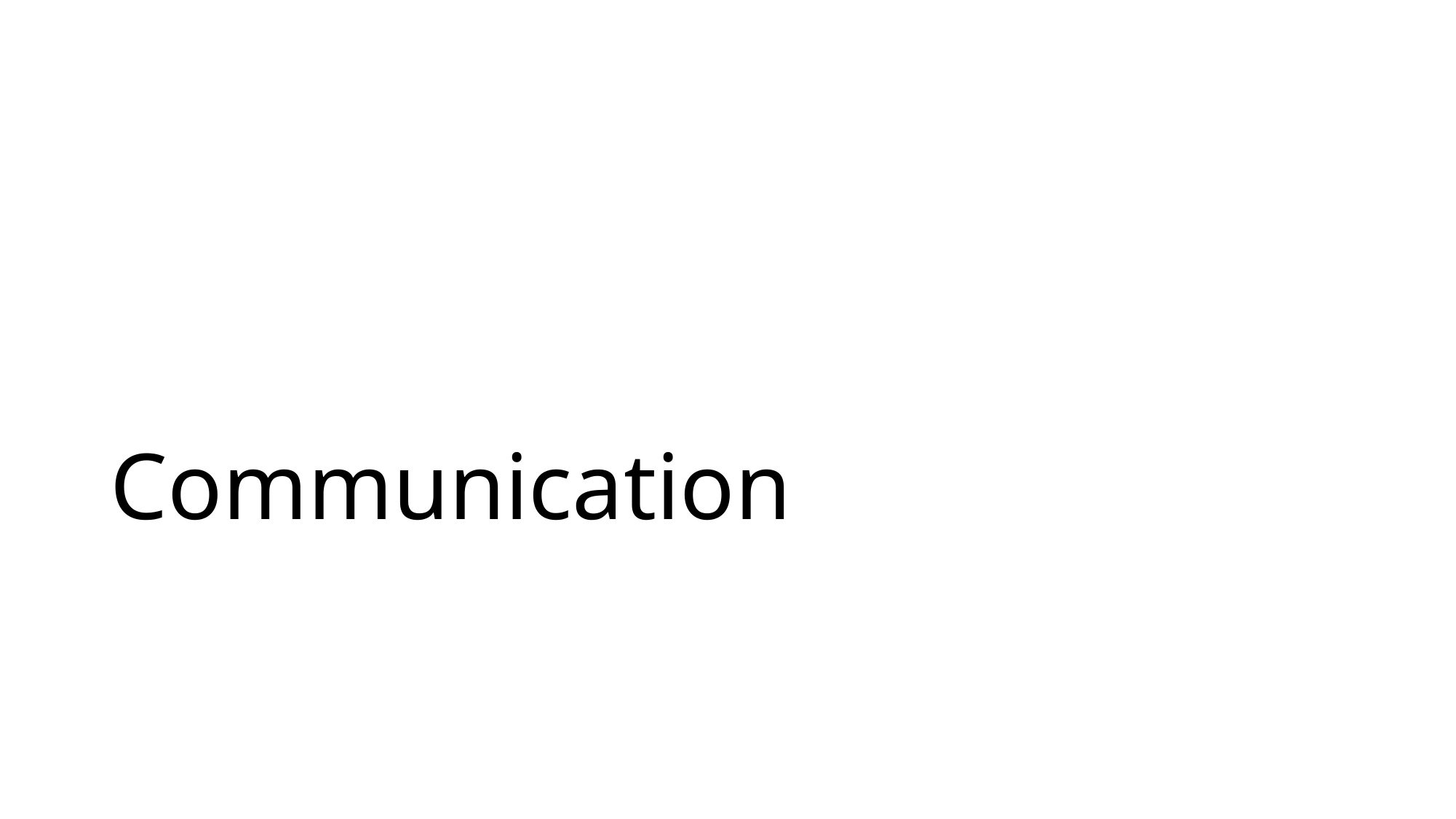

# Communication

## Slide 33
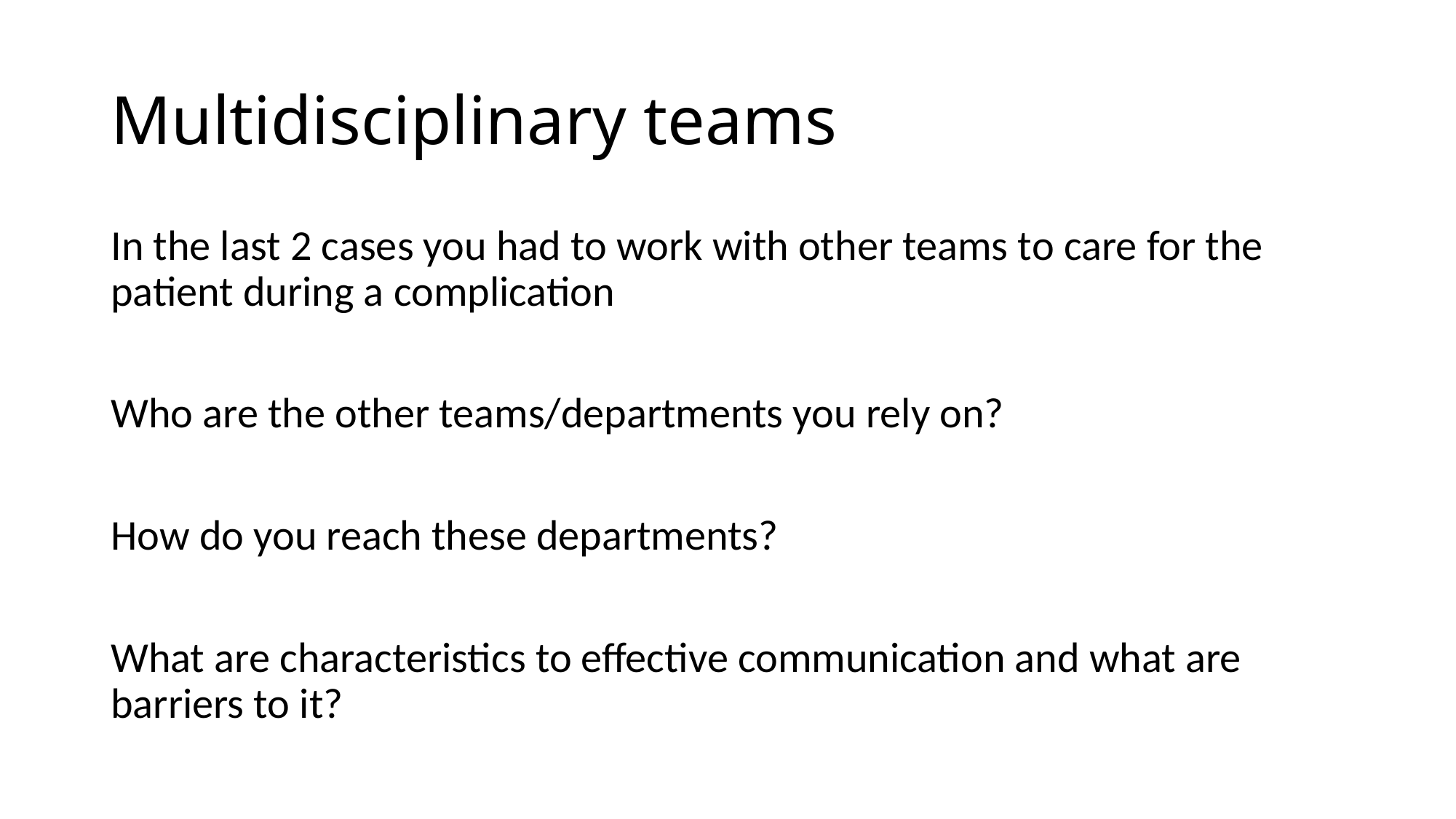

# Multidisciplinary teams
In the last 2 cases you had to work with other teams to care for the patient during a complication
Who are the other teams/departments you rely on?
How do you reach these departments?
What are characteristics to effective communication and what are barriers to it?

## Slide 34
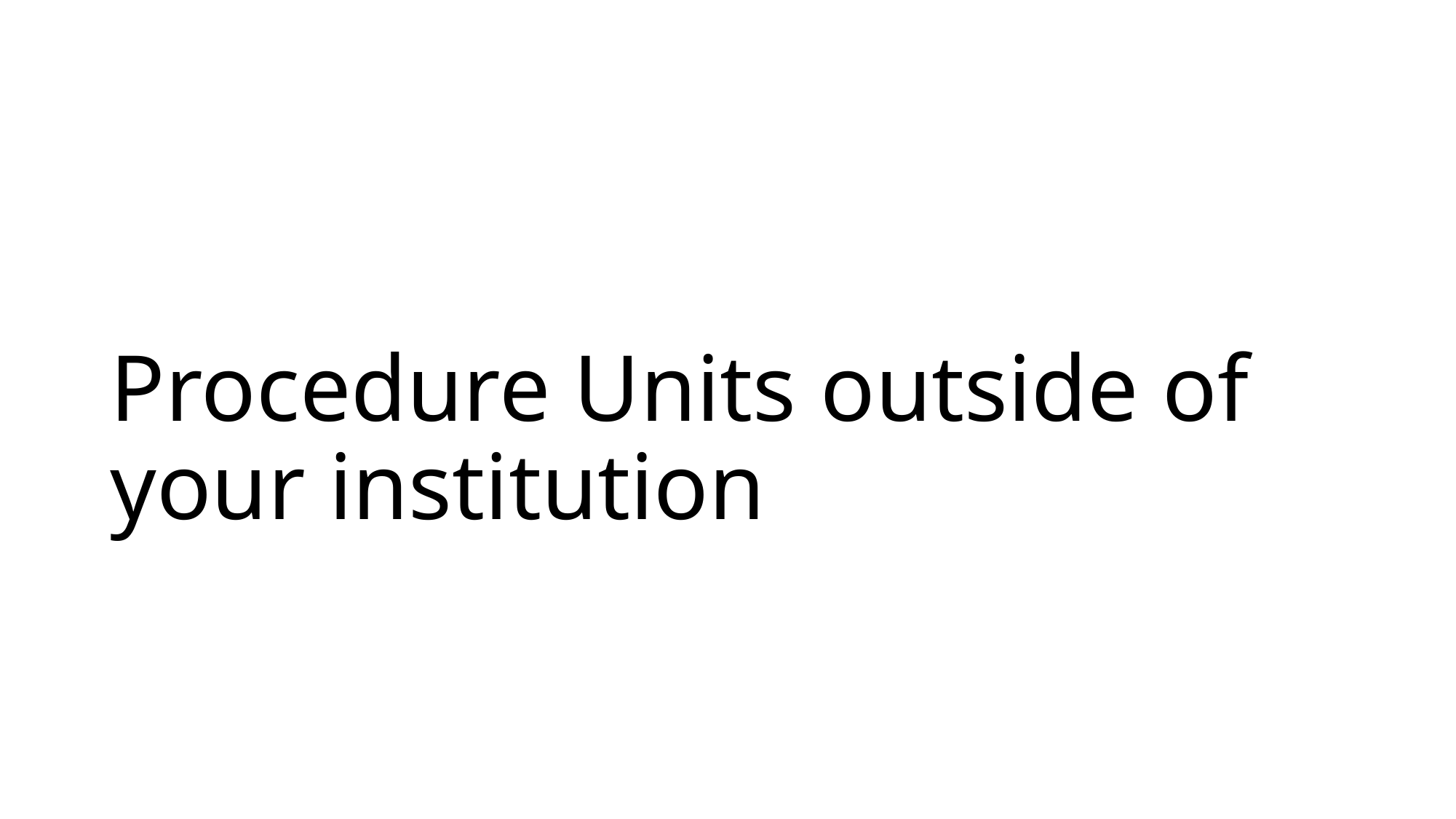

# Procedure Units outside of your institution

## Slide 35
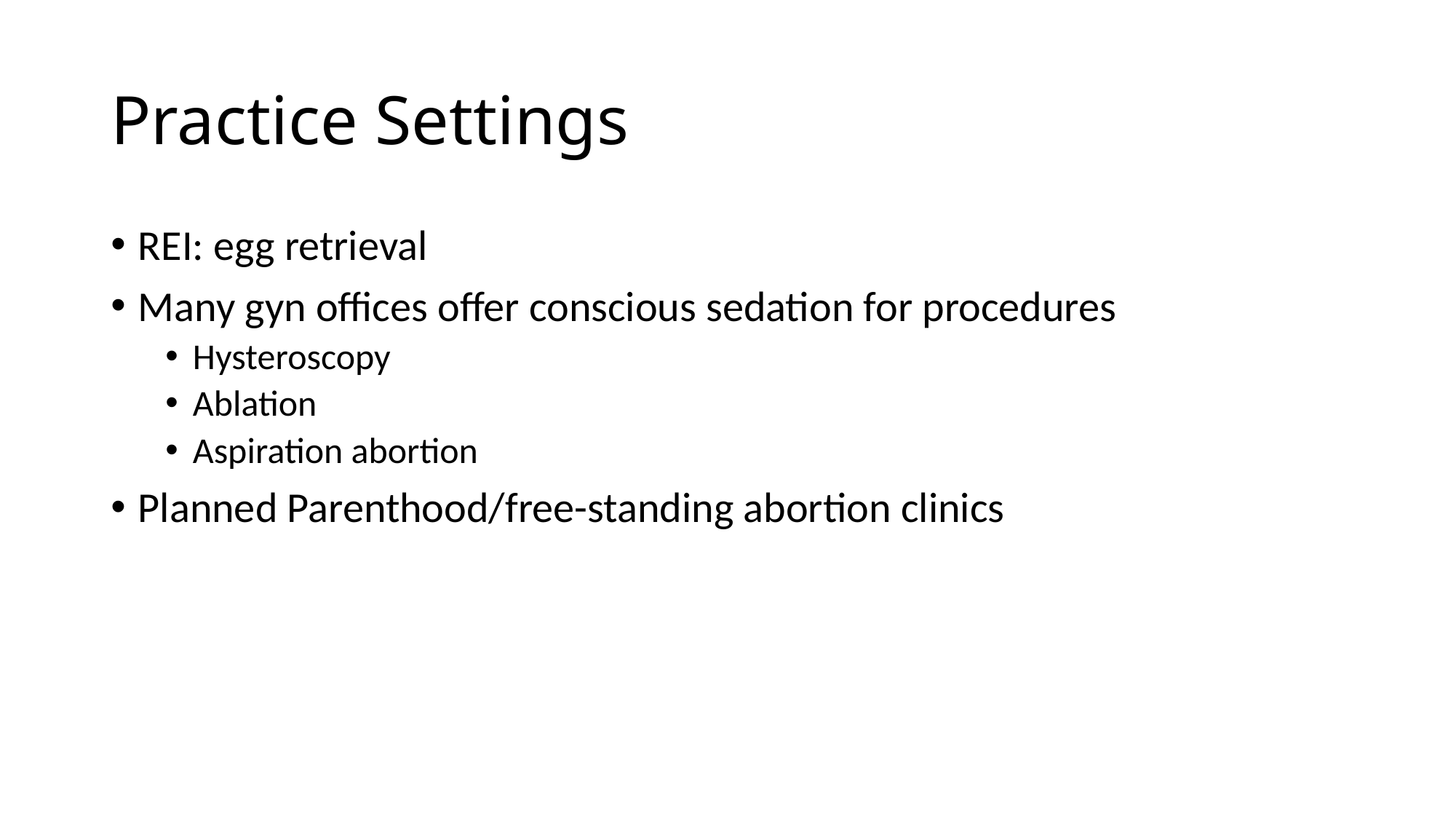

# Practice Settings
REI: egg retrieval
Many gyn offices offer conscious sedation for procedures
Hysteroscopy
Ablation
Aspiration abortion
Planned Parenthood/free-standing abortion clinics

## Slide 36
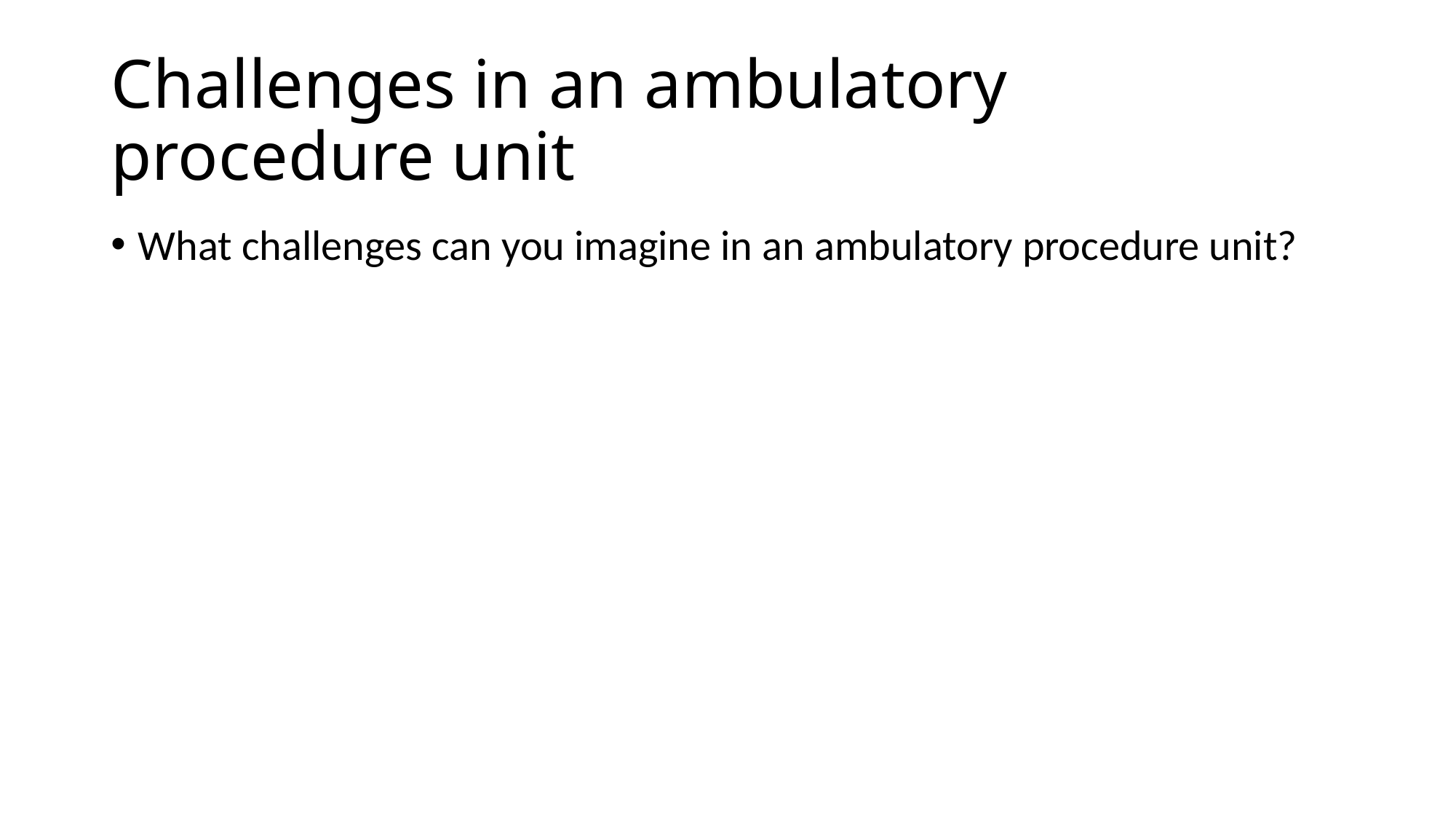

# Challenges in an ambulatory procedure unit
What challenges can you imagine in an ambulatory procedure unit?

## Slide 37
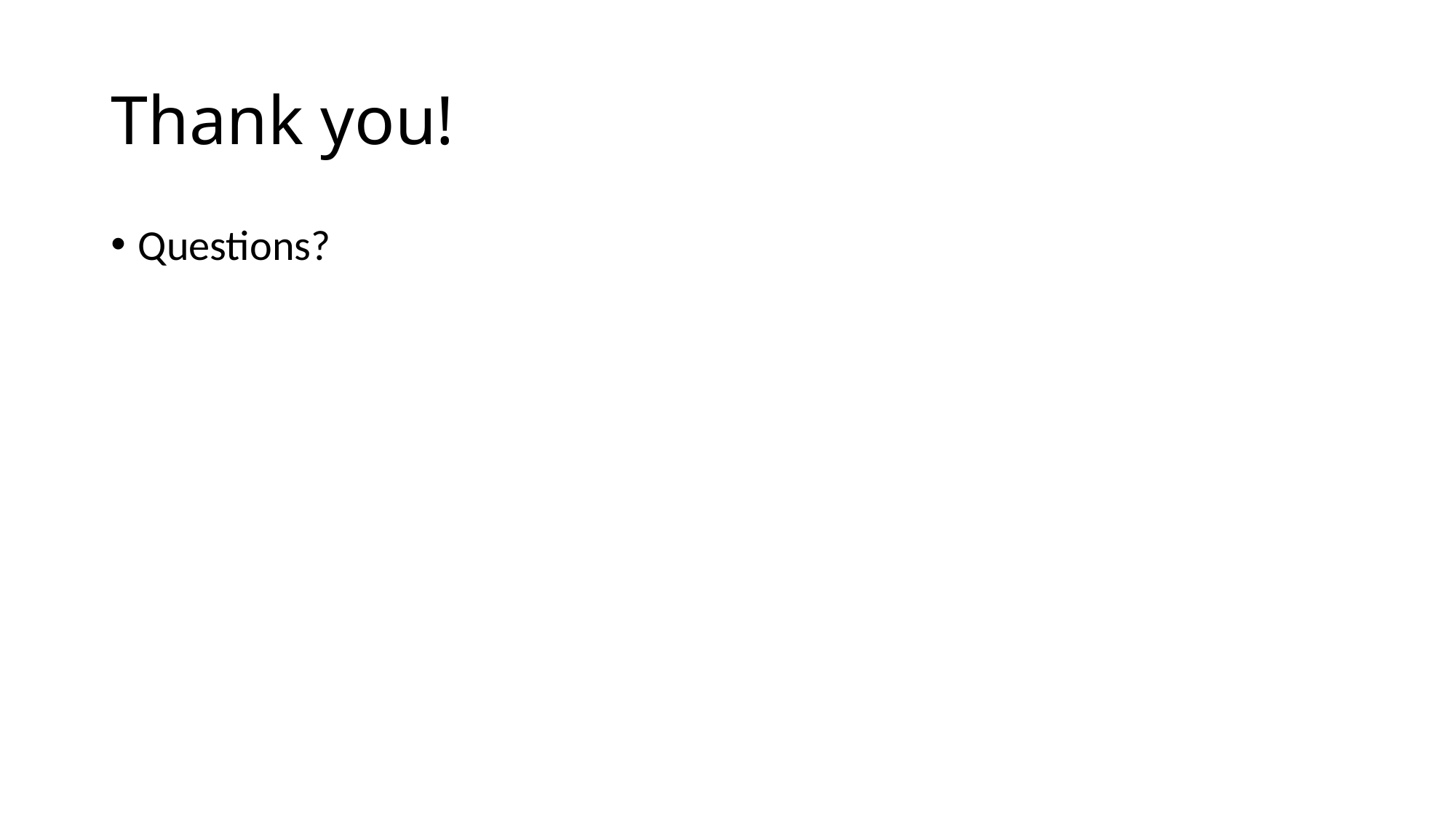

# Thank you!
Questions?
