# Supplementary material for: First-Trimester Abortion Complications: Simulation Cases for OB/GYN Residents in Sepsis and Hemorrhage
Source: MedEdPORTAL. 2020 Oct 16;16:10995. doi: 10.15766/mep_2374-8265.10995 (PMC7566226; doi:10.15766/mep_2374-8265.10995)
Supplement: Supplementary file 1 — Sepsis Simulation Case.docxHemorrhage Simulation Case.docxSimulation Images.docxPresimulation Didactic Lecture.pptxSepsis Critical Action Checklist.docxHemorrhage Critical Action Checklist.docxSepsis Debriefing Guide.docxHemorrhage Debriefing Guide.docxSepsis Postsimulation Debrief Didactic.pptxSepsis Pre-and Postsurvey.docxHemorrhage Pre-and Postsurvey.docx [file mep_2374-8265.10995-s001.zip › E. Sepsis Critical Action Checklist.docx]

**Learner(s): ______________________________ Assessor(s): ______________________________**

**Sepsis Simulation**

**Critical Action Checklist**

- Ascertain complete history and physical from Emergency Department (ED) report
- Order appropriate labs for a patient presenting to the ED for complications due to a medication abortion and meeting criteria for sepsis with possible shock and end organ damage
  - CBC
  - CMP
  - Urine pregnancy test
  - Lactate
  - Venous blood gas
  - Coagulation panel
  - Type and screen
  - Blood Culture
- Perform bimanual and speculum exam to evaluate uterine bleeding
- Review the differential diagnosis
  - Septic abortion
  - Endometritis
  - PID/TOA
  - Hemorrhagic shock
- Perform bedside ultrasound to evaluate for retained products of conception
- Recognize worsening vital signs: tachycardia, hypotension
- Prepare for resuscitation efforts with two large bore IVs and requesting crossmatched units of blood
- Work with ED colleagues to follow hospital sepsis protocols re: initiation of vasopressor support if BP refractory to IV fluids
- Prepare for and perform immediate manual vacuum aspiration
  - Recognize the importance of performing the procedure in the trauma bay, which is faster than transferring the unstable patient to the operating room
  - Have the following supplies available:
    - MVA supplies
    - Dilation and Evacuation kit
    - Uterotonics including Misoprostol, Carboprost, Methylergonovine (Methergine)
    - Anesthesia/sedation (fentanyl/midazolam)
- Prepare disposition plan
  - CT abdomen to be obtained in the ED prior to transfer pending stability
  - Consult ICU
- Review plan for antibiotics
  - Broad spectrum antibiotics: clindamycin and vancomycin
  - Consult Infectious Disease
- Demonstrate effective communication skills and workflow management with co-residents and emergency medicine colleagues

Residents who have satisfactorily completed this simulation demonstrate the following core competencies:

1. Skill in the recognition, workup, and treatment of sepsis by identifying the signs, symptoms, differential diagnosis, and need for appropriate diagnostic tests and imaging for sepsis, practicing initial management of sepsis including resuscitation and antibiotic therapy, and practicing decision-making regarding uterine evacuation aspiration abortion in the ED versus the operating room.
2. Ability to work with individuals of other professions to maintain a climate of mutual respect and clear communication
3. Skill in communication with patients and healthcare professionals in a responsive and responsible manner that supports a team approach to the promotion and maintenance of health and the prevention and treatment of disease
4. The ability to apply relationship-building values and the principles of team dynamics to perform effectively in different team roles to plan and deliver patient centered care that is safe, timely, efficient, effective, and equitable.
