# Supplementary material for: First-Trimester Abortion Complications: Simulation Cases for OB/GYN Residents in Sepsis and Hemorrhage
Source: MedEdPORTAL. 2020 Oct 16;16:10995. doi: 10.15766/mep_2374-8265.10995 (PMC7566226; doi:10.15766/mep_2374-8265.10995)
Supplement: Supplementary file 1 — Sepsis Simulation Case.docxHemorrhage Simulation Case.docxSimulation Images.docxPresimulation Didactic Lecture.pptxSepsis Critical Action Checklist.docxHemorrhage Critical Action Checklist.docxSepsis Debriefing Guide.docxHemorrhage Debriefing Guide.docxSepsis Postsimulation Debrief Didactic.pptxSepsis Pre-and Postsurvey.docxHemorrhage Pre-and Postsurvey.docx [file mep_2374-8265.10995-s001.zip › F. Hemorrhage Critical Action Checklist.docx]

**Learner(s): ___________________________ Assessor(s): ___________________________**

**Hemorrhage Simulation**

**Critical Action Checklist**

- Correctly identify dangerous bleeding
- Transition from manual to electric suction for uterine evacuation in the setting of ongoing bleeding
- Evaluate uterine bleeding using ultrasound
- Evaluate the most common etiologies of hemorrhage
  - Atony (Tone)
  - Retained products of conception (Tissue)
  - Cervical laceration (Trauma)
  - Uterine perforation (Trauma)
  - Coagulopathy (Thrombin)
- Perform uterine massage
- Administer uterotonics, recognizing contraindications
  - Methylergonovine contraindicated in patients with hypertension
  - Carboprost contraindicated in patients with asthma
- Perform cervical evaluation to evaluate for laceration
  - ask for additional instruments for proper evaluation of the cervix
- Recognize worsening vital signs: tachycardia, hypotension
- Place Foley catheter for tamponade
- Recognize need for escalation of care and transfer to operating room (OR)
- Communicate with anesthesia to draw and order labs (CBC, PTT, INR, Fibrinogen, Type and Cross)
- Communicate with staff to draw and order labs (CBC, PTT, INR, Fibrinogen, Type and Cross)
- Call OR staff to coordinate transfer to OR
- Call fellow residents to coordinate transfer to OR and any attending level backup required
- Transfer patient to the OR
  - Transfer patient to stretcher
  - Connect patient to supplemental oxygen and cardiac monitoring
  - Bring stretcher to OR
- Demonstrate effective communication skills and workflow management with co-residents, anesthesia, nursing, medical assistant, and operating room colleagues

Residents who have satisfactorily completed this simulation demonstrate the following core competencies:

1. Skill in the recognition, workup, and treatment of hemorrhage by correctly identifying hemorrhage and the most common etiologies of hemorrhage including the utility of ultrasound in diagnosis and management, demonstrating understanding of the various treatments of hemorrhage and identifying the need for transfer to the OR.
2. Ability to work with individuals of other professions to maintain a climate of mutual respect and clear communication
3. Skill in communication with patients and healthcare professionals in a responsive and responsible manner that supports a team approach to the promotion and maintenance of health and the prevention and treatment of disease
4. The ability to apply relationship-building values and the principles of team dynamics to perform effectively in different team roles to plan and deliver patient centered care that is safe, timely, efficient, effective, and equitable.
