# Supplementary material for: First-Trimester Abortion Complications: Simulation Cases for OB/GYN Residents in Sepsis and Hemorrhage
Source: MedEdPORTAL. 2020 Oct 16;16:10995. doi: 10.15766/mep_2374-8265.10995 (PMC7566226; doi:10.15766/mep_2374-8265.10995)
Supplement: Supplementary file 1 — Sepsis Simulation Case.docxHemorrhage Simulation Case.docxSimulation Images.docxPresimulation Didactic Lecture.pptxSepsis Critical Action Checklist.docxHemorrhage Critical Action Checklist.docxSepsis Debriefing Guide.docxHemorrhage Debriefing Guide.docxSepsis Postsimulation Debrief Didactic.pptxSepsis Pre-and Postsurvey.docxHemorrhage Pre-and Postsurvey.docx [file mep_2374-8265.10995-s001.zip › G. Sepsis Debriefing Guide.docx]

**Appendix G: Sepsis Debriefing Guide**

**General Questions:**

1. What do you think went well?
2. What did you find challenging?
3. Is there anything you think you should have done differently?
4. What surprised you about how things went?
5. How did you feel during this simulation?
6. How do you think communication was between team members?
7. Are there any changes you will make in how your approach your patients and their management?

**Specific Questions: Sepsis Simulation**

Review Pre Test

1. Antibiotic prophylaxis at the time of first-trimester medical abortion:

a. decreases the risk of clostridium infection

**b. is not strongly supported by data, can be offered, but is not universally recommended**

c. prevents PID and should be recommended to all patients

2.  All of the following are important initial treatments in the management of septic abortion EXCEPT:

a. Uterine evacuation

b. IV antibiotic

c. IV fluid resuscitation

d. **Packed red blood cells transfusion**

3. What are the preferred antibiotics for someone with sepsis after abortion?

a. Ampicillin/gentamicin/clindamycin

b. Ceftriaxone/doxycycline/flagyl

**c. Vancomycin/piperacillin/tazobactam/clindamycin**

d. Doxycycline/flagyl

4.  Which imaging modality is the most useful in diagnosing the etiology of pelvic pain during first-trimester pregnancy?

**a. Ultrasound**

b. CT

c. MRI

d. X-ray

5. The following findings are all suggestive of more severe sepsis or septic shock EXCEPT:

**a. Lactate <2.0**

b. Creatinine >2.0

c. Platelet count <100K

d. INR >1.5

****After this simulation we reviewed a presentation about sepsis with our Emergency Medicine Colleague see Appendix I****
