# Supplementary material for: First-Trimester Abortion Complications: Simulation Cases for OB/GYN Residents in Sepsis and Hemorrhage
Source: MedEdPORTAL. 2020 Oct 16;16:10995. doi: 10.15766/mep_2374-8265.10995 (PMC7566226; doi:10.15766/mep_2374-8265.10995)
Supplement: Supplementary file 1 — Sepsis Simulation Case.docxHemorrhage Simulation Case.docxSimulation Images.docxPresimulation Didactic Lecture.pptxSepsis Critical Action Checklist.docxHemorrhage Critical Action Checklist.docxSepsis Debriefing Guide.docxHemorrhage Debriefing Guide.docxSepsis Postsimulation Debrief Didactic.pptxSepsis Pre-and Postsurvey.docxHemorrhage Pre-and Postsurvey.docx [file mep_2374-8265.10995-s001.zip › H. Hemorrhage Debriefing Guide.docx]

**Appendix H: Hemorrhage Debriefing Guide**

**General Questions:**

1. What do you think went well?
2. What did you find challenging?
3. Is there anything you think you should have done differently?
4. What surprised you about how things went?
5. How did you feel during this simulation?
6. How do you think communication was between team members?
7. Are there any changes you will make in how your approach your patients and their management?

**Specific Questions:**

Review Pre Test

1. What is the most common cause of bleeding in first-trimester uterine aspiration?

a. Bleeding disorder

**b. Retained tissue**

c. Atony

d. Trauma

2. Which of the following medications should NOT be used for a patient with hypertension?

a. carboprost

**b. Methergine**

c. Misoprostol

d. Vasopressin

3. Where do most perforations occur in the setting of first-trimester uterine aspiration?

**a. Fundus**

b. Lower uterine segment

c. Lateral aspect of the uterus

4. The ultrasound finding of free fluid in the cul-de-sac during a uterine aspiration should raise concern for:

a. False tract

**b. Uterine perforation**

c. Hematometra

d. Anembryonic pregnancy

5. What are risk factors for hemorrhage during a first-trimester uterine aspiration?

a. Previous hemorrhage

b. Increased parity

c. Gestation greater than 10 weeks

**d. All of the above**
