# Supplementary material for: First-Trimester Abortion Complications: Simulation Cases for OB/GYN Residents in Sepsis and Hemorrhage
Source: MedEdPORTAL. 2020 Oct 16;16:10995. doi: 10.15766/mep_2374-8265.10995 (PMC7566226; doi:10.15766/mep_2374-8265.10995)
Supplement: Supplementary file 1 — Sepsis Simulation Case.docxHemorrhage Simulation Case.docxSimulation Images.docxPresimulation Didactic Lecture.pptxSepsis Critical Action Checklist.docxHemorrhage Critical Action Checklist.docxSepsis Debriefing Guide.docxHemorrhage Debriefing Guide.docxSepsis Postsimulation Debrief Didactic.pptxSepsis Pre-and Postsurvey.docxHemorrhage Pre-and Postsurvey.docx [file mep_2374-8265.10995-s001.zip › I. Sepsis Postsimulation Debrief Didactic.pptx]

## Slide 1
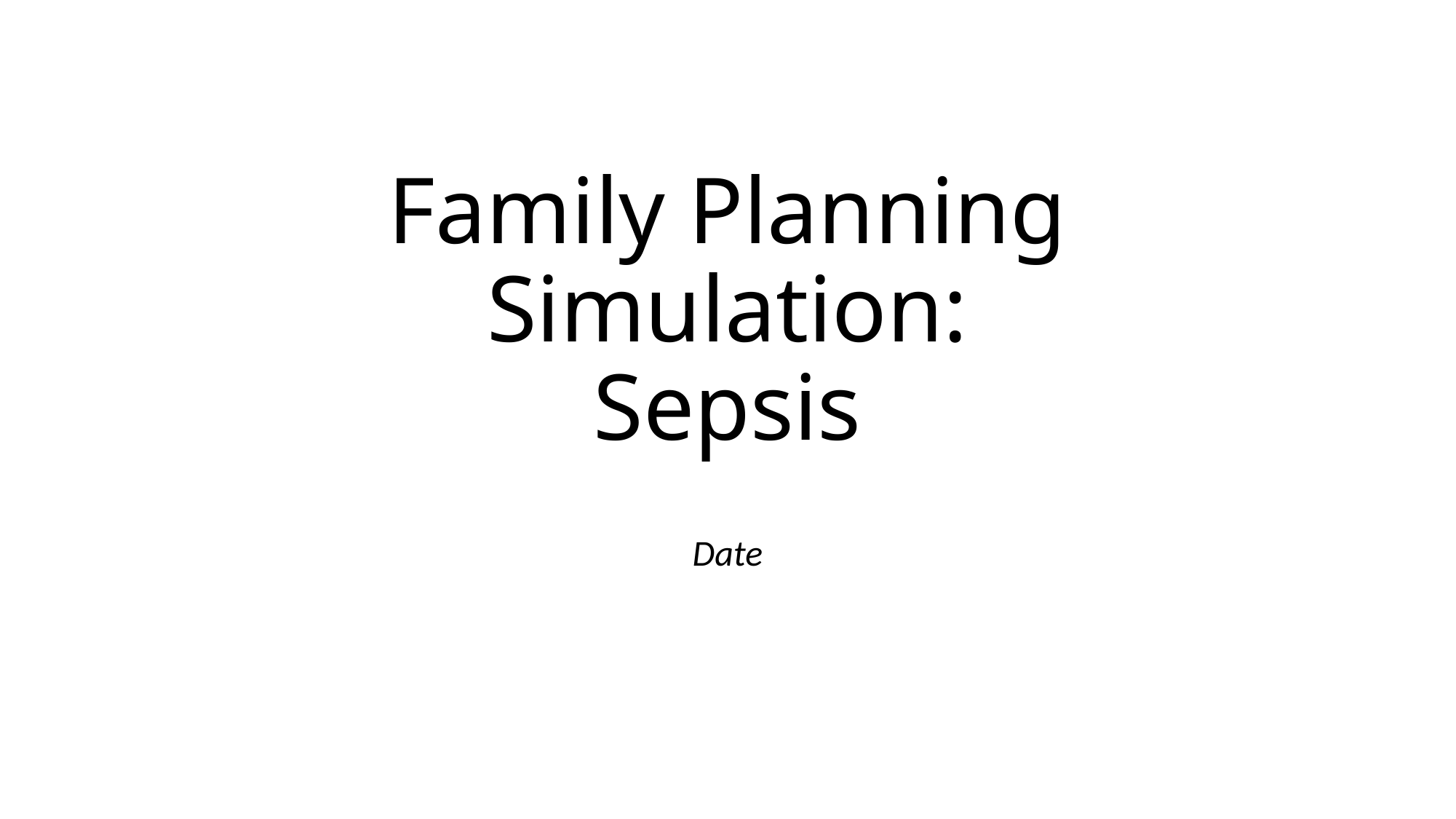

# Family Planning Simulation: Sepsis
Date

## Slide 2
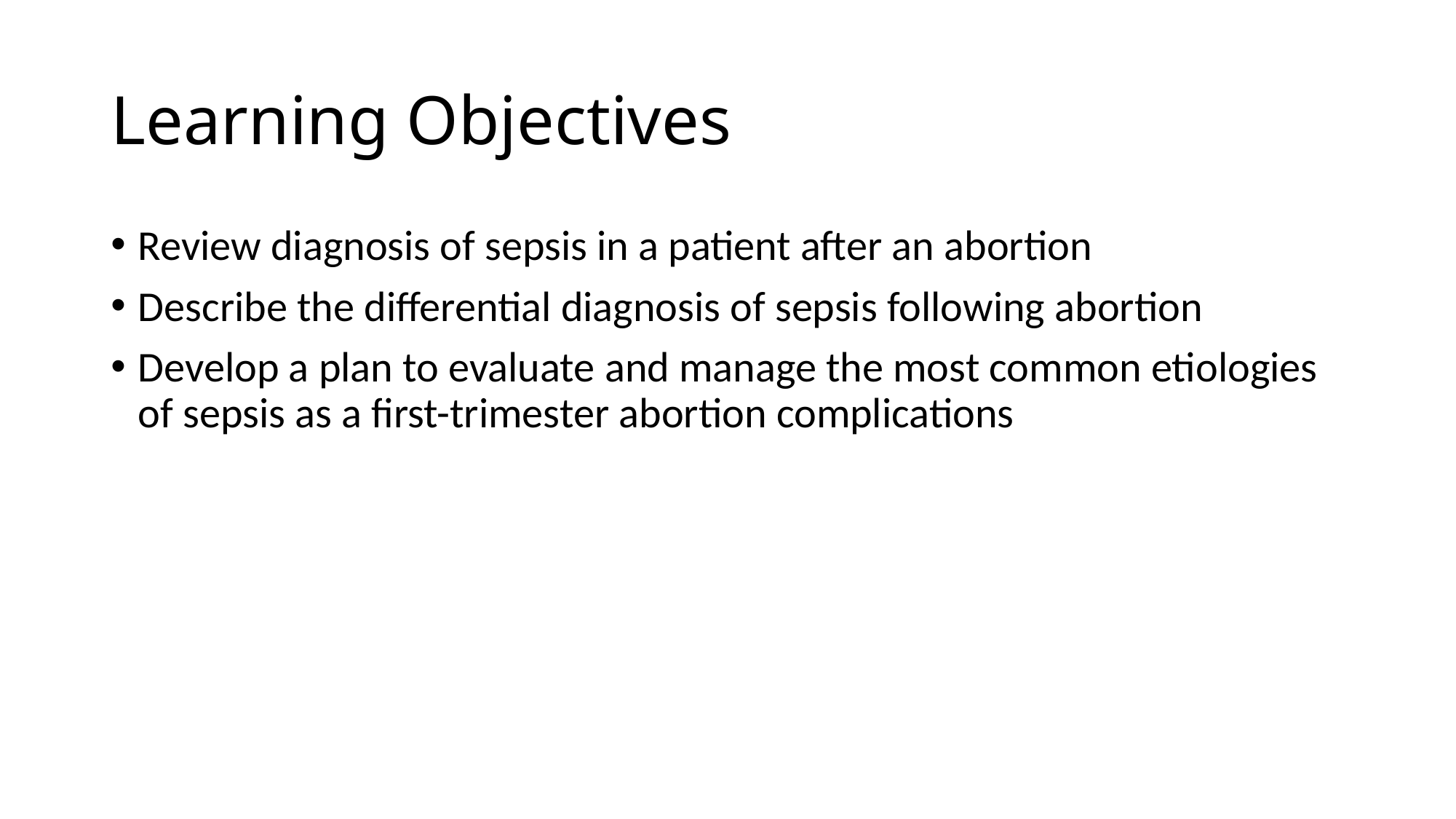

# Learning Objectives
Review diagnosis of sepsis in a patient after an abortion
Describe the differential diagnosis of sepsis following abortion
Develop a plan to evaluate and manage the most common etiologies of sepsis as a first-trimester abortion complications

## Slide 3
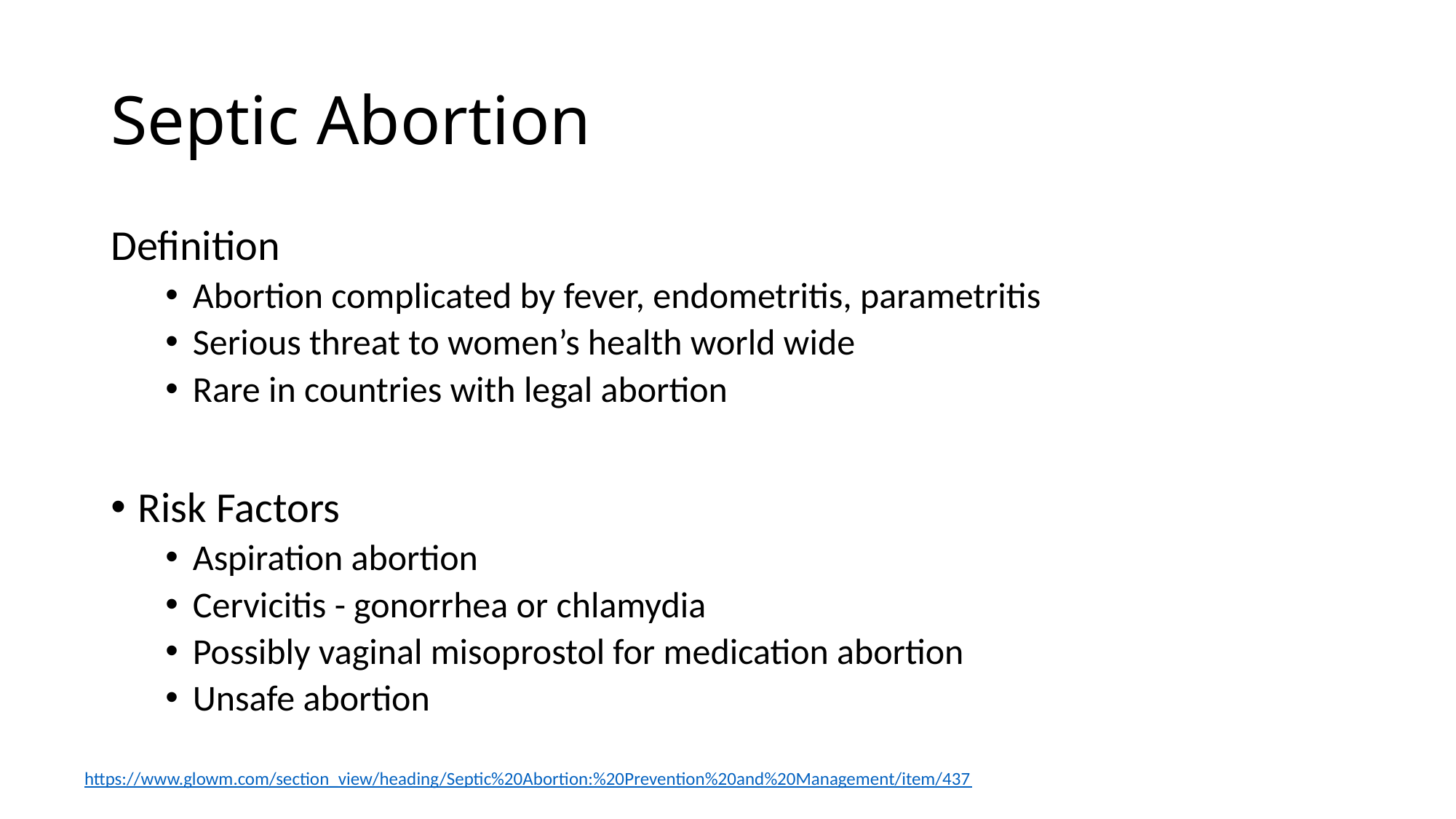

# Septic Abortion
Definition
Abortion complicated by fever, endometritis, parametritis
Serious threat to women’s health world wide
Rare in countries with legal abortion
Risk Factors
Aspiration abortion
Cervicitis - gonorrhea or chlamydia
Possibly vaginal misoprostol for medication abortion
Unsafe abortion
https://www.glowm.com/section_view/heading/Septic%20Abortion:%20Prevention%20and%20Management/item/437

## Slide 4
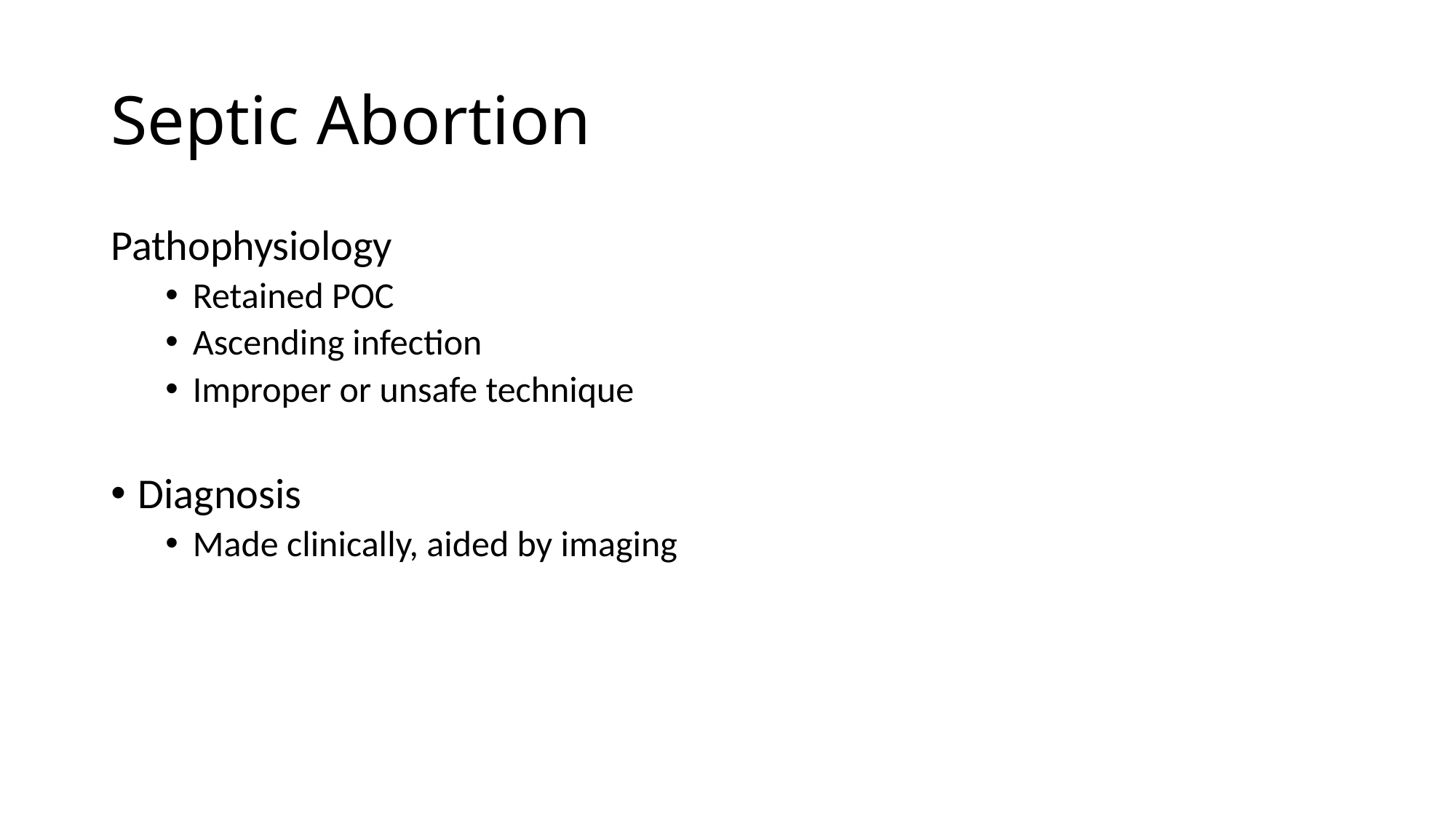

# Septic Abortion
Pathophysiology
Retained POC
Ascending infection
Improper or unsafe technique
Diagnosis
Made clinically, aided by imaging

## Slide 5
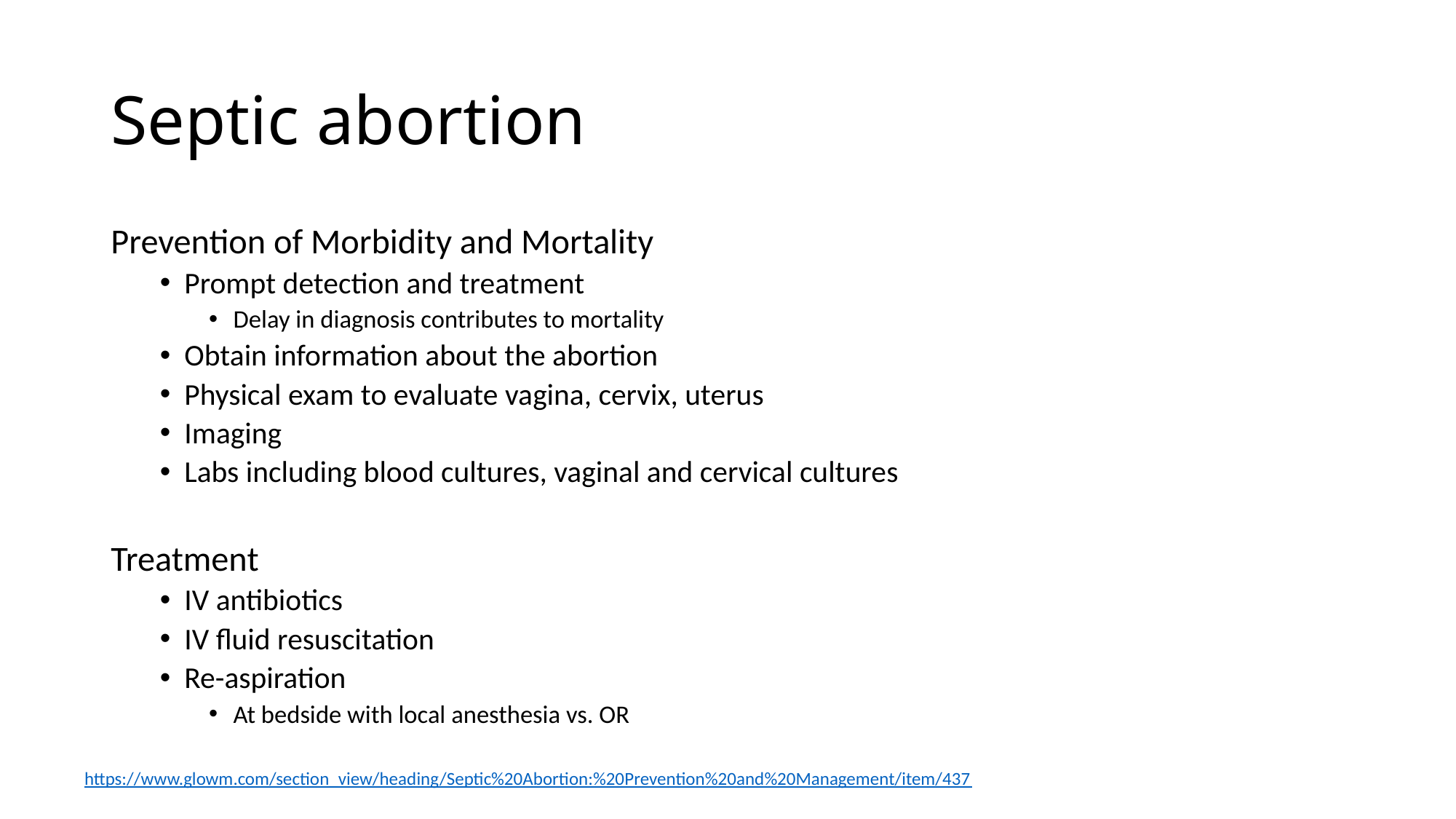

# Septic abortion
Prevention of Morbidity and Mortality
Prompt detection and treatment
Delay in diagnosis contributes to mortality
Obtain information about the abortion
Physical exam to evaluate vagina, cervix, uterus
Imaging
Labs including blood cultures, vaginal and cervical cultures
Treatment
IV antibiotics
IV fluid resuscitation
Re-aspiration
At bedside with local anesthesia vs. OR
https://www.glowm.com/section_view/heading/Septic%20Abortion:%20Prevention%20and%20Management/item/437

## Slide 6
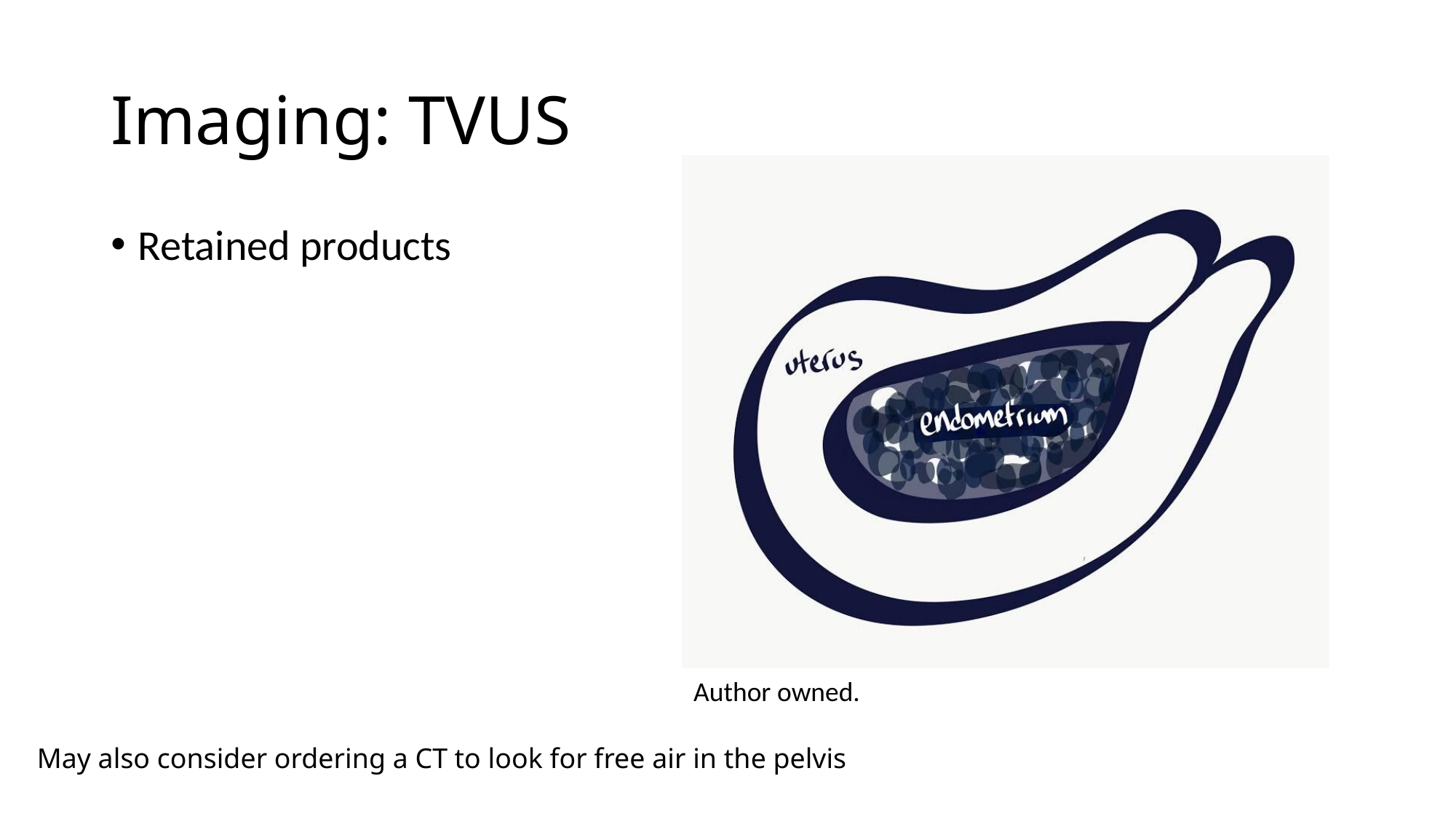

# Imaging: TVUS
Retained products
Author owned.
May also consider ordering a CT to look for free air in the pelvis

## Slide 7
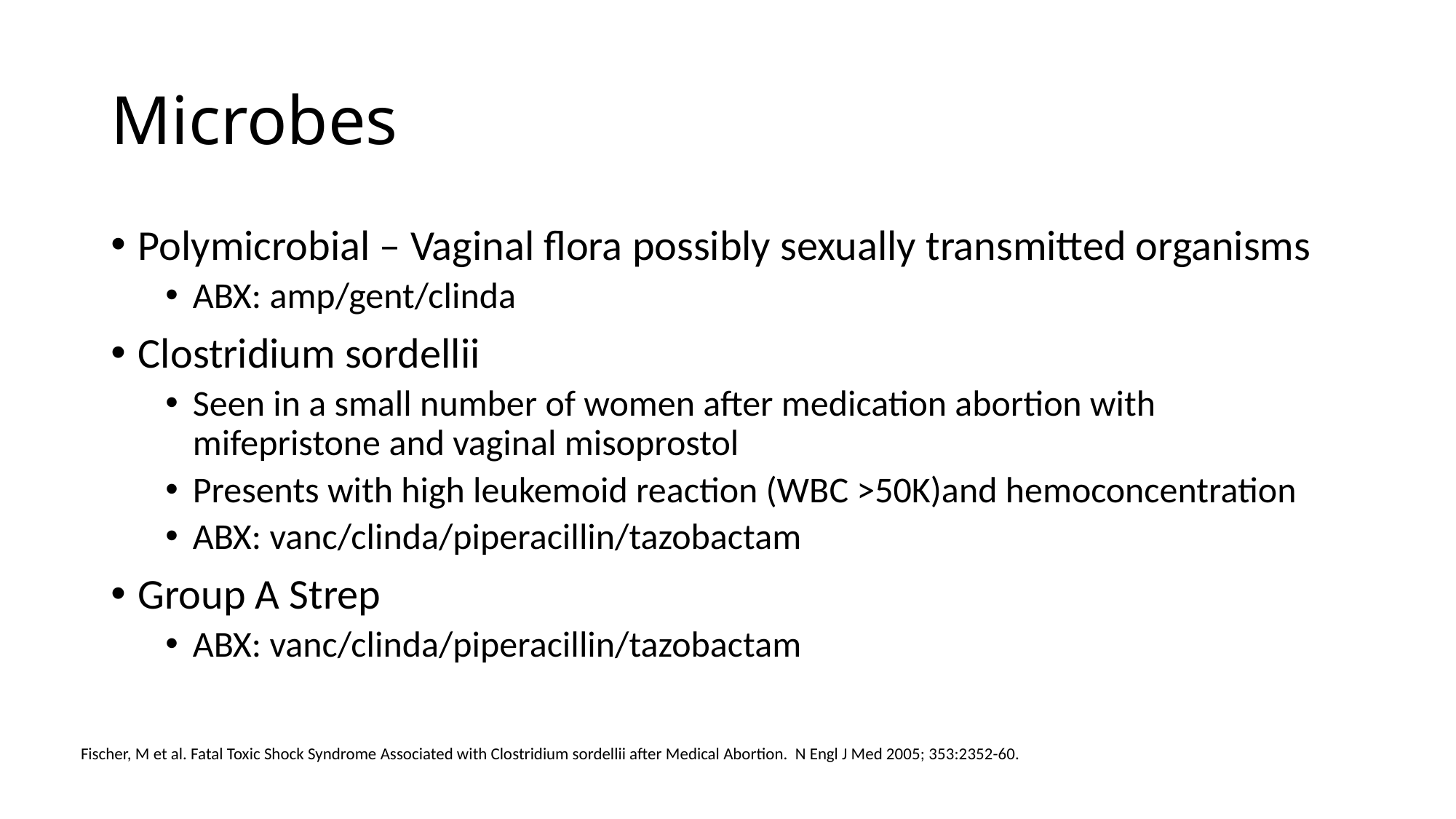

# Microbes
Polymicrobial – Vaginal flora possibly sexually transmitted organisms
ABX: amp/gent/clinda
Clostridium sordellii
Seen in a small number of women after medication abortion with mifepristone and vaginal misoprostol
Presents with high leukemoid reaction (WBC >50K)and hemoconcentration
ABX: vanc/clinda/piperacillin/tazobactam
Group A Strep
ABX: vanc/clinda/piperacillin/tazobactam
Fischer, M et al. Fatal Toxic Shock Syndrome Associated with Clostridium sordellii after Medical Abortion. N Engl J Med 2005; 353:2352-60.

## Slide 8
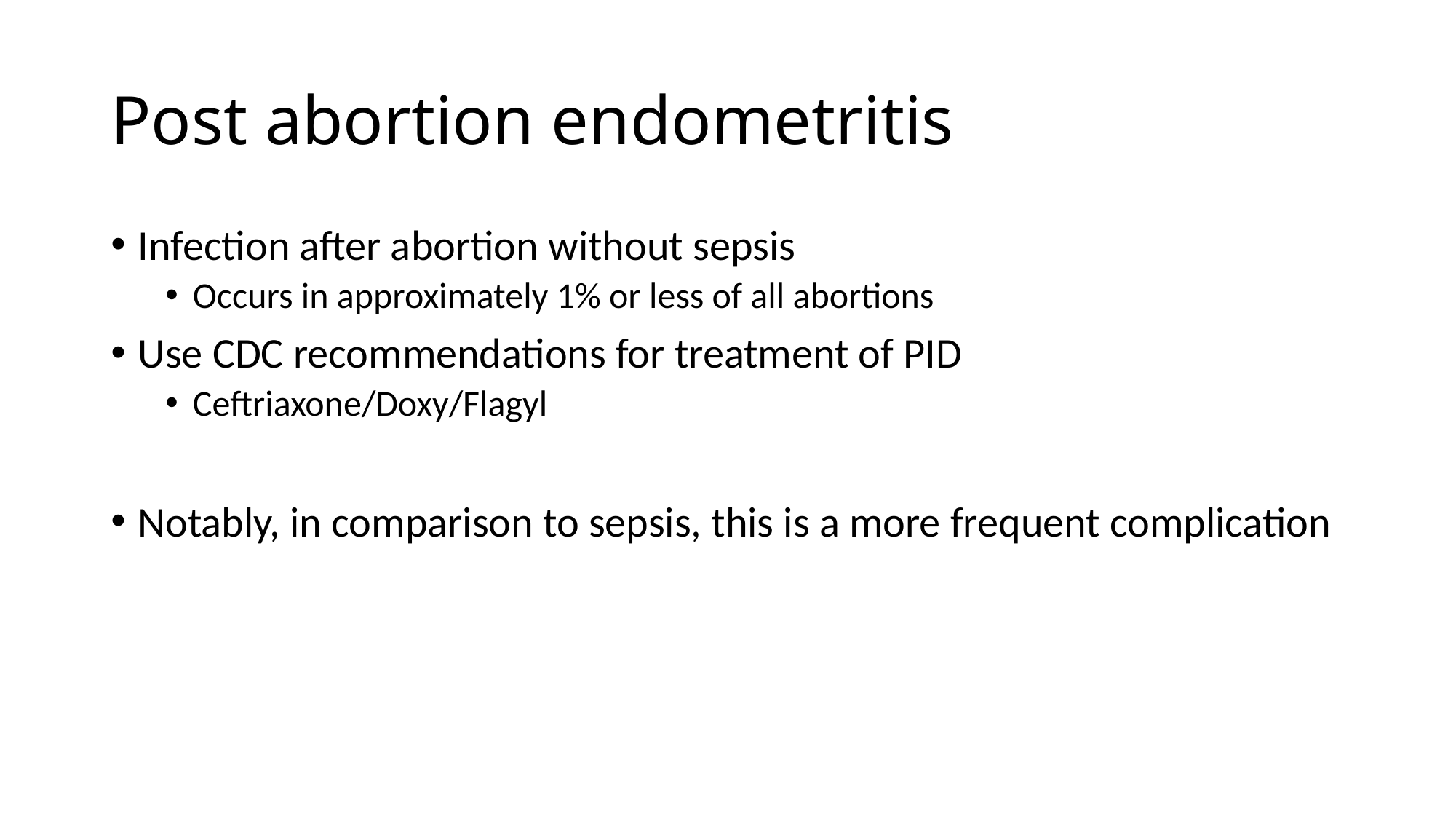

# Post abortion endometritis
Infection after abortion without sepsis
Occurs in approximately 1% or less of all abortions
Use CDC recommendations for treatment of PID
Ceftriaxone/Doxy/Flagyl
Notably, in comparison to sepsis, this is a more frequent complication

## Slide 9
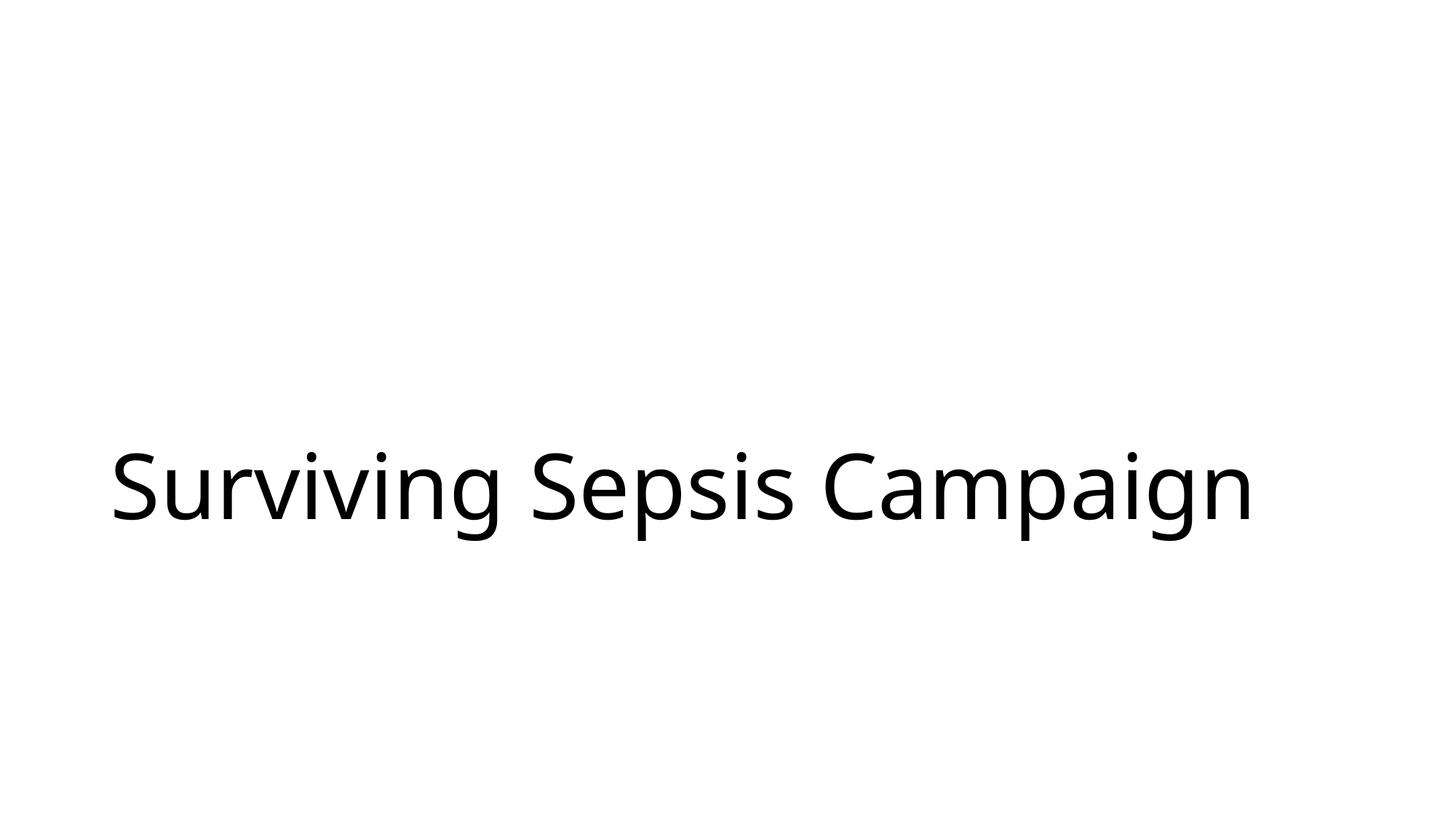

# Surviving Sepsis Campaign

## Slide 10
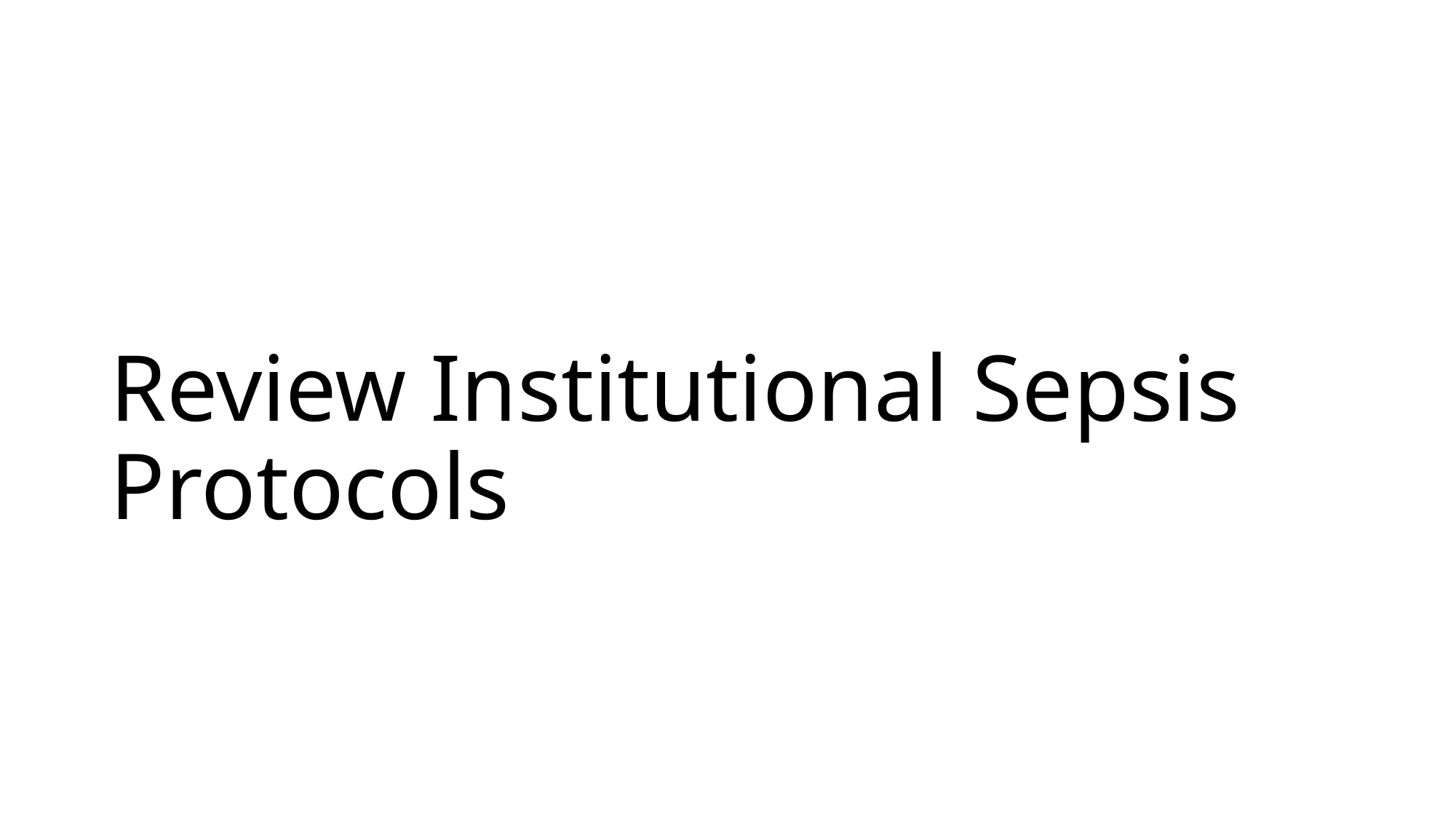

# Review Institutional Sepsis Protocols

## Slide 11
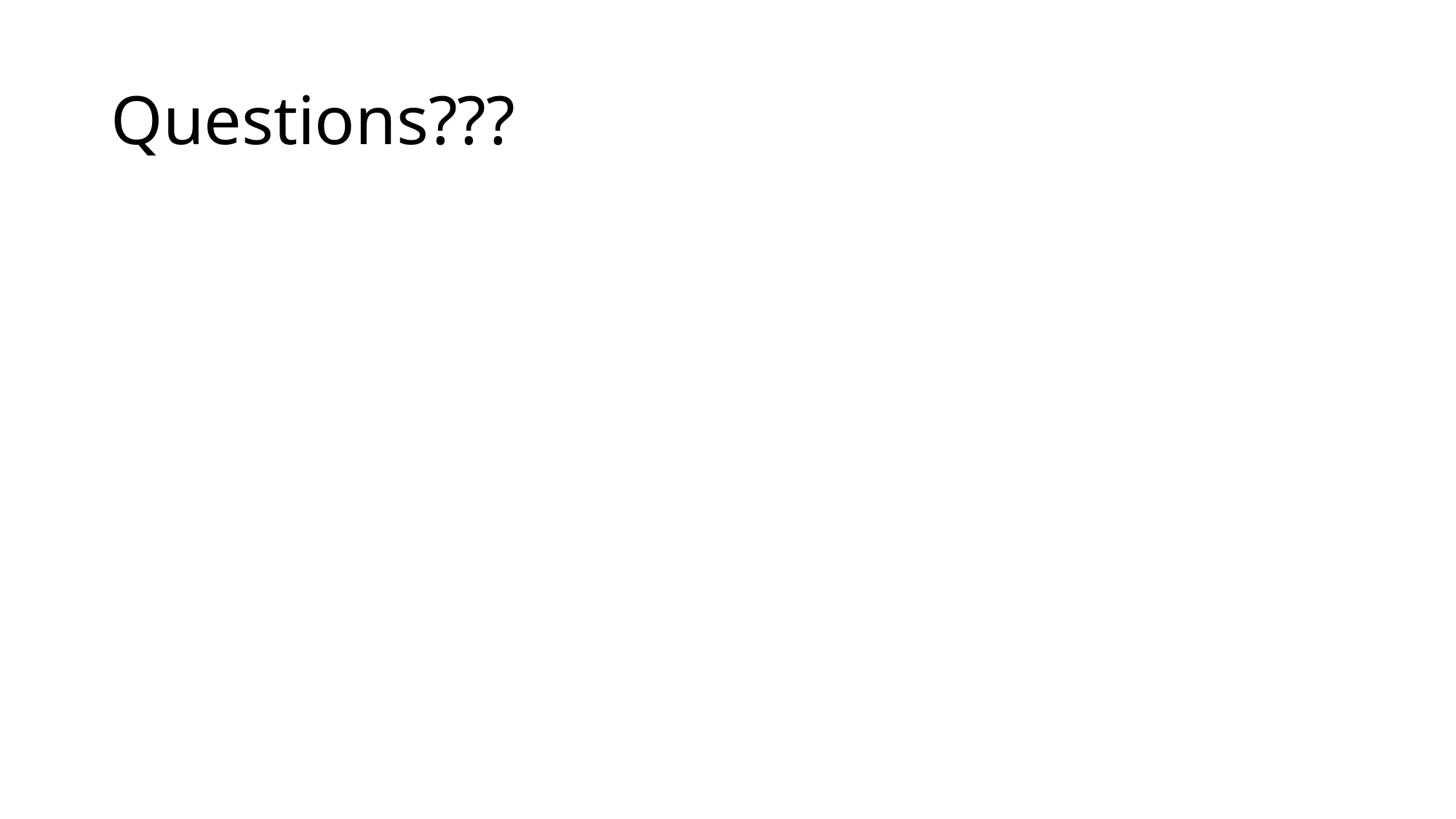

# Questions???
