# Supplementary material for: First-Trimester Abortion Complications: Simulation Cases for OB/GYN Residents in Sepsis and Hemorrhage
Source: MedEdPORTAL. 2020 Oct 16;16:10995. doi: 10.15766/mep_2374-8265.10995 (PMC7566226; doi:10.15766/mep_2374-8265.10995)
Supplement: Supplementary file 1 — Sepsis Simulation Case.docxHemorrhage Simulation Case.docxSimulation Images.docxPresimulation Didactic Lecture.pptxSepsis Critical Action Checklist.docxHemorrhage Critical Action Checklist.docxSepsis Debriefing Guide.docxHemorrhage Debriefing Guide.docxSepsis Postsimulation Debrief Didactic.pptxSepsis Pre-and Postsurvey.docxHemorrhage Pre-and Postsurvey.docx [file mep_2374-8265.10995-s001.zip › J. Sepsis Pre-and Postsurvey.docx]

**Appendix J: Pre-Survey**

**Sepsis Simulation**

Please enter your **Randomly Assigned Number** ________________

What is your residency class?

- 1. PGY-1
  2. PGY-2
  3. PGY-3
  4. PGY-4

After the Complication Workshop, please fill out the following assessment.

1. Antibiotic prophylaxis at the time of first-trimester medical abortion:

a. decreases the risk of clostridium infection

b. is not strongly supported by data, can be offered, but is not universally recommended

c. prevents PID and should be recommended to all patients

2.  All of the following are important initial treatments in the management of septic abortion EXCEPT:

a. Uterine evacuation

b. IV antibiotic

c. IV fluid resuscitation

d. Packed red blood cells transfusion

3. What are the preferred antibiotics for someone with sepsis after abortion?

a. Ampicillin/gentamicin/clindamycin

b. Ceftriaxone/doxycycline/flagyl

c. Vancomycin/ piperacillin/tazobactam/clindamycin

d. Doxycycline/flagyl

4.  Which imaging modality is the most useful in diagnosing the etiology of pelvic pain during first-trimester pregnancy?

a. Ultrasound

b. CT

c. MRI

d. X-ray

5. The following findings are all suggestive of more severe sepsis or septic shock EXCEPT:

a. Lactate <2.0

b. Creatinine >2.0

c. Platelet count <100K

d. INR >1.5

On a scale of 1 to 5, please respond using the following scale:

Disagree Strongly (1) Disagree Somewhat (2) Neutral (3) Agree Somewhat (4) Agree Strongly (5)

1. I know how to recognize sepsis in a pregnant or recently pregnant patient.

1 2 3 4 5

2. I feel competent to manage a septic patient around the time of abortion.

1 2 3 4 5

3. I am ready to take a leadership role and delegate during management of a septic patient around the time of abortion.

1 2 3 4 5

4. I can stay calm under pressure during management of a septic patient around the time of abortion.

1 2 3 4 5

**Post-Survey**

**Sepsis Simulation**

Please enter your **Randomly Assigned Number** ________________

What is your residency class?

- 1. PGY-1
  2. PGY-2
  3. PGY-3
  4. PGY-4

After the Complication Workshop, please fill out the following assessment.

1. Antibiotic prophylaxis at the time of first-trimester medical abortion:

a. decreases the risk of clostridium infection

b. is not strongly supported by data, can be offered, but is not universally recommended

c. prevents PID and should be recommended to all patients

2.  All of the following are important initial treatments in the management of septic abortion EXCEPT:

a. Uterine evacuation

b. IV antibiotic

c. IV fluid resuscitation

d. Packed red blood cells transfusion

3. What are the preferred antibiotics for someone with sepsis after abortion?

a. Ampicillin/gentamicin/clindamycin

b. Ceftriaxone/doxycycline/flagyl

c. Vancomycin/piperacillin/tazobactam/clindamycin

d. Doxycycline/flagyl

4.  Which imaging modality is the most useful in diagnosing the etiology of pelvic pain during first-trimester pregnancy?

a. Ultrasound

b. CT

c. MRI

d. X-ray

5. The following findings are all suggestive of more severe sepsis or septic shock EXCEPT:

a. Lactate <2.0

b. Creatinine >2.0

c. Platelet count <100K

d. INR >1.5

On a scale of 1 to 5, please respond using the following scale:

Disagree Strongly (1) Disagree Somewhat (2) Neutral (3) Agree Somewhat (4) Agree Strongly (5)

1. I know how to recognize sepsis in a pregnant or recently pregnant patient.

1 2 3 4 5

2. I feel competent to manage a septic patient around the time of abortion.

1 2 3 4 5

3. I am ready to take a leadership role and delegate during management of a septic patient around the time of abortion.

1 2 3 4 5

4. I can stay calm under pressure during management of a septic patient around the time of abortion.

1 2 3 4 5

5. This workshop helped prepare me to manage septic abortion in future practice.

1 2 3 4 5

6. This workshop helped prepare me to know what labs to order for a patient with a septic abortion.

1 2 3 4 5

7. This workshop helped prepare me to known when to move to the OR for a septic patient.

1 2 3 4 5

8. What is one change that you will make in your practice as a result of this workshop?

9. Further comments:
