# Supplementary material for: First-Trimester Abortion Complications: Simulation Cases for OB/GYN Residents in Sepsis and Hemorrhage
Source: MedEdPORTAL. 2020 Oct 16;16:10995. doi: 10.15766/mep_2374-8265.10995 (PMC7566226; doi:10.15766/mep_2374-8265.10995)
Supplement: Supplementary file 1 — Sepsis Simulation Case.docxHemorrhage Simulation Case.docxSimulation Images.docxPresimulation Didactic Lecture.pptxSepsis Critical Action Checklist.docxHemorrhage Critical Action Checklist.docxSepsis Debriefing Guide.docxHemorrhage Debriefing Guide.docxSepsis Postsimulation Debrief Didactic.pptxSepsis Pre-and Postsurvey.docxHemorrhage Pre-and Postsurvey.docx [file mep_2374-8265.10995-s001.zip › K. Hemorrhage Pre-and Postsurvey.docx]

**Appendix K: Pre-Survey**

**Hemorrhage Simulation**

Please enter your **Randomly Assigned Number** ________________

What is your residency class?

- 1. PGY-1
  2. PGY-2
  3. PGY-3
  4. PGY-4

After the Complication Workshop, please fill out the following assessment.

1. What is the most common cause of bleeding in first-trimester uterine aspiration?

a. Bleeding disorder

b. Retained tissue

c. Atony

d. Trauma

2. Which of the following medications should NOT be used for a patient with hypertension?

a. Carboprost

b. Methergine

c. Misoprostol

d. Vasopressin

3. Where do most perforations occur in the setting of first-trimester uterine aspiration?

a. Fundus

b. Lower uterine segment

c. Lateral aspect of the uterus

4. The ultrasound finding of free fluid in the cul-de-sac during a uterine aspiration should raise concern for:

a. False tract

b. Uterine perforation

c. Hematometra

d. Anembryonic pregnancy

5. What are risk factors for hemorrhage during a first-trimester uterine aspiration?

a. Previous hemorrhage

b. Increased parity

c. Gestation greater than 10 weeks

d. All of the above

On a scale of 1 to 5, please respond using the following scale:

Disagree Strongly (1) Disagree Somewhat (2) Neutral (3) Agree Somewhat (4) Agree Strongly (5)

1. I know how to recognize a case of hemorrhage during a first-trimester uterine aspiration.

1 2 3 4 5

2. I feel competent to manage case of hemorrhage during a first-trimester uterine aspiration.

1 2 3 4 5

3. I am ready to take a leadership role and delegate during a complication of uterine aspiration.

1 2 3 4 5

4. I can stay calm under pressure during an acute complication of uterine aspiration.

1 2 3 4 5

**Post-Survey**

**Hemorrhage Simulation**

Please enter your **Randomly Assigned Number** ________________

What is your residency class?

- 1. PGY-1
  2. PGY-2
  3. PGY-3
  4. PGY-4

After the Complication Workshop, please fill out the following assessment.

1. What is the most common cause of bleeding in first-trimester uterine aspiration?

a. Bleeding disorder

b. Retained tissue

c. Atony

d. Trauma

2. Which of the following medications should NOT be used for a patient with hypertension?

a. Carboprost

b. Methergine

c. Misoprostol

d. Vasopressin

3. Where do most perforations occur in the setting of first-trimester uterine aspiration?

a. Fundus

b. Lower uterine segment

c. Lateral aspect of the uterus

4. The ultrasound finding of free fluid in the cul-de-sac during a uterine aspiration should raise concern for:

a. False tract

b. Uterine perforation

c. Hematometra

d. Anembryonic pregnancy

5. What are risk factors for hemorrhage during a first-trimester uterine aspiration?

a. Previous hemorrhage

b. Increased parity

c. Gestation greater than 10 weeks

d. All of the above

On a scale of 1 to 5, please respond using the following scale:

Disagree Strongly (1) Disagree Somewhat (2) Neutral (3) Agree Somewhat (4) Agree Strongly (5)

1. I know how to recognize a case of hemorrhage during a first-trimester uterine aspiration.

1 2 3 4 5

2. I feel competent to manage case of hemorrhage during a first-trimester uterine aspiration.

1 2 3 4 5

3. I am ready to take a leadership role and delegate during a complication of uterine aspiration.

1 2 3 4 5

4. I can stay calm under pressure during an acute complication of uterine aspiration.

1 2 3 4 5

5. This workshop helped prepare me to manage aspiration-related hemorrhage in future practice.

1 2 3 4 5

6. This workshop helped prepare me to assess the cervix for bleeding.

1 2 3 4 5

7. This workshop w helped prepare me to perform uterine tamponade with a foley.

1 2 3 4 5

8. What is one change that you will make in your practice as a result of this workshop?

9. Further comments:
